# Symmetric Bis-Kryptopyrrol-2-Yl Squaraine as a Viscosity Probe and Dual-State Emitter

**DOI:** 10.1002/cptc.202500024

**Published:** 2025-07-07

**Authors:** Daniel D. Ta, Bong Lee, Agnieszka Jablonska, William L. Burnett, Rajveer Sagoo, Danh Pham, Luca Ceresa, Jose L. Chavez, Joseph D. Kimball, Cynthia B. Powell, Jeffery L. Coffer, Kayla N. Green, Ignacy Gryczynski, Zygmunt Gryczynski, Benjamin G. Janesko, Sergei V. Dzyuba

**Affiliations:** Department of Chemistry and Biochemistry, Texas Christian University, Fort Worth, TX 76129, USA; Department of Physics and Astronomy, Texas Christian University, Fort Worth, TX 76129, USA; Department of Physics and Astronomy, Texas Christian University, Fort Worth, TX 76129, USA; Department of Chemistry and Biochemistry, Texas Christian University, Fort Worth, TX 76129, USA; Department of Physics and Astronomy, Texas Christian University, Fort Worth, TX 76129, USA; Department of Physics and Astronomy, Texas Christian University, Fort Worth, TX 76129, USA; Department of Physics and Astronomy, Texas Christian University, Fort Worth, TX 76129, USA; Department of Physics and Astronomy, Texas Christian University, Fort Worth, TX 76129, USA; Department of Physics and Astronomy, Texas Christian University, Fort Worth, TX 76129, USA; Department of Chemistry and Biochemistry, Abilene Christian University, ACU Box 28132, Abilene, TX 79699, USA; Department of Chemistry and Biochemistry, Texas Christian University, Fort Worth, TX 76129, USA; Department of Chemistry and Biochemistry, Texas Christian University, Fort Worth, TX 76129, USA; Department of Physics and Astronomy, Texas Christian University, Fort Worth, TX 76129, USA; Department of Physics and Astronomy, Texas Christian University, Fort Worth, TX 76129, USA; Department of Chemistry and Biochemistry, Texas Christian University, Fort Worth, TX 76129, USA; Department of Chemistry and Biochemistry, Texas Christian University, Fort Worth, TX 76129, USA

**Keywords:** aggregations, density functional theories, dual-state emitters, environment-sensitive probes, molecular rotors

## Abstract

Easily accessible small organic fluorophores with multiplexing capabilities are of interest for a variety of fields. Here, a combination of experimental and computational methods reveals previously unknown, yet potentially useful properties of one of the original squaraine dyes, i.e., bis-kryptopyrrol-2-yl squaraine. The rotational flexibility of the pyrrole units around the squaraine core allows for this dye to act as a viscosity probe. In addition to the previously known fluorescence emission in solution with λ_em_ in the 550–580 nm range, this dye exhibits solid-state emission with λ_em_ at ≈800 nm. Thus, this dye is a dual-state emitter. density functional theory/time-dependent density functional theory calculations and concentration-dependent studies, in both solution and solid-state, indicate aggregated form of these dyes is responsible for the solid-state emission in the near-IR range.

## 1. Introduction

**Figure 1.**
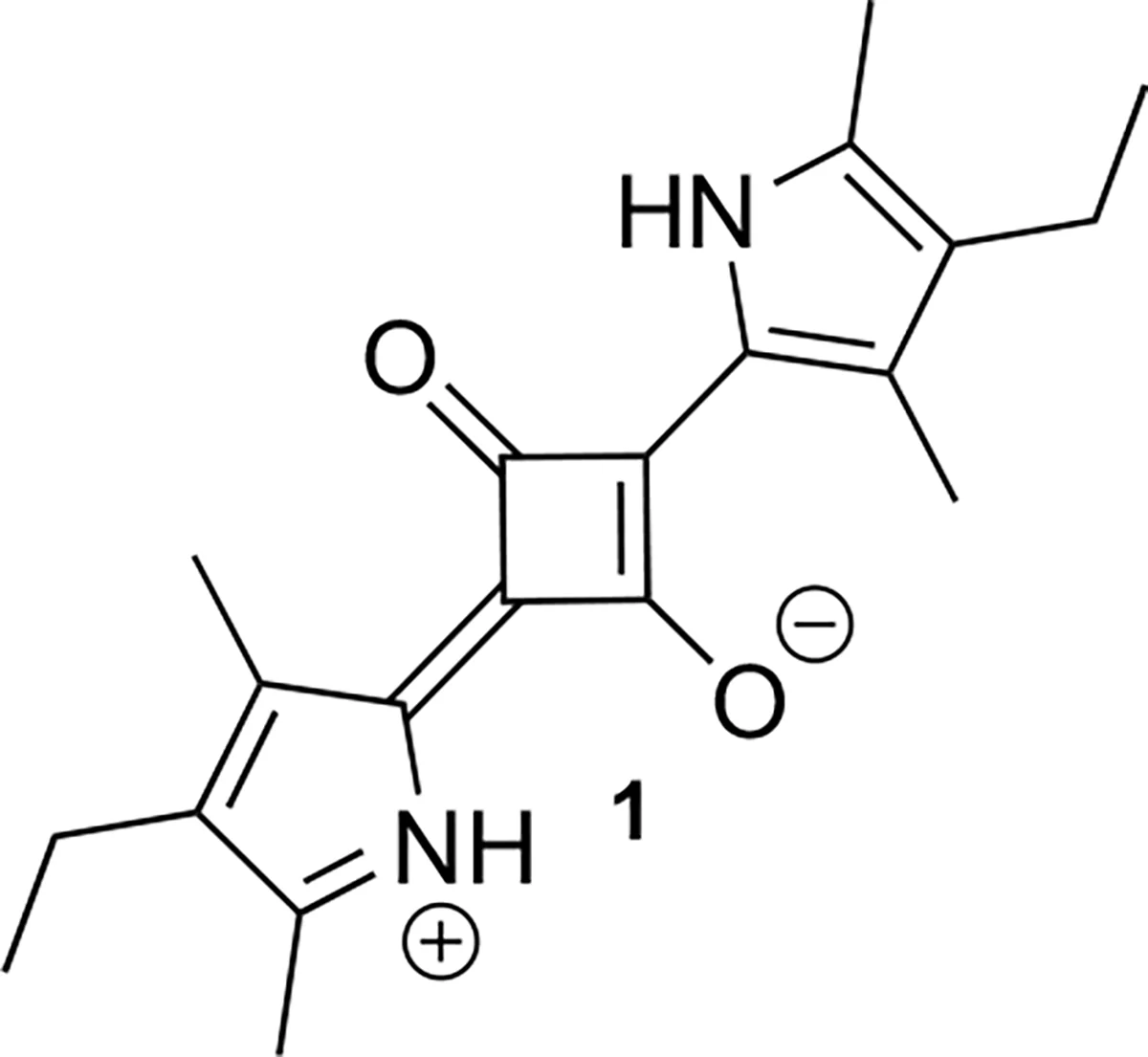
Structure of dye **1**; anti-conformer is shown.

Squaraine dyes can be categorized as “privileged” dye scaffolds due to their relative ease of synthesis, tunable photophysical properties, and a fairly diverse range of applications, which span over various areas of chemical, biological, medicinal, materials, and energy-related fields.^[1–6]^ Dye **1** (Figure 1) was one of the first reported squaraine dyes, and it was synthesized by simply heating kryptopyrrole (i.e., 3-ethyl-2,4-dimethylpyrrole) and squaric acid in ethanol or 50% acetic acid, followed by a chromatography-free isolation in 65 or 90 % yield.^[7,8]^ At that time, nuclear magnetic resonance (NMR) studies were not conducted, allegedly due to the poor solubility of **1**. It should be pointed out that poor solubility was also reported for several other symmetric aryl-containing pyrrol-2-yl squaraines (Figure S1, Supporting Information).^[9,10]^ Arguably, the aforementioned perceived limitations might have contributed to the lack of detailed photophysical studies on **1** in the following years. Recently, however, the photophysical characteristics of **1** in several solvents as well as ^1^H/^13^C NMR spectra of **1** in deuterated dimethyl-sulfoxide (DMSO-d_6_) were reported.^[11,12]^ In addition, **1** was utilized as a starting material for the synthesis of a BF_2_-containing squaraine (Figure 1S, Supporting Information), which was used as a photoinitiator for radical polymerizations.^[12]^ In view of the somewhat incomplete characterization of **1**, we decided to conduct a more detailed photophysical characterization of **1**, and in line with our interest in squaraine-based probes,^[13]^ investigate the capabilities of dye **1** as a potential environment-sensitive probe^[14]^

## 2. Results and Discussion

### 2.1. Synthesis of 1

Original synthesis of **1** was accomplished in EtOH or 50% AcOH at room temperature (Table 1).^[7,8]^ Dye **1** was also obtained using a conventional n-butanol/toluene mixture under reflux (Dean–Stark).^[12]^ During our studies on the synthesis of kryptopyrrol-2-yl semisquaraine in water,^[15]^ we observed an appreciable amount of **1** being formed, even when an excess of squaric acid was used. Thus, we looked at the possibility of forming **1** in water (Table 1). However, the reaction required a long, yet comparable to other syntheses^[8,12]^ reaction time, and recrystallization from EtOH to obtain pure product. Recently, we demonstrated that the synthesis of dye **1** could also be accomplished in a facile and efficient manner using ethylene glycol (EG) as the solvent.^[16]^ Notably, the aforementioned syntheses produced **1** as a solid with various shades of purple colors. Thus, crystallization from EtOH was done to assure homogeneity of the sample for all subsequent studies.

### 2.2. NMR Studies on Rotational Conformers of 1

Rotational isomers, i.e., anti–syn conformers, have been suggested and/or demonstrated using NMR spectroscopy for several squaraine dyes (Figure S2, Supporting Information).^[17–24]^ The formation of intramolecular hydrogen bonds was suggested to control rotameric ratios.^[17–19]^ The conversion between two conformers was induced by the addition of trifluoroacetic acid (TFA), and anti- and syn-rotamers were recorded in TFA-d.^[20]^ In some cases, squaraine’s rotamers were separated by column chromatography, yet an equilibration to the original anti/syn-mixture was noted within a day.^[21]^ It should be pointed out that for a few structurally related **1** squaraine dyes (Figure S2, Supporting Information),^[22,23]^ conformational isomers were also suggested.

From a practical perspective, conformational flexibility of squaraines is of interest due to the possibilities of using squaraine dyes as environment-sensitive probes,^[13,25]^ as well as due to some applications in materials,^[26]^ including rational design of conformer-specific supramolecular polymers.^[19]^ Thus, we investigated the spectroscopic behavior of **1** in several molecular organic solvents using ^1^H NMR spectroscopy.

**Figure 2.**
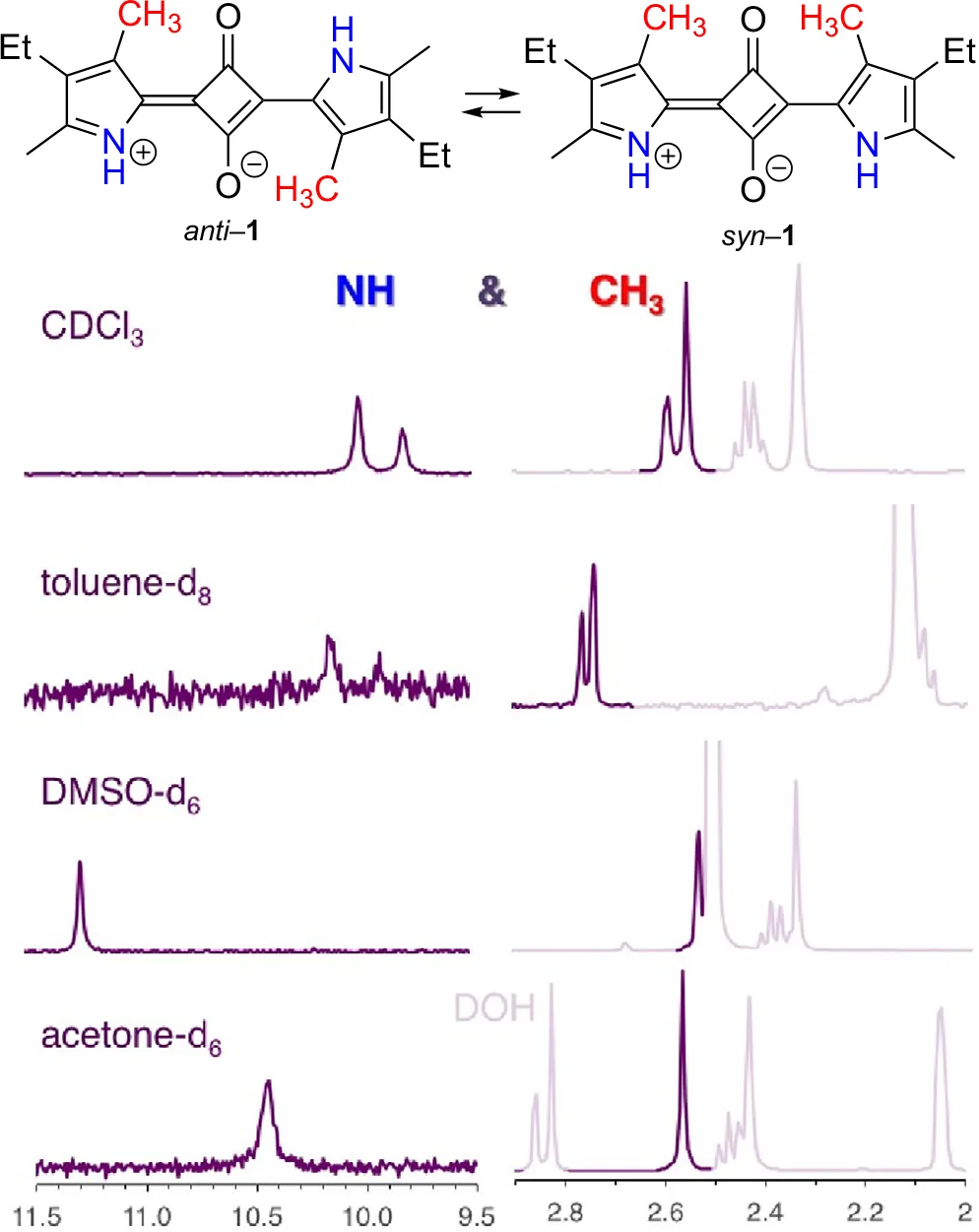
Partial ^1^H NMR spectra of **1** in different solvents. NH (blue; left) and CH_3_ (red; right) resonances are highlighted in each spectrum. See SI for complete ^1^H NMR spectra of **1** in deuterated solvents.

Specifically, in CDCl_3_, **1** exhibited two sets of resonances for the NH- and CH_3_-groups, whereas in DMSO-d_6_, only one set of resonances was observed (Figure 2; see Figure S3, Supporting Information for CH_3_-group assignment). By expanding the range of solvents, we found that distinct resonances of NH for anti- and syn-rotamers were also observed in toluene-d_8_, nitrobenzene-d_5_, and 1,2-Cl(CD_2_)_2_Cl. In contrast, a single set of resonances for the NH was observed in acetone-d_6_, CD_3_CN, and methanol-d_4_ (Figure 2 and S4, see Supporting Information for complete ^1^H NMR spectra).

In order to rationalize the effect of the solvent, we examined several solvent parameters. It appeared that in relatively nonpolar solvents, with E_T_(30) values below 40 kcal mol^−1^, i.e., CDCl_3_, toluene-d_8_, and nitrobenzene-d_5_, both rotamers were observed. However, as the E_T_(30) value of the solvent increased above 40 kcal mol^−1^, only one set of resonances for the NH- and CH_3_-protons was observed, yet no meaningful, linear correlations with E_T_(30) and other solvent parameters, such as π* and ε, were noted (Table S1). Arguably, the observation of two conformers in some solvents could simply be due to differential solvation of the anti- and syn-rotamers in those solvents as opposed to the effect of solvents’ properties (such as polarity, ability of the solvents to stabilize intramolecular H-bonding, for example).

To clarify the effect of solvents on the rotameric ratio, we conducted density functional theory (DFT) calculations (B3LYP/6–31+G(d,p)) on a homologous structure **1** (Table S2, Supporting Information), where the ethyl groups were replaced with methyl groups, in order to reduce computational costs. When continuum solvent models were considered, the chemical shifts for the NH resonances appeared to be virtually the same in both CDCl_3_ and DMSO (Table S2, Supporting Information). On the other hand, when one explicit solvent molecule was coordinated to **1** (Figure S4, Supporting Information; this approach was based on literature account^[27]^), it appeared that the NH resonances could be differentiated in CHCl_3_ and DMSO (Table S2, Supporting Information) with the resonance of the anti-rotamer being down-field from those of the syn-rotamer. This result was in good correlation with the experimental NMR results (Figure 2). Lastly, when nitrobenzene, MeOH, and acetone were used as solvating molecules, the computed NH resonances (Table S2 and Figure S5, Supporting Information) were found to be in relatively good agreement with experimental NMR data (Figure 2 and S4, Supporting Information).

Overall, the combination of ^1^H NMR and computational studies, which considered a coordinated solvent molecule, has furnished substantial sufficient evidence that both rotamers of **1** were present in all solvents. However, in some solvents (such as CHCl_3_, toluene, and nitrobenzene), differential solvation has enabled the observation of both anti- and syn-rotamers. It is of interest to note that these results are in some agreement with a literature account also indicating a somewhat unique behavior (attributed to specific solvent–solute interactions) of some squaraine dyes in aromatic and halogenated solvents (e.g., toluene, benzene, chloroform) as opposed to other solvents (e.g., dioxane, acetone, acetonitrile).^[28]^

### 2.3. Photophysical Properties of Solutions; 1 as a Viscosity Probe

The absorption and steady-state emission data of **1** in various low-viscosity molecular solvents appeared to be virtually identical to previously published data (Table S3, Figure S6, S7, Supporting Information).^[11]^ Notably, **1** was found to be nonemissive in water, while its absorption spectrum featured a broad transition with the maximum at 485 nm (Figure S8, Supporting Information), which suggested the presence of various types of aggregated species. The fluorescence emission lifetimes of **1** were also measured in the above solvents (Table S3, Figure S9, Supporting Information). Specifically, small amplitude average lifetimes (i.e., ≈40–50 ps range) were observed in solvents of higher polarity, such as MeOH and DMSO. In contrast, in less polar solvents, such as hexane and toluene, the amplitude average lifetimes were found to be higher (i.e., 0.17–1.35 ns range). In general, higher quantum yields appeared to correlate with larger lifetimes (Table S3, Supporting Information). Notwithstanding, no useful correlation between the photophysical properties of **1** and the physical properties of the tested solvents emerged (Table S4, Figure S10, Supporting Information), indicating that **1** could not be used as a solvatochromic probe.

**Figure 3.**
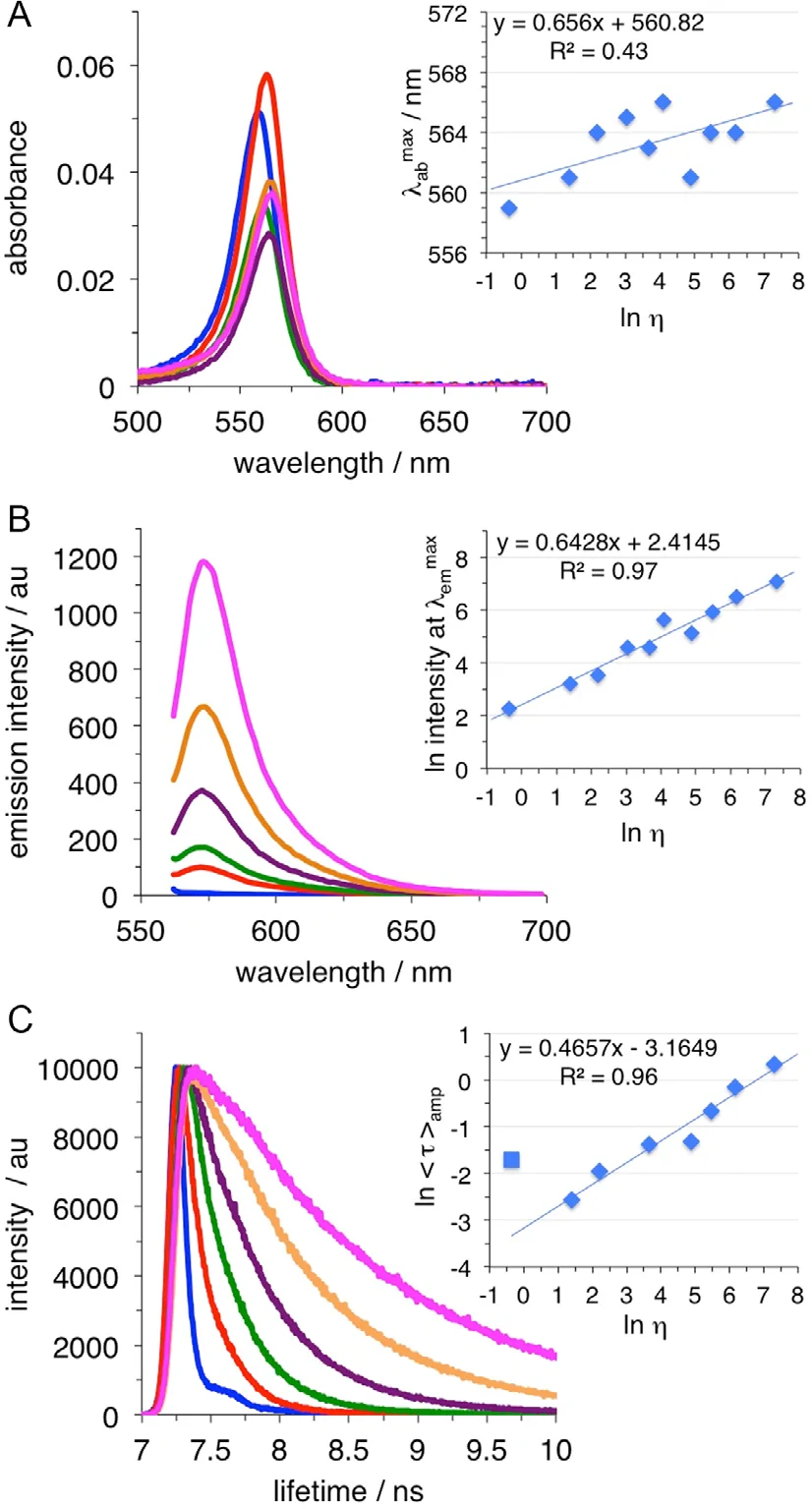
A) absorption spectra of **1** in solvents of various viscosities; inset: absorption maximum of **1** as a function of media’s viscosity; for a complete set of data see Figure S11, Supporting Information. B) Fluorescence emission spectra of **1** in solvents of various viscosities; inset: emission maximum of **1** as a function of media’s viscosity; for complete set of data see Figure S12, Supporting Information. C) Fluorescence intensity decays of **1** as a function of media’s viscosity; inset: amplitude average emission lifetime of **1** as a function of media’s viscosity (MeOH/square was not included in the correlation); for complete set of data see Figure S13, Supporting Information. Color coding: MeOH (blue), glycerol (pink), and glycerol/MeOH mixtures: 6/4 (red), 7/3 (green), 8/2 (purple), and 9/1 (orange).

On the other hand, in view of the rotational flexibility of **1** (Figure 2), we decided to investigate its capability to act as a viscosity probe. By examining the absorption spectra of **1** in solvents of varying viscosities, we noted a slight bathochromic shift, albeit with a relatively poor correlation (Figure 3A and S11, Supporting Information). On the other hand, the emission intensity of **1** showed a good, positive linear correlation with the media’s viscosity (Figure 3B and S12, Supporting Information). Importantly, the amplitude average fluorescence lifetimes of **1** also showed good correlation with the viscosity of the media (Figure 3C, Table S6, Supporting Information). Intensity average lifetimes showed a qualitatively similar correlation with the viscosity of the media (Figure S14, Supporting Information). The linearity of the plots of the emission intensity and fluorescence lifetimes versus media’s viscosity, with the slopes above 0.3, unambiguously showed a rotor-like behavior of dyes **1**.^[29,30]^ It should also be noted that, similar to other molecular rotors, **1** showed poor correlation for the low viscosity solvents, such as MeOH (Figure S15, Supporting Information).^[29,30]^

Arguably, the observed correlations that were based on fluorescence lifetimes (Figure 3C) emerged as the most useful due to the advantageous property that fluorescence lifetimes remain unaffected by variations in the probe’s concentration, unlike the intensity fluctuations of the probe or fluorophore.

### 2.4. Solid-State Emission of 1

In general, organic dyes with strong emission in the solution state tend to undergo the phenomenon of aggregation-causing quenching, resulting in the loss of emission in the solid state. On the other hand, molecules that exhibit emission in the solid state (either due to solid-state luminescence emission or aggregation-induced emission) tend to be nonemissive in solution. Therefore, small-molecule dyes that exhibit emission in both solution and the solid state, i.e., so-called dual-state emitters, are of particular interest and importance, as they have already found numerous applications in optoelectronic devices, bioimaging, sensing, and data encryption.^[31,32]^

Given the pronounced aggregation-induced quenching that typically affects the emission of squaraine dyes, the observation of solid-state emission from squaraines is relatively uncommon, with only a handful of instances reported.^[33–36]^ As such, recently disclosed dual-state emission^[37–39]^ and crystallization-induced emission^[40]^ of some squaraine dyes is an intriguing and potentially useful phenomenon. Thus, we decided to investigate the solid-state emission of **1**.

**Figure 4.**
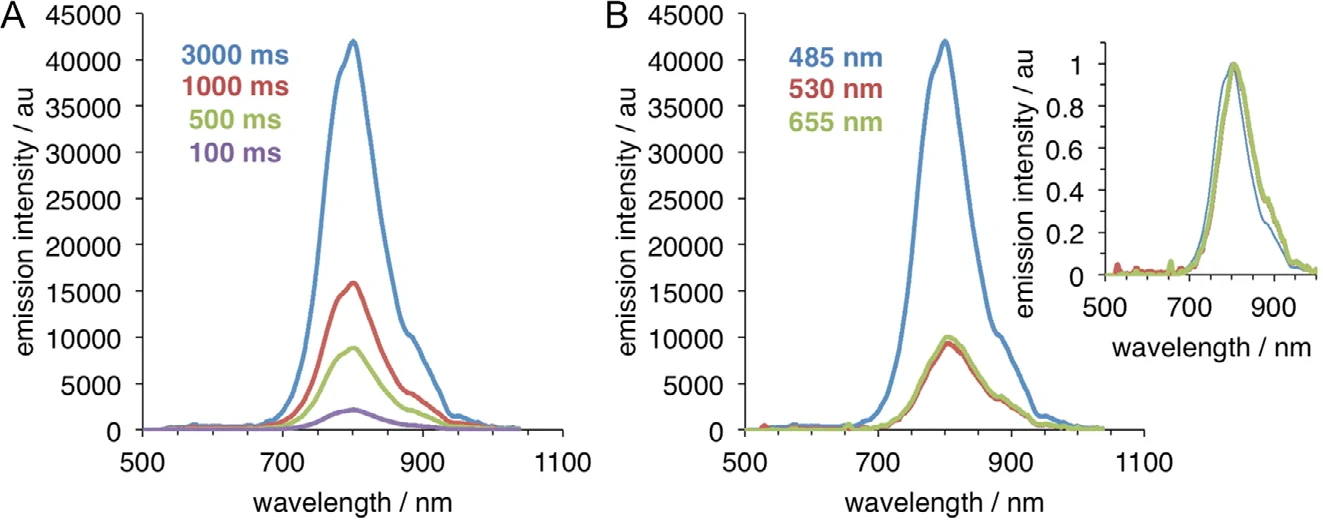
Solid-state emission spectra of dye 1 using the Ocean Optics-based system. A) Emission spectra of **1** with various integration times (3000/blue, 1000/red, 500/green, and 100/purple ms); λ_ex_ = 485 nm. B) Emission spectra at various excitation wavelengths at various excitations: λ_ex_ = 485 nm/blue, 530 nm/red, and 655 nm/green; inset: normalized emission spectra; integration time 1000 ms.

Solid-state emission of **1** was observed in the near-infrared (IR) range, with emission maximum at 790 nm (Figure 4). Also, solid-state emission of dye **1** at 790 nm was largely independent of the excitation wavelength (Figure 4B and S16, Supporting Information). It should be noted that solid-state emission of **1** was measured using both a conventional Varian spectrofluorometer as well as an Ocean Optics-based in-house built fiber optics detection system, which allowed for obtaining emission spectra of various types of samples, including solids (Figure S17, Supporting Information). Not surprisingly, due to direct excitation (the laser excites the sample directly, i.e., not through quartz/glass of the spectroscopic cell), higher quality and high intensity (as judged by a higher signal-to-noise ratio) spectra were obtained using the Ocean Optics system (Figure S16, Supporting Information). Therefore, all subsequent solid-state emission studies were done using this system.

Further, fluorescence lifetimes of **1** in the solid state were also measured, and they appeared to be substantially higher (Figure S18, Supporting Information) than those observed in the solution state (Table S3, Supporting Information). Overall, these studies indicated that either the arrangement of dye **1** or the microenvironment around **1** in the solid state must be different from that in the solution.

### 2.5. X-Ray Studies on Dye 1

**Figure 5.**
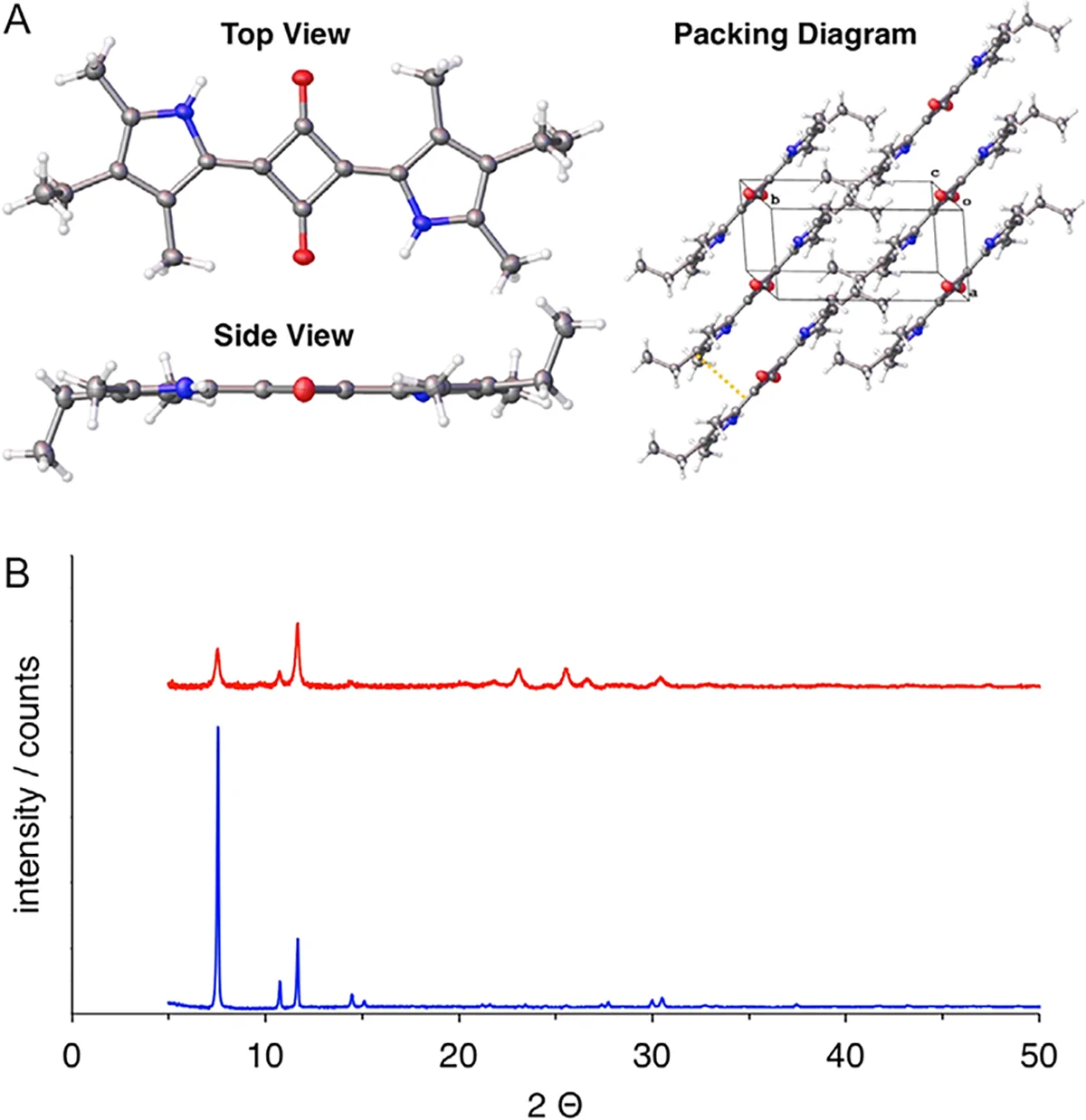
A) X-ray structure of **1**; plane to plane distance is 1.396 Å (yellow dotted line); B) powder XRD of **1** (crystalline **1** – blue; ground **1** – red). Values on the y-axis are artificially offset. Grinding was done using a mortar and pestle.

In order to understand the emission properties of **1** in the solid-state, X-ray characterization was attempted. Crystals suitable for single-crystal X-ray analysis were obtained from recrystallization from dioxane (notably, all attempts to obtain crystals that were suitable for X-ray analysis using the slow evaporation method (e.g., mixtures of MeOH-hexane, MeOH-Et_2_O, CH_2_Cl_2_-hexane, and ethyl acetate-hexane systems) were unsuccessful). Long, purple needles were analyzed, and the resulting model (Figure 5A) shows a planar, highly symmetric **1** that cocrystalizes with dioxane solvent. A distance of 1.396 Å is observed between the planes formed by molecule **1**.

Furthermore, crystallinity is also evident in the powder x-ray diffraction (XRD) analysis of **1** (Figure 5B), with prominent reflections appearing at 7.5°, 10.7°, and 11.6°. Quantitative evaluation of a sample of dye **1** that has undergone extensive grinding (Figure 5B) is consistent with a reduction of crystallinity of ≈27%. Thus, in regard to the solid-state emission, it appeared that the aggregation/stacking of monomeric **1** might be responsible for the emission at ≈800 nm.

### 2.6. Studies on the Origin of Solid-State Emission of 1

The fact that the emission profiles of **1** in solution (Figure 3B) and solid (Figure 4) states were drastically different strongly suggested that two distinct emission mechanisms were in operation. Arguably, different conformations and/or assemblies of **1** in solution and solid states could be responsible for the observed differences in emission. Thus, we conducted time-dependent density functional theory (TDDFT) calculations (B3LYP/6–31+G(d,p)) to clarify the nature of possible processes that could be responsible for the dual-state emission properties of **1**.

In general, fluorescence emission in the solid state has been attributed to either a) twisted intramolecular charge transfer (TICT) in the excited state^[41–43]^ or b) excited-state intramolecular proton-transfer (ESIPT)^[44,45]^ or c) formation of aggregates (e.g., J-aggregates).^[46,47]^

When it comes to aligning computational outcomes with experimentally observed data, several noteworthy issues merit consideration. To this end, an array of methods (Hartree-Fock, SVWN5, LC-ωPBE, wv97xd, and B3LYP) was evaluated. B3LYP was chosen as the optimal method due to accuracy in calculated redshift compared to experimental values. The accuracy of TDDFT calculations, however, encounters inherent constraints attributable to the employed approximations.^[48–50]^ In addition, only rotamers and/or assemblies of the dye **1** with nonzero oscillator strength were considered, since oscillator strength should be related to the transition probably of an electron from S_0_ to S_1_ level, i.e., oscillator strength of zero should have indicated that the particular species were nonemissive.

Computational analyses were performed on a homologous structure **1** (Figure S19, Supporting Information), where the ethyl groups were replaced with the methyl groups, in order to reduce computational costs. The anti-conformer of **1**’ had a lower energy than the syn-conformer. Yet, both conformers had similar calculated emission maximum (this was consistent with experimental results, see Figure S7, Supporting Information). On the other hand, the tautomeric forms of **1**’ had appreciably larger highest occupied molecular orbital (HOMO)–lowest occupied molecular orbital (LUMO) gaps, and as a result shorter emission. To further probe the effect of rotation and tautomerization on the emission of **1**’, we conducted TDDFT calculations.

Calculated potential energy profile for the TICT mechanism indicated that this mechanism might be responsible for the observed solid-state emission of **1**’ based on the small HOMO–LUMO gap of the twisted charge transfer form (Figure S20, Supporting Information top). However, the oscillator strength was found to decrease gradually as the dihedral angle increased (Figure S20, Supporting Information bottom), reaching zero value when two pyrrole units became perpendicular to each other, i.e., the twisted conformation was realized. Therefore, the TICT mechanism was ruled out.

Next, we investigated the possibility of the ESIPT mechanism being responsible for the bathochromic shift in emission (Figure S21, Supporting Information). Calculations predicted that the conversion of the “keto-form” of **1**’ into the “enol-form” of **1**’ was energetically unfavorable in both the ground state and excited state (Figure S21, Supporting Information), and thus, the ESIPT mechanism was ruled out as well.

**Figure 6.**
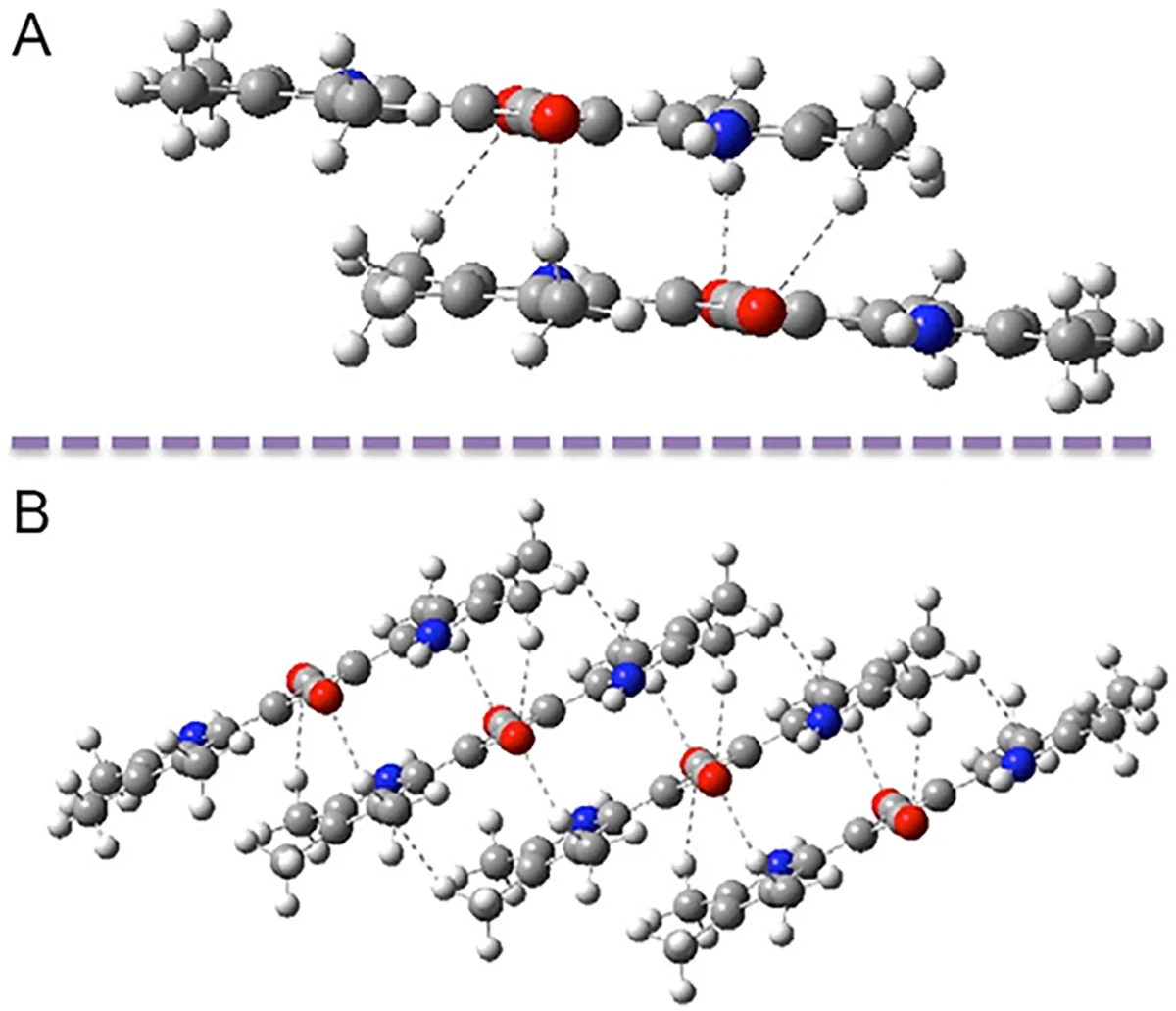
A) Computed dimer’s structure of **1**’; computed λ_em_^max^ = 585 nm. B) Computed tetramer’s structure of **1**’; computed λ_em_^max^ = 597 nm.

Finally, aggregation of **1**’ as the mechanism responsible for the solid-state emission, i.e., the formation of dimeric and high-order aggregates, was considered. Dimer of **1**’ had calculated emission maximum at 585 nm, i.e., some 60 nm redshift as compared to the calculated emission of the monomeric form of **1** (Figure 6A). Importantly, the tetrametric assembly (which was obtained by using two optimized dimeric structures, see Supporting Information for details) showed a further redshift of the emission’s maximum to 597 nm (Figure 6B). Although higher order aggregates (i.e., hexamers, octamers, etc) were not attainable due to the computational capacities of our server (see SI for details), the above results strongly indicated that aggregation of **1**’ could be responsible for the observed emission of 790 nm in the solid state. This suggestion would also be consistent with the literature account that demonstrated a red-shift of the emission of squaraine dyes upon formation of aggregates.^[51]^

Because a small twisting of the pyrrole unit appeared to lead to an increase of the emission maximum (albeit with the decreased oscillator strength, Figure S20, Supporting Information), we decided to probe the effect of peripheral rotation of the pyrrole unit on the emission of the aggregates. However, optimization of dimeric **1**’ with twisted/rotated pyrrole moieties proved to be impossible due to “dissociation” of the dimer. Thus, we used an additivity rationale to obtain predicted emission maxima for the aggregates, which have some twisting of the peripheral pyrrole units, using the data from optimized monomeric (both planar and twisted conformers) and aggregated (dimeric and tetrameric) forms. Specifically, for monomeric **1**’, the contribution of a certain degree of rotation would be Δλ_em_^max^ = λ_em_^max^ (twisted) – λ_em_^max^ (planar). The degrees of rotation were chosen to be ≤40°, for which the oscillator strength was > 0.6 (Figure S20, Supporting Information). The Δλ_em_^max^ values for 10, 20, 30, and 40 degrees of rotation were added to the base λ_em_^max^ values of dimer or tetramer to generate a set of predicted emission maxima of aggregates with partially twisted peripheral pyrroles (Figure S22, Supporting Information). The extrapolation of this set of data revealed that the solid state emission of **1**’ could depend on the aggregation of **1**’, and with higher order aggregates, which possess some degree of twisting of pyrrole units, a shift of the emission maxima to ≈700–800 nm range could be plausible.

In order to obtain additional experimental evidence that aggregates of **1** could be responsible for the solid-state emission, we conducted concentration-dependent studies. Specifically, two types of studies were envisioned: a) solution to solid study, in which **1** would be dissolved in an appropriate solvent, and the dye concentration would be gradually increased until the solubility limits are reached; and b) dispersion of **1** within a solid matrix, where solid sample of **1** would be mixed with a solid matrix at various concentrations.

**Figure 7.**
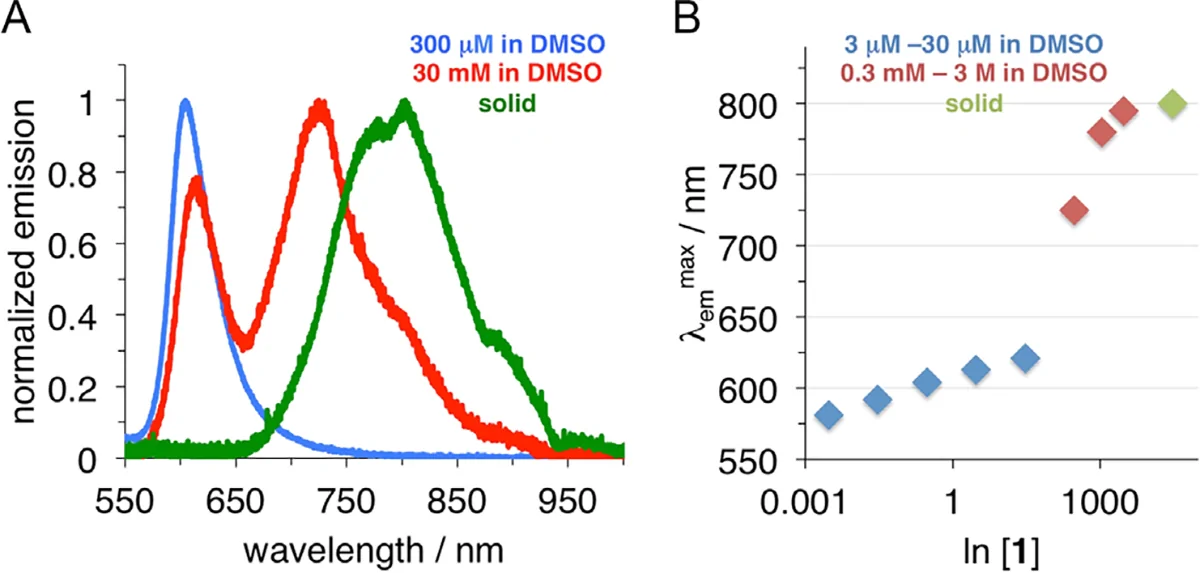
Concentration-dependent emission spectra of **1**. A) Normalized emission spectra of **1** in DMSO (at 30 μM/blue and 30 mM/red) and solid-state emission (green). B) Emission maximum of **1** as a function of concentration in DMSO solutions (blue symbols), DMSO suspensions/slushes (red symbols), and solid-state emission. λ_ex_ = 485 nm. See Figure S23, Supporting Information, for the respective emission spectra of **1**.

For the solution to solid study (Figure 7A and S23, Supporting Information), as the concentration of **1** in DMSO (i.e., a relatively nonvolatile solvent with high solubilizing ability of 1) increased, a gradual shift of the emission maximum from 580 nm to ≈790 nm was noted

(Figure 7B), which was accompanied by a decreased emission in the 580–600 nm range (Figure S23, Supporting Information).

Although homogeneous (i.e., 3–30 μM concentration) and heterogeneous (i.e., 0.3 mM–3 M concentration) samples of **1** in DMSO might not be directly compared, it could be argued that the aggregation of **1** might be responsible for the shift of the emission maxima to ≈790 nm range in the solid state. It should be pointed out that emission from high concentration solutions should be taken with caution due to inner filter effects that could distort the emission signal.^[52]^ In particular, the secondary inner-filter effect, which is caused by reabsorption of the emitted fluorescent light, when absorption and emission spectra of the dye have a significant overlap, can lead to a redshift in the emission maximum.^[53]^ In order to establish that the redshift of the emission maximum of **1** due to the formation of aggregates leading to solid-state emission (rather than the secondary inner filter effect), we measured absorption spectra of dye **1** over a wide range of concentrations (Figure S24, Supporting Information). The absorption maxima did not change appreciably (Δλ_max_ was less than 5 nm for the 3 μM to **1** M range, Figure S24, Supporting Information; along with the preservation of the overall shape of the signal, as judged by the normalized spectra), which suggested that inner filter effects were not responsible for the appearance, shape, and position of the emission bands above 650 nm. Thus, the emission at ≈800 nm should be attributed to the solid-state emission of **1**.

**Figure 8.**
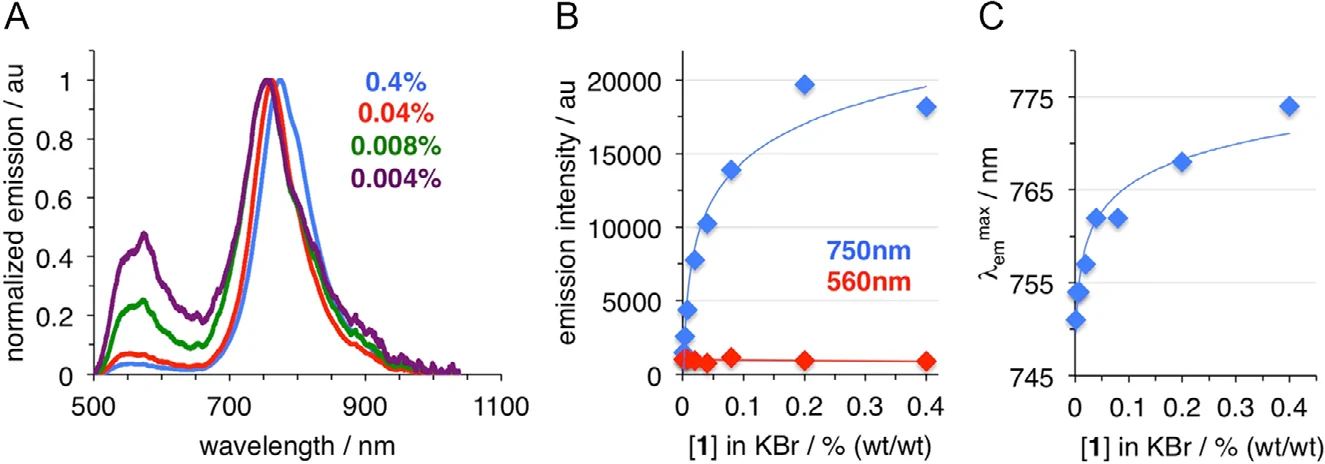
Concentration-dependent solid-state emission spectra of **1** in KBr matrix. A) Fluorescence emission spectra of **1** in KBr (concentration of 1 in wt/wt is given on the spectra); λ_ex_ = 485 nm, integration time 1000 ms. For additional spectra, see Figure S25, Supporting Information. B) Changes of emission intensity at 750 nm (blue) and 560 nm (red) as a function of concentration of **1** in KBr. C) Changes in the near-IR emission maximum as a function of concentration of **1** in KBr. The lines in **B** and **C** are added to guide the eye.

For the dispersion within the solid matrix study, **1** was mixed and thoroughly ground with solid KBr, pressed into transparent pellets, followed by the acquisition of the spectral profiles (Figure 8, S25, Supporting Information). The observed dual emission spectra (i.e., λ_em_^max^ at 560 and 750 nm; Figure 8A), with the emission maxima at 750 nm increasing as a function of increasing concentration of the dye (Figure 8B), indicated that in the KBr matrix, **1** was present in two distinct forms. Specifically, one with the emission maximum at 560 nm resembled the species that are responsible for the solution-state emission of **1**; whereas the other one, with emission at ≈750 nm, resembled the species responsible for the solid-state emission of **1**, also, as the concentration of dye **1** increased a shift to 775 nm was noted (Figure 8C). The ratio between the two forms increased in favor of the aggregated one as the concentration of **1** increased (Figure 8). Excitation-dependent emission spectra as a function of the **1** concentration in KB further supported the notion that both forms of **1** are present in the KBr matrix (Figure S26, Supporting Information).

Absorption spectra of **1** in KBr also indicated the formation of aggregates as the concentration of **1** increased (Figure S27, Supporting Information; these are somewhat comparable to the aggregates observed in water, Figure S8, Supporting Information). Importantly, from the qualitative point of view, the concentration-dependent emission profile of **1** in KBr matrix (Figure 8) resembled that observed for the solution to solid study (Figure 7).

**Figure 9.**
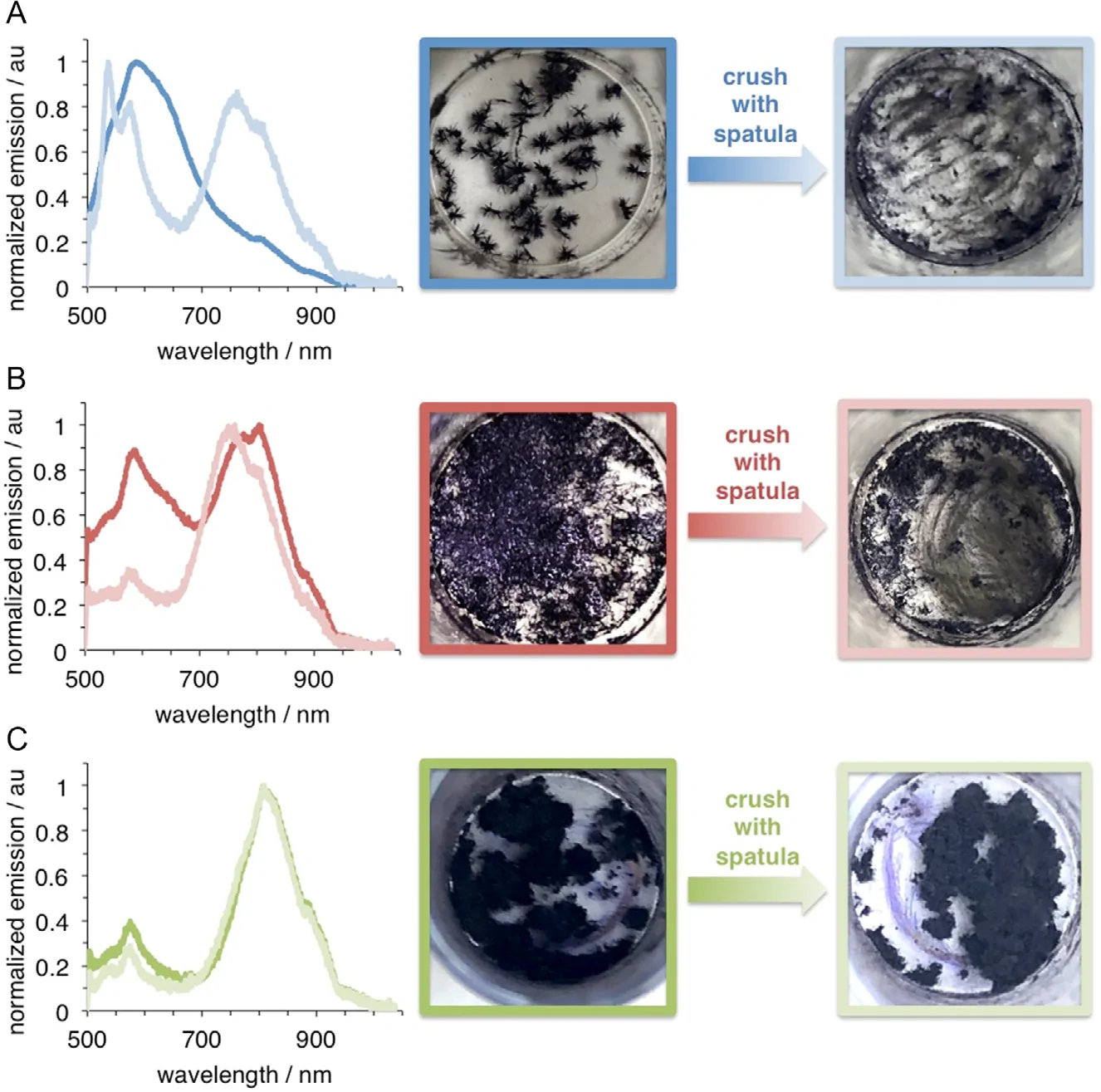
Solid-state emission spectra (left) and photographs (right) of various morphologies of dye **1** in glass vials. A) Distinctly crystalline **1** before (blue trace/blue box) and after (light blue trace/box) crushing with a spatula; B) crystalline/aggregate **1** before (red trace/box) and after (light red trace/box) crushing with a spatula; C) aggregated **1** before (green trace/box) and after (light green trace/box) crushing with a spatula. Conditions: λ_ex_ = 485 nm, front face (bifurcated fiber), laser diode; integration time 500 ms.

Finally, during extensive crystallization attempts for x-ray studies, we were also able to obtain some various morphologies of **1** upon evaporation of dioxane. The fluorescence emission of those samples (Figure 9) revealed substantial dependence on the morphology. Specifically, a distinctly visible crystalline form of **1** (Figure 9A) showed the main fluorescence emission band at ≈570 nm, with a shoulder/minor band at ≈800 nm.

Thus, emission of the crystal appeared to be similar to that observed in the solution (Figure 3B and S7, Supporting Information). On the other hand, more aggregated forms of **1** (Figure 9B,C) exhibited the main emission band in the 800 nm range (Figure 9B,C).

We reasoned that upon the disruption of the crystal structure the molecules of **1** would be forced into closer proximity to each other, thus producing a more aggregated state, which should favor the emission in the 800 nm range. Indeed, when the crystals of **1** were crushed by a spatula, the ordered arrangement of **1** appeared to be destroyed, producing a more aggregated form of **1**, which exhibited the emission in the 800 nm range (Figure 9A, light blue trace/box). In the case of the more aggregated form (Figure 9B), the crushing had a lesser effect, as only the relative intensity of the transition at 570 nm decreased (Figure 9B, light red trace/box). Consistent with these observations, no significant change in the emission spectrum of the aggregated form was observed upon crushing of the aggregates with a spatula (Figure 9C). Optical microscopy imaging of less and more aggregated forms of **1** also suggested that aggregation is responsible for the emission at ≈800 nm (Figure S28, Supporting Information).

Collectively, a combination of computational and experimental studies indicated that the solid-state emission of dye **1** at 800 nm is largely attributed to the formation of aggregated species.

## 3. Conclusion

Although dye **1** is one of the original squaraine dyes first reported over 60 years ago, many of its photophysical properties have not been established. NMR spectroscopic study revealed the conformation flexibility of dye **1**. Computational studies suggested that a solvent-specific solvation of anti- and syn-conformers, rather than the polarity of the solvent, accounted for the observation of both conformers in such solvents as chloroform, toluene, and nitrobenzene. On the other hand, in the excited state, the solvent’s viscosity was the contributing factor to the rotational isomerization of **1**. Thus, this dye appeared to be a viable viscosity probe, and its fluorescence lifetimes showed good, positive, linear correlations with the media’s viscosity. In addition, **1** exhibited previously unknown emission in the solid state, which makes **1** a dual-state emitter. Based on the computational and experimental data, aggregates of **1** were responsible for the emission in the solid state. Considering that **1** could be obtained in a facile and efficient manner, these novel properties could put this dye into a toolbox of fluorescent probes for various applications.

## Supplementary Material

SI Material

Supporting information for this article is available on the WWW under https://doi.org/10.1002/cptc.202500024

## Figures and Tables

**Table 1.** Synthesis of dye 1 in various solvents.

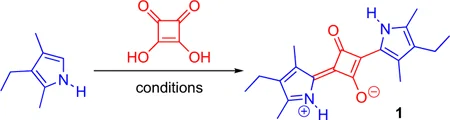			
Entry^a)^	Solvent	Reaction time/temperature	Yield, %

1	EtOH	1 & 12 h/Δ & rt	90^b)^
2	EtOH	7 h/reflux	77
3	n-BuOH/PhCH_3_	7 h/120 °C	60^c)^
4	H_2_O	7 h/reflux	58
5	EG^d)^	15 min/150 °C	90^e)^

a) [squaric acid]/[kryptopyrrole] = 1/2; 0.1 M.

b) ref. [7] reaction was performed in 90 % Ethanol (or 50% AcOH), product was obtained as a hydrate.

c) ref. [11].

d) EG – ethylene glycol.

e) ref. [15].

## Data Availability

The data that support the findings of this study are available in the supplementary material of this article.
