# Supplementary material for: Symmetric Bis-Kryptopyrrol-2-Yl Squaraine as a Viscosity Probe and Dual-State Emitter
Source: ChemPhotoChem. Author manuscript; Available in PMC 2026 Sep 22. (PMC13593347; doi:10.1002/cptc.202500024)
Supplement: Supplementary file 1 [file NIHMS2203796-supplement-SI_Material.pdf]

# SUPPORTING INFORMATION

# Symmetric bis-kryptopyrrol-2-yl squaraine as a viscosity probe and dual-state emitter

Daniel D. Ta,[a] Bong Lee,[b] Agnieszka Jablonska,[b] William L. Burnett,[a] Rajveer Sagoo,[b] Danh Pham,[b] Luca Ceresa,[b] Jose L. Chavez,[b] Joseph D. Kimball,[b] Cynthia B. Powell,[c] Jeffery L. Coffer,[a] Kayla N. Green,[a] Ignacy Gryczynski,[b] Zygmunt Gryczynski,*[b] Benjamin G. Janesko,*[a] Sergei V. Dzyuba*[a]

[a] – Department of Chemistry and Biochemistry, Texas Christian University, Fort Worth TX 76129, USA

[b] – Department of Physics and Astronomy, Texas Christian University, Fort Worth TX 76129, USA

[b] – Department of Chemistry and Biochemistry, Abilene Christian University, ACU Box 28132, Abilene, TX 79699, USA

## TABLE OF CONTENT


Materials and methods……………………………………………………………………………............ 4

Synthesis of dye **1**……………………………………………………………………........................ 5

Figure S1. General structures of squaraine dyes and dyes related to dye **1**………………….. 6

Figure S2. Several squaraine dyes with reported 1H NMRs of conformational isomers…….. 7

Figure S3. Partial HMBC spectrum of **1** with assignment of relevant Hs and Cs…………….. 8

Figure S4. Overlaid $^{1}$H NMR spectra of the NH region of **1** in various deuterated solvents… 9

Figure S5. Computed structures of conformers of dye **1'** with coordinated molecule of the solvent……………………………………………………………………………………... 10

Figure S6. Absorbance spectra of **1** in some organic solvents………………………………… 11

Figure S7. Fluorescence emission spectra of **1** in some organic solvents.…………………… 12

Figure S8. Absorbance spectrum of **1** in water……………………………...……………... 13

Figure S9. Fluorescence intensity decays of **1** in some organic solvents……………….…..… 14

Figure S10. Absorption and emission maxima of **1** as a function of solvents' parameters...... 17

Figure S11. Absorbance spectra of **1** in solvents of various viscosities…………..………… 18

Figure S12. Fluorescence emission spectra of **1** in solvents of various viscosities……..…… 19

Figure S13. Fluorescence intensity decays of **1** in solvents of various viscosities..……..…… 20

Figure S14. Intensity average lifetime of **1** as a function of solvents' viscosity……………….. 25

Figure S15. Amplitude average lifetimes of dye **1** as a function of viscosity of low-viscosity organic solvents………………………………………………………………………………. 26

Figure S16. Solid-state emission spectra of **1** using various systems and conditions…..…… 27

Figure S17. OCEANOPTICS system set-up…………………..…………………...…………… 28

Figure S18. Fluorescence intensity decays of **1** in the solid state……..……………………..… 29

Figure S19. Computed energies of ground state structures and HOMO–LUMO of various conformers and tautomers of dye **1'**……………..……………………...………... 30

Figure S20. Computed energy profile of TICT state and oscillator strength of **1'**…………… 31

Figure S21. Computed energy profile of ESIPT **1'..**……………………………...……..…. 32

Figure S22. Predicted emission of aggregates of **1'** as a function of pyrrole twisting angle… 33

Figure S23. Concentration-dependent emission spectra of **1** in DMSO……………….……… 34

Figure S24. Concentration-dependent absorption spectra of **1** in DMSO....….……………….. 35

Figure S25. Concentration-dependent emission spectra of **1** in KBr.…………………………… 36

Figure S26. Excitation dependent emission spectra of **1** in KBr……………..……..………… 37

Figure S27. Concentration dependent absorbance spectra of **1** in KBr………………………. 38

Figure S28. Normalized emission spectra of dye **1** and the respective optical microscopy images……………………………………………………………………………………… 39

Table S1. Solvent parameters and NH– and $CH_3$–groups resonances in the respective solvents……………………………………………………………………………………... 40

Table S2. Computed energies and chemical shifts of NH-resonances of *anti*- and *syn*-conformers of dye **1'** using various solvent models……………………………………… 41

Table S3. Photophysical properties of dye **1** in various organic solvents…………………..… 42

Table S4. Solvent properties………………………………………………………………….. 42

Table S5. Average fluorescence lifetimes of dye **1** in solvents of various viscosities……….. 43

$^1$H and 2D NMR spectra of dye **1**……………..…………………………………………… 44

X-ray characterization data of dye **1**……………….………………………….……………… 51

References……………………………………………………………………………............... 55

## MATERIALS AND METHODS

All reagents and solvents were purchased from the commercial sources (Sigma-Aldrich, Acros, and TCI) and were used as received. NMR spectra were acquired on a Bruker Ascend 400 (400 MHz) spectrophotometer, and the chemical shifts are reported in ppm ($\delta$) from the residual solvent peaks or TMS/tetramethylsilane (0.00 ppm). Multiplicities are reported as: s – singlet, t – triplet, q – quartet.

SPECTROSCOPIC METHODS:

For spectroscopic measurements stock solutions of non-symmetric squaraine dyes were prepared in dioxane (1 mM solution), and used within 48 hours. The UV-Vis absorption spectra were measured with a Cary 60 UV-Vis spectrophotometer (Agilent Technologies), with a resolution of 1 nm using 1 cm quartz cell. The fluorescence spectra were measured with a Cary Eclipse fluorescence spectrometer (Varian Inc.) using 1 cm quartz cell with a resolution of 1 nm. Fluorescence measurements were carried out as follows: excitation and emission width slits were 5mm and 5mm; samples were excited at 560 nm; PMT detection voltage set to 740V. The fluorescence emission spectra were corrected for the amount of absorber light according to: $I_0(1 - 10^{-Ab})$. Fluorescence lifetimes were measured on a FluoTime 200 fluorometer (PicoQuant, GmbH.) using a 485 nm diode laser. The fluorometer was equipped with an ultrafast microchannel plate detector (MCP) from Hamamatsu, Inc. The fluorescence lifetimes were measured in the magic angle condition and data analyzed using FluoFit4 program from PicoQuant, GmbH (Germany) using multi-exponential fitting model. For all viscosity measurements, the samples were subjected to gentle heating (using heat gun) and vortexing cycles to assure homogenous dispersion of the dye in the solvents of high (i.e., $\eta$ > 10 cP) viscosity.

OCEANOPTICS SYSTEM:

For the description/schematics of the fiberoptics detection system see Figure S17.

COMPUTATIONAL METHODS:

All calculations were performed using the Gaussian 16 electronic structure package.[S1] Kohn-Sham DFT[S2] and the 6-31+G(d.p)[S3] basis sets were used for calculations. All optimized geometries of **1** conformers are confirmed to be local minima with no negative eigenvalues of the vibrational Hessian. For solvated $^1$H NMR simulations the SMD continuum solvent model (with or without explicit solvent molecule attached) was used.[S4] NMR shieldings were evaluated using gauge-including atomic orbitals where chemical shifts were computed relative to the absolute shielding of TMS calculated at the same level of theory.[S4] Absorption and emission spectra were computed using the first optically allowed excited state, that is the first state with non-negligible transition dipole moment > $10^{-3}$ au.

Aggregate structures, i.e., dimeric and tetrameric assemblies of **1**, were obtained by stacking optimized monomeric structures **1**, followed by optimization procedure described above. Regretfully, attempts to calculate aggregates larger than four units resulted in time-out errors with cluster computing capabilities.

SINGLE CRYSTAL X-RAY:

Data for the crystal structure was collected on a crystal cooled to 150K using the Cu source on a Rigaku SuperNova diffractometer equipped with an ATLAS S2 CCD detector.

X-RAY POWDER DIFFRACTION:

X-ray powder diffraction was performed on a Rigaku Smartlab SE with a copper x-ray source (l = 1.5406 Å).

OPTICAL MICROSCOPY

Optical microscopy images were obtained using a light microscope (Axioskop 40, Zeiss), and images were taken by a digital camera (AxioCam MRc, Zeiss) and processed using a manufacturer provided software (AxioVision AC 4.5, Zeiss). Samples were placed in between glass slides for visualization.

**SYNTHESIS OF DYE 1.**

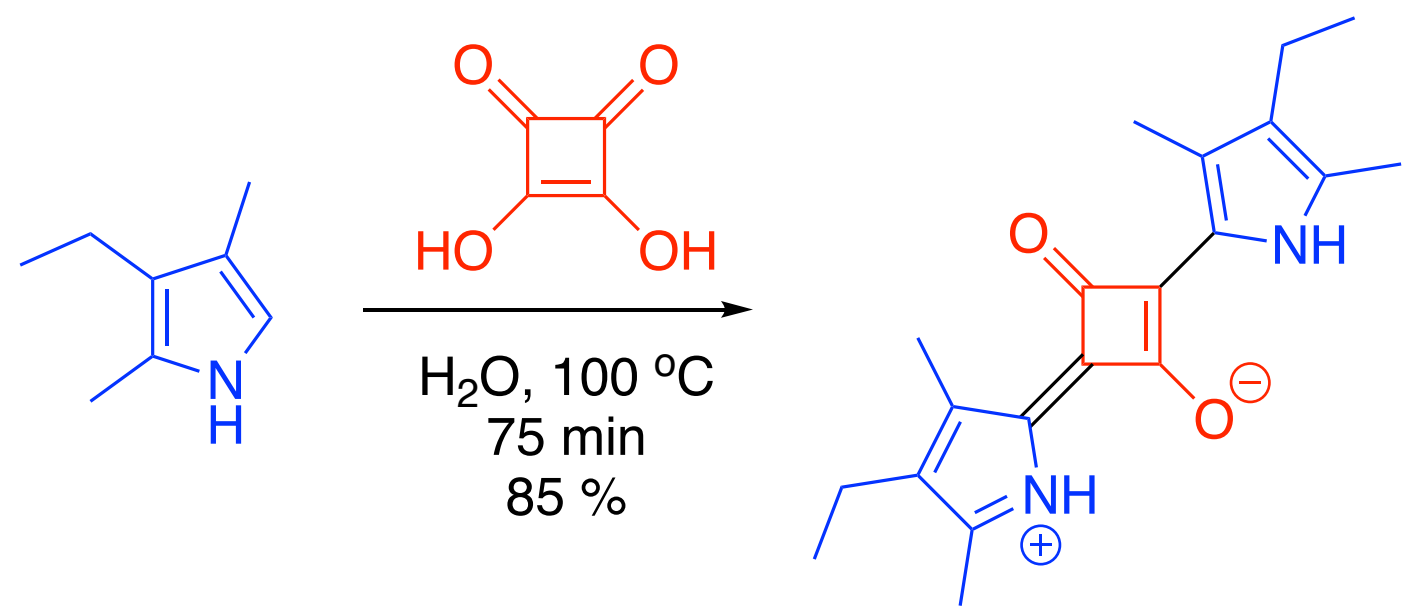


*Synthesis of **1** in EtOH:* A round bottom flask was charged with a stirring bar, squaric acid (155 mg, 1.36 mmol), EtOH (13 ml), and kryptopyrrole (0.37 ml, 2.74 mmol). The reaction flask was fitted a reflux condenser, and refluxed under stirring for 7 h, cooled to room temperature, and filtered to give **1** as a purple solid (390 mg, 77 % yield).

*Synthesis of **1** in n-BuOH/PhCH$_3$ (adopted from ref. S5):* A round bottom flask was charged with a stirring bar, squaric acid (210 mg, 1.84 mmol), n-BuOH (9.0 ml), PhCH$_3$ (9.0 ml), and kryptopyrrole (0.5 ml, 3.7 mmol). The flask was fitted with the Dean-Stark apparatus, reflux condenser. The reaction mixture was reflux for 7 h, cooled to room temperature, filtered, and the solid was washed with EtOH (10 ml) to give **1** as a purple solid (457 mg, 38 % yield).

*Synthesis of **1** in $H_2O$:* A screw cap pressure vial was successively charged with a stirring bar, squaric acid (84 mg, 0.74 mmol), water (4.0 ml), and kryptopyrrole (0.2 ml, 1.48 mmol). Next, the vial was placed into 100 $^{o}$C beads bath under stirring for 7 hours. The reaction mixture turned pink with a minute, followed by the formation of dark purple coagulated precipitate, and finally heavy purple precipitate. The vial was cooled to around room temperature, and the mixture was filtered, washed with hot water (10 ml), and the solid was suspended in EtOH (5 ml), heated, cooled and filtered to give **1** as a purple solid (276 mg, 58 % yield).

*Synthesis of **1** in ethylene glycol:* A screw cap pressure vial was successively charged with a stirring bar, squaric acid (23 mg, 0.20 mmol), ethylene glycol (2.4 ml), and kryptopyrrole (0.07 ml, 0.4 mmol). Next, the vial was placed into 150 $^{o}$C beads bath under stirring for 15 min. Next, the vial was cooled to room temperature, and the slushy mixture was poured into water (20 ml), heated with a heat gun, and filtered. The solid was washed with hot water (20 ml x 2), and dried under vacuum to give **1** as a purple solid (59 mg, 90 % yield).

$^1$H NMR (400 MHz, DMSO-d$_6$)[S5] δ: 11.25 (s, 2H), 2.54 (s, 6H), 2.36 (q, $J$ = 7.6 Hz, 4H), 2.33 (s, 6H), 1.03 (t, $J$ = 7.6 Hz, 6H).

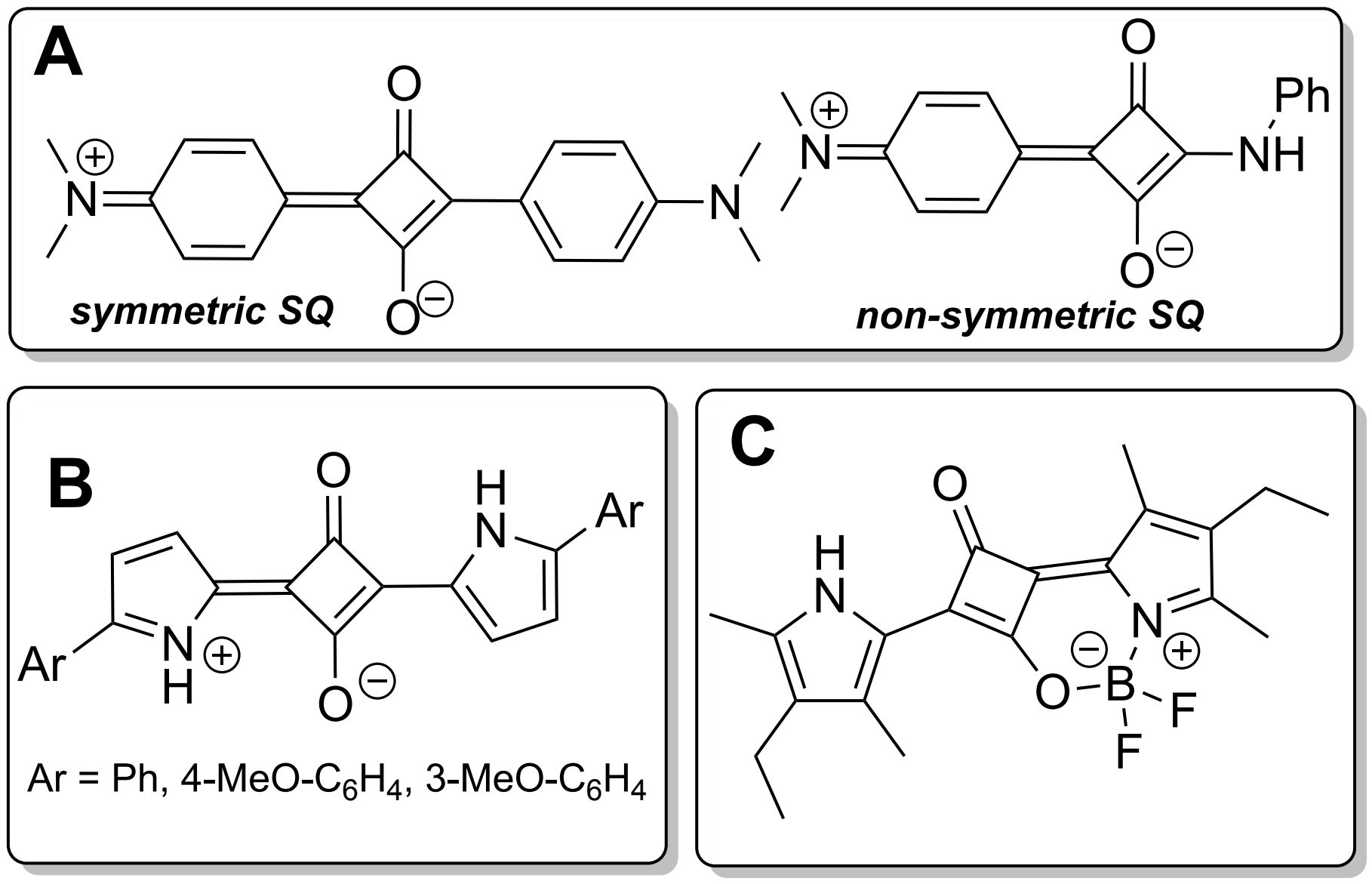


**Figure S1**. **A**: general structures of symmetric and non-symmetric squaraine (SQ) dyes; **B**: aryl-containing pyrrol-2-yl SQ dyes; **C**: $BF_2$-complexed SQ dye **1**.

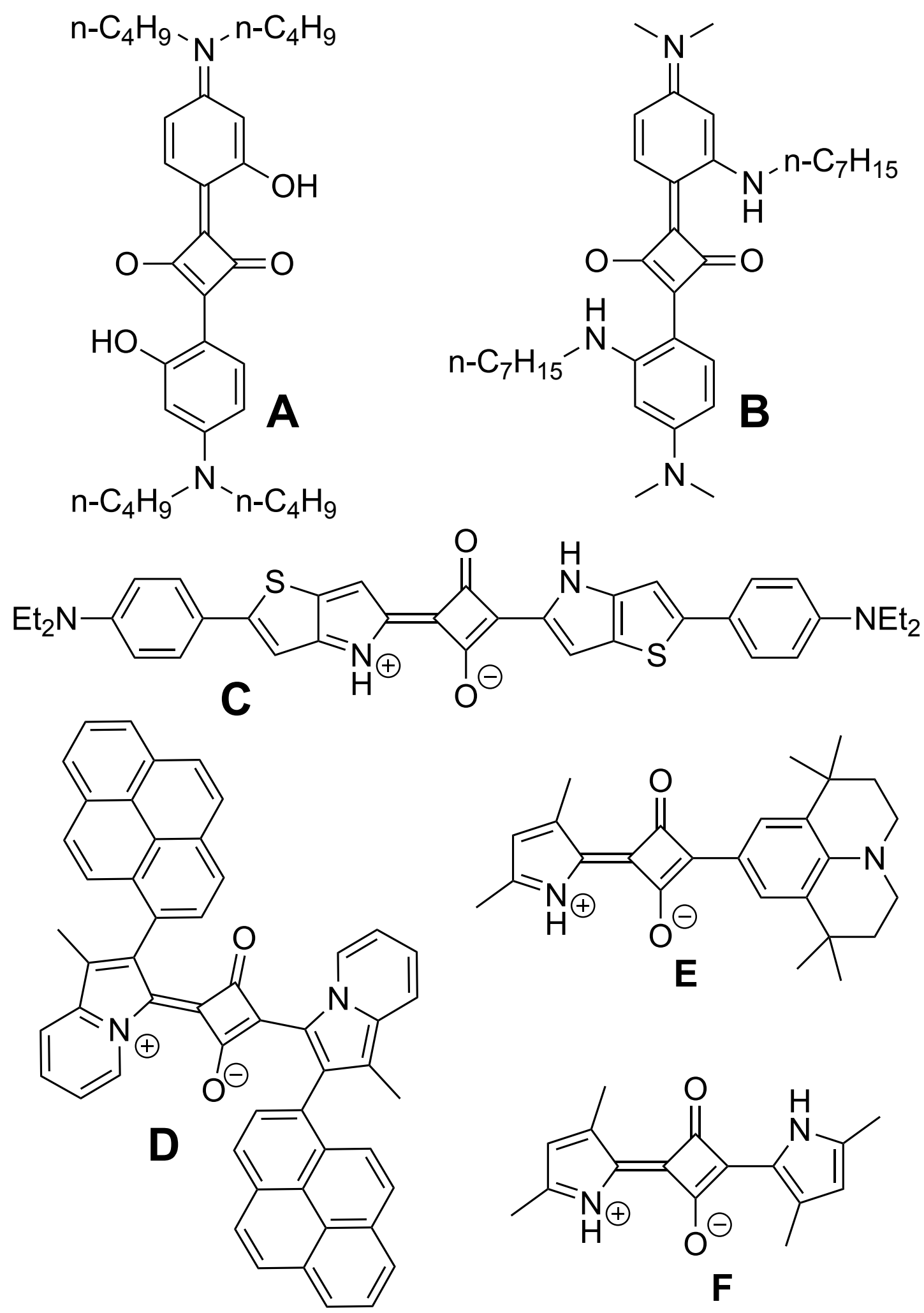

**Figure S2**. Several squaraine dyes with reported $^1$H NMRs of conformational isomers. Only major, anti-isomer is shown for each symmetric squaraine dye.

The formation of intramolecular hydrogen bonds between the amide and hydroxyl groups and the oxygen of the squaraine four-membered ring was suggested to control rotameric ratios (structures **A** and **B**).[S6–S8] The conversion between these two conformers was induced by the addition of trifluoroacetic acid (TFA), which was presumed to disrupt intramolecular hydrogen bonding.[S8]

Both *anti-* and *syn*-rotamers were recorded for dye **C** in TFA-d.[S9]

The rotamers of squaraine **D** were separated by column chromatography, yet an equilibration to the original *anti*/*syn*-mixture was noted within a day.[S10]

For structurally related to dye **1** squaraine dyes, *e.g.*, **E** and **F**,[S11,S12] conformational isomers were also suggested. Importantly, the $^1$H NMR spectrum of **F** showed the presence of separate resonances for *anti*- and *syn*-conformers (although they were not described as such).[S12]

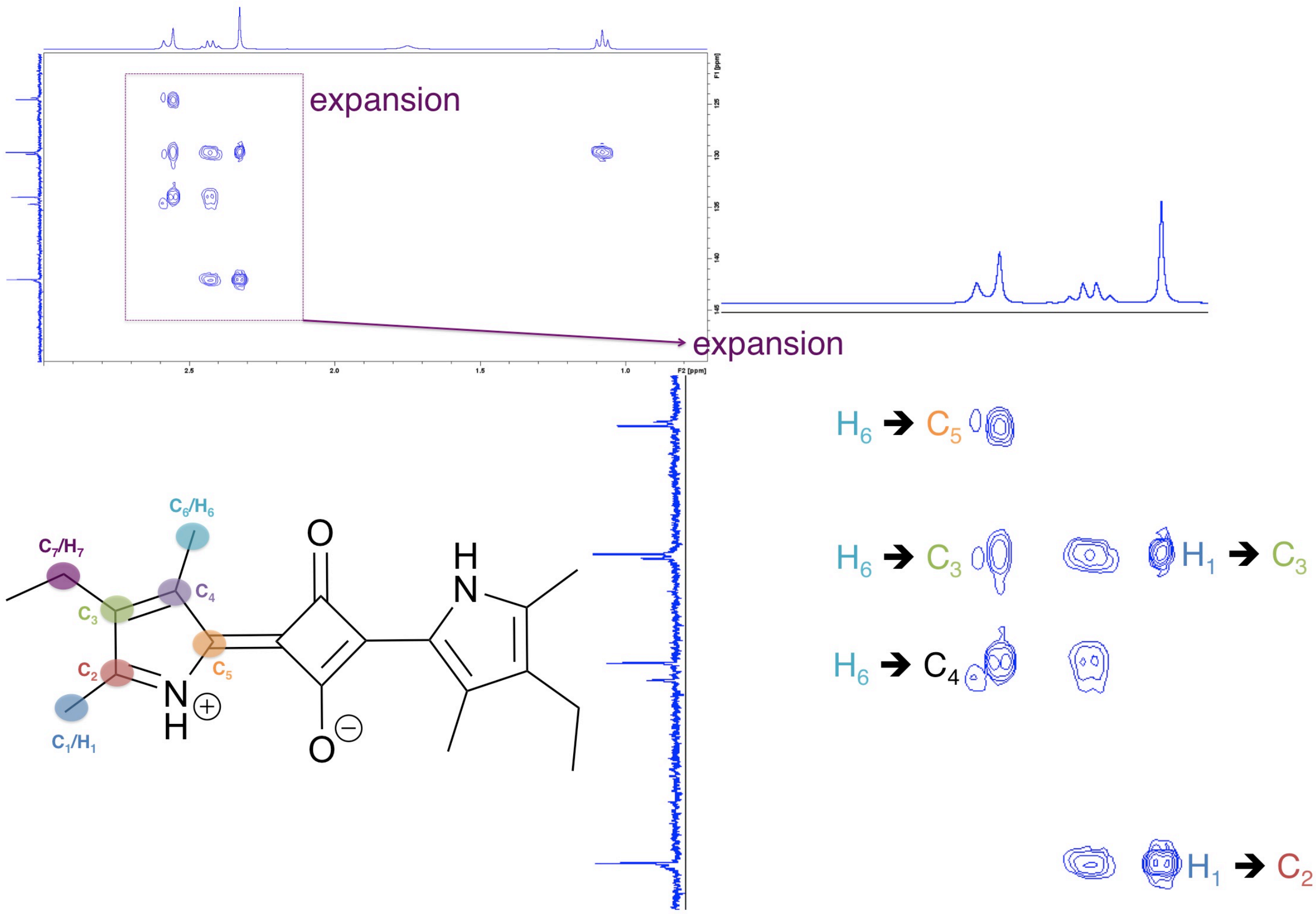


**Figure S3**. Partial HMBC spectrum of **1** with assignment of relevant Hs and Cs.

NMR: 400 MHz, $CDCl_3$.

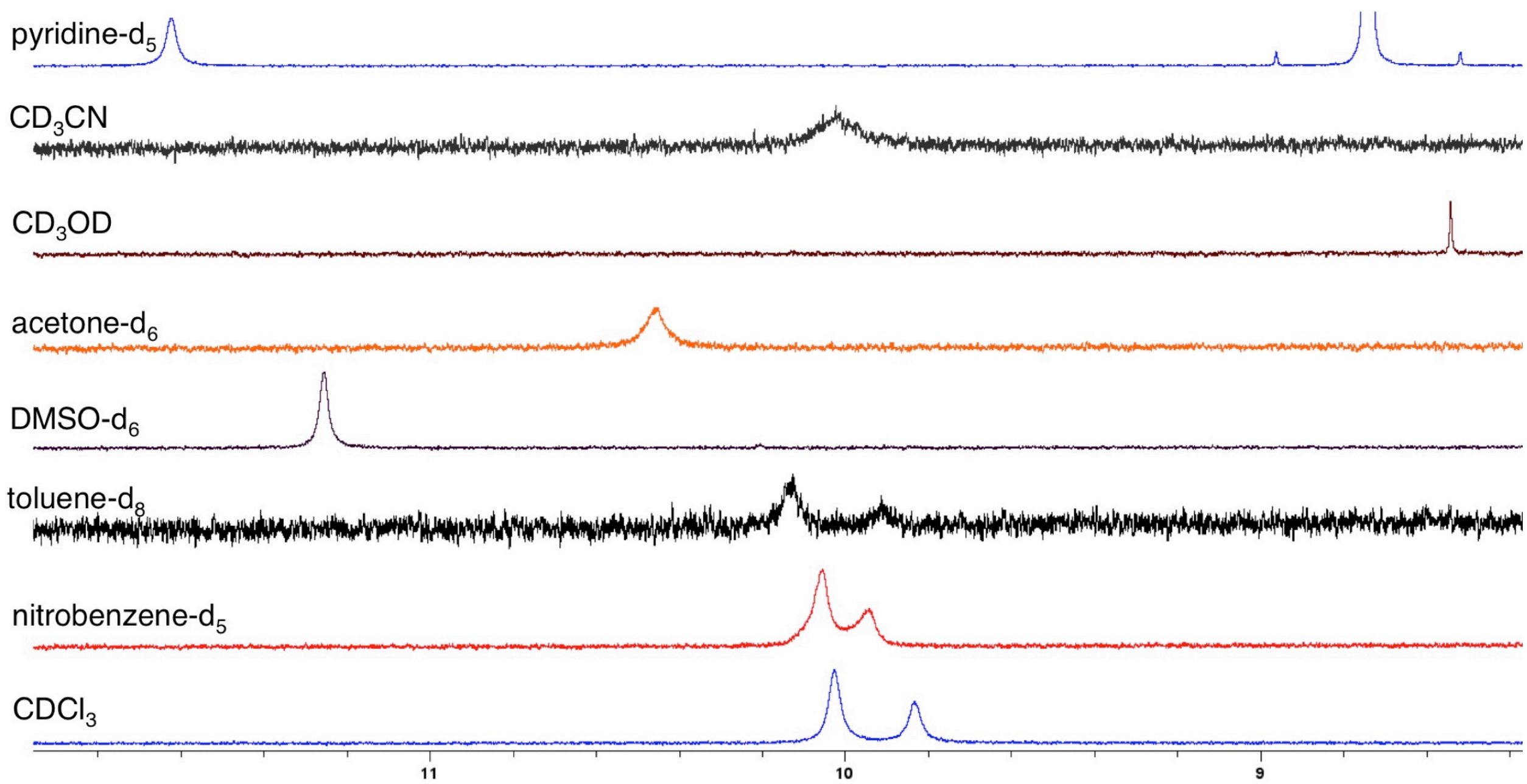


**Figure S4**. Overlaid $^1$H NMR spectra of the NH region of **1** in various deuterated solvents.

| SOLVENT | *anti*-**1** | *syn*-**1** |
|---|---|---|
| $CHCl_3$ | 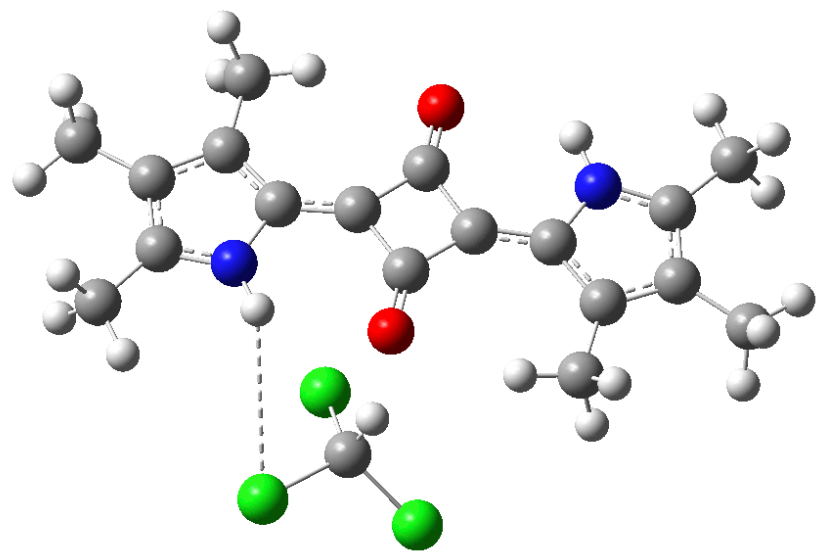 | 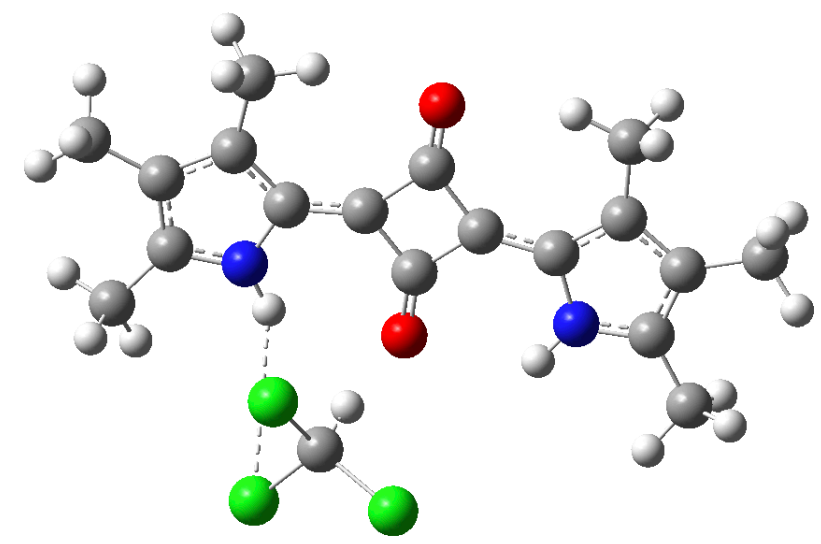 |
| DMSO | 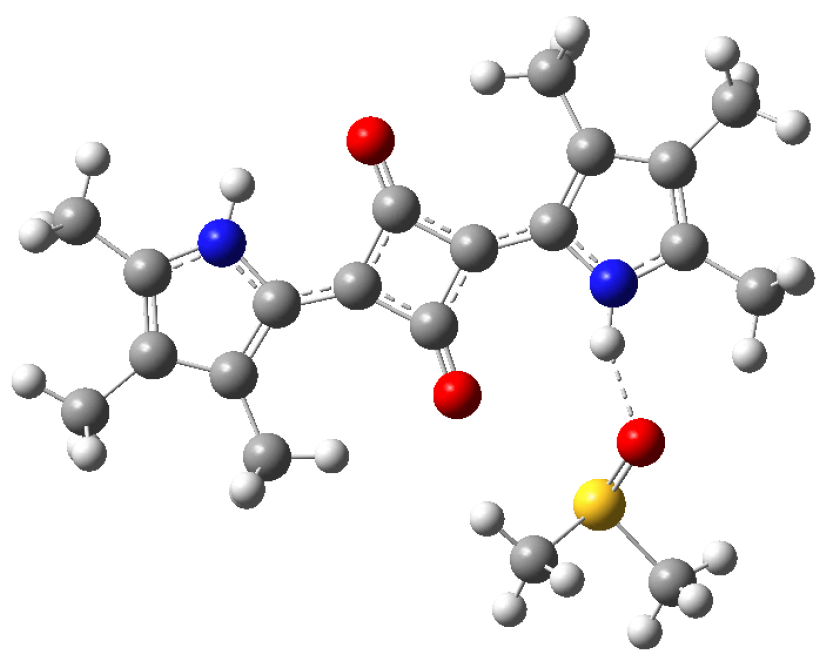 | 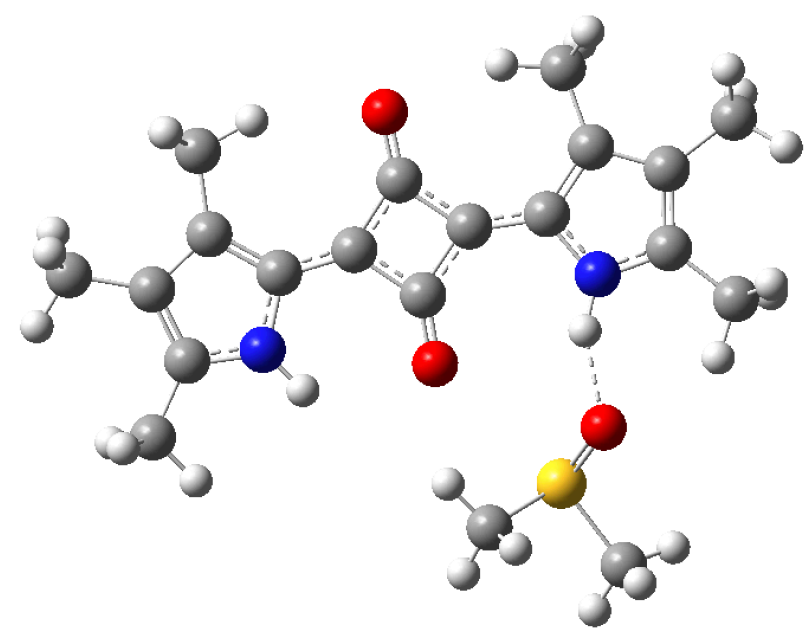 |
| $CH_3OH$ | 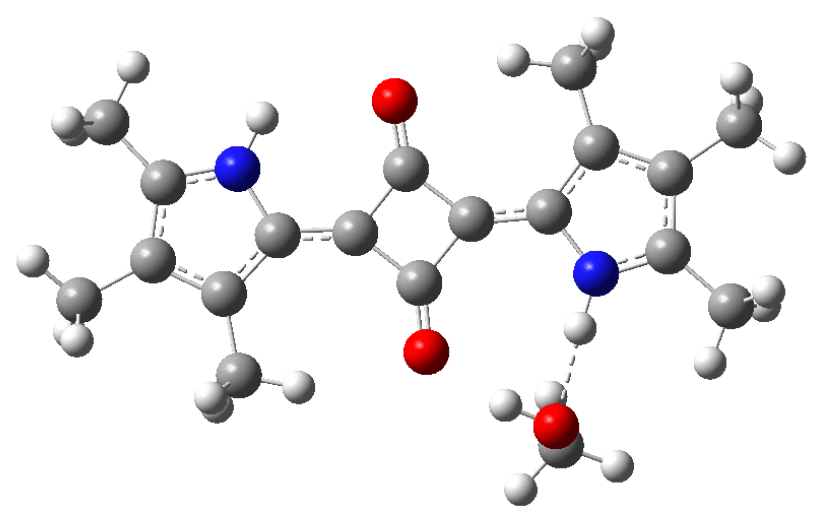 | 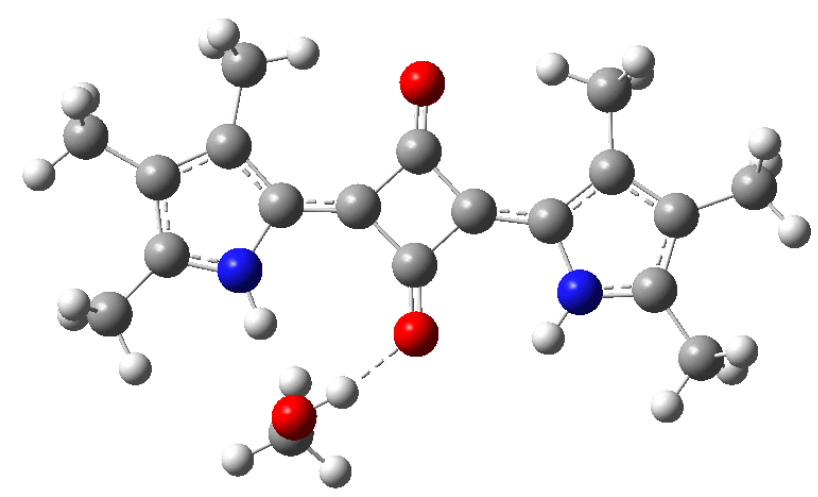 |
| nitrobenzene | 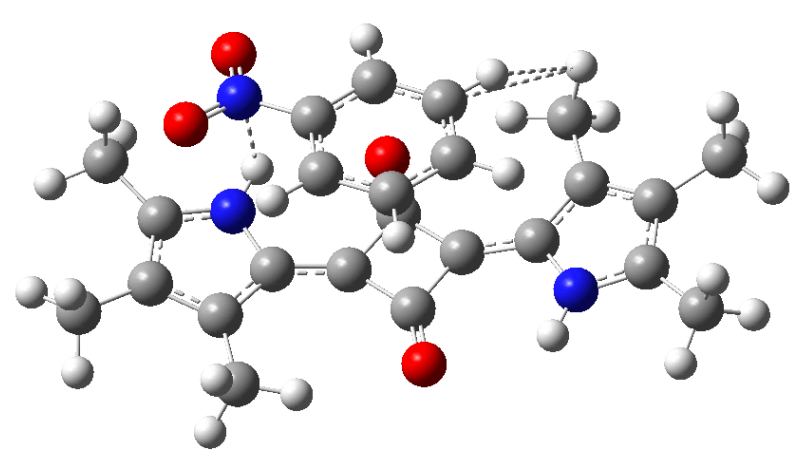 | 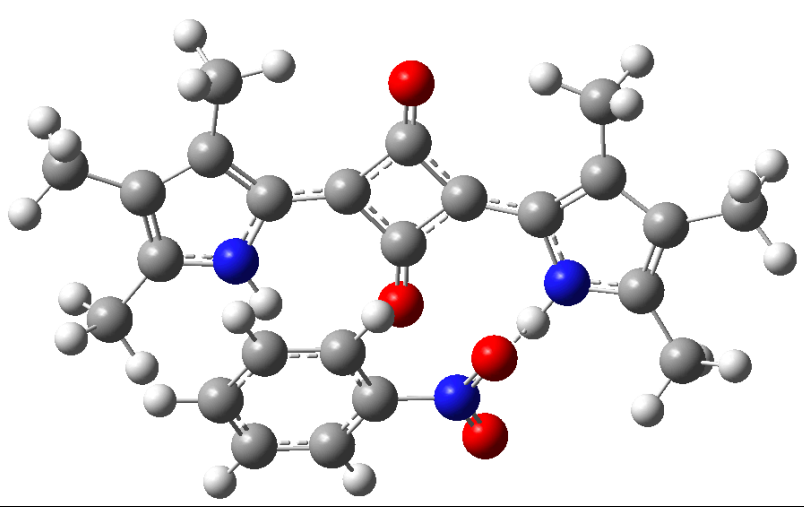 |
| acetone | 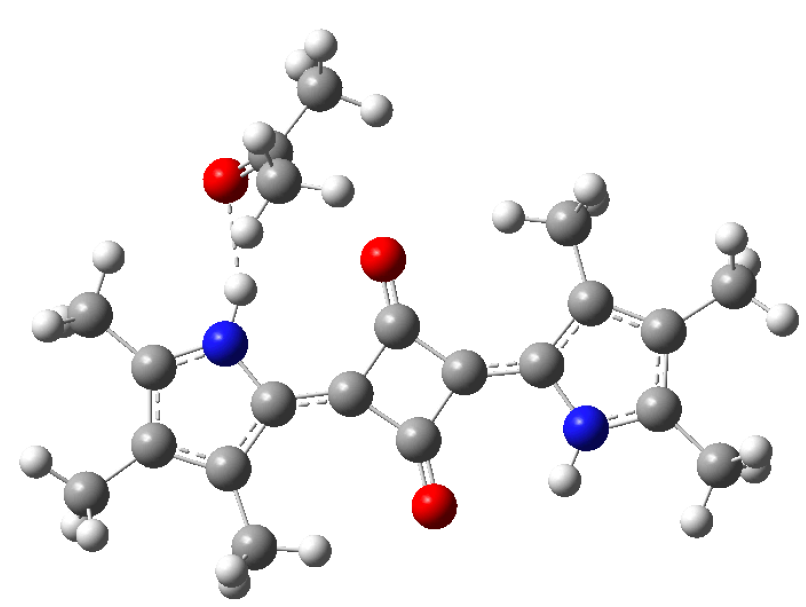 | 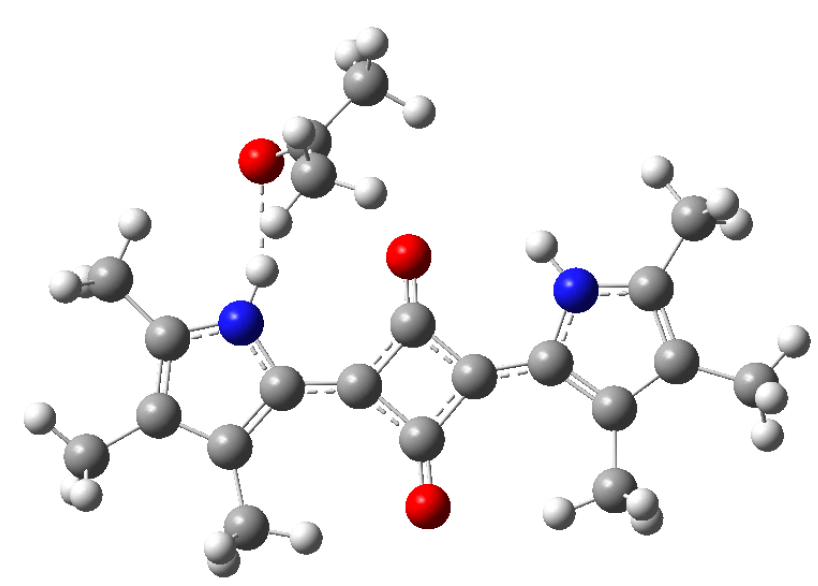 |

**Figure S5**. Computed structures of conformers of dye **1** with coordinated molecule of the solvent.

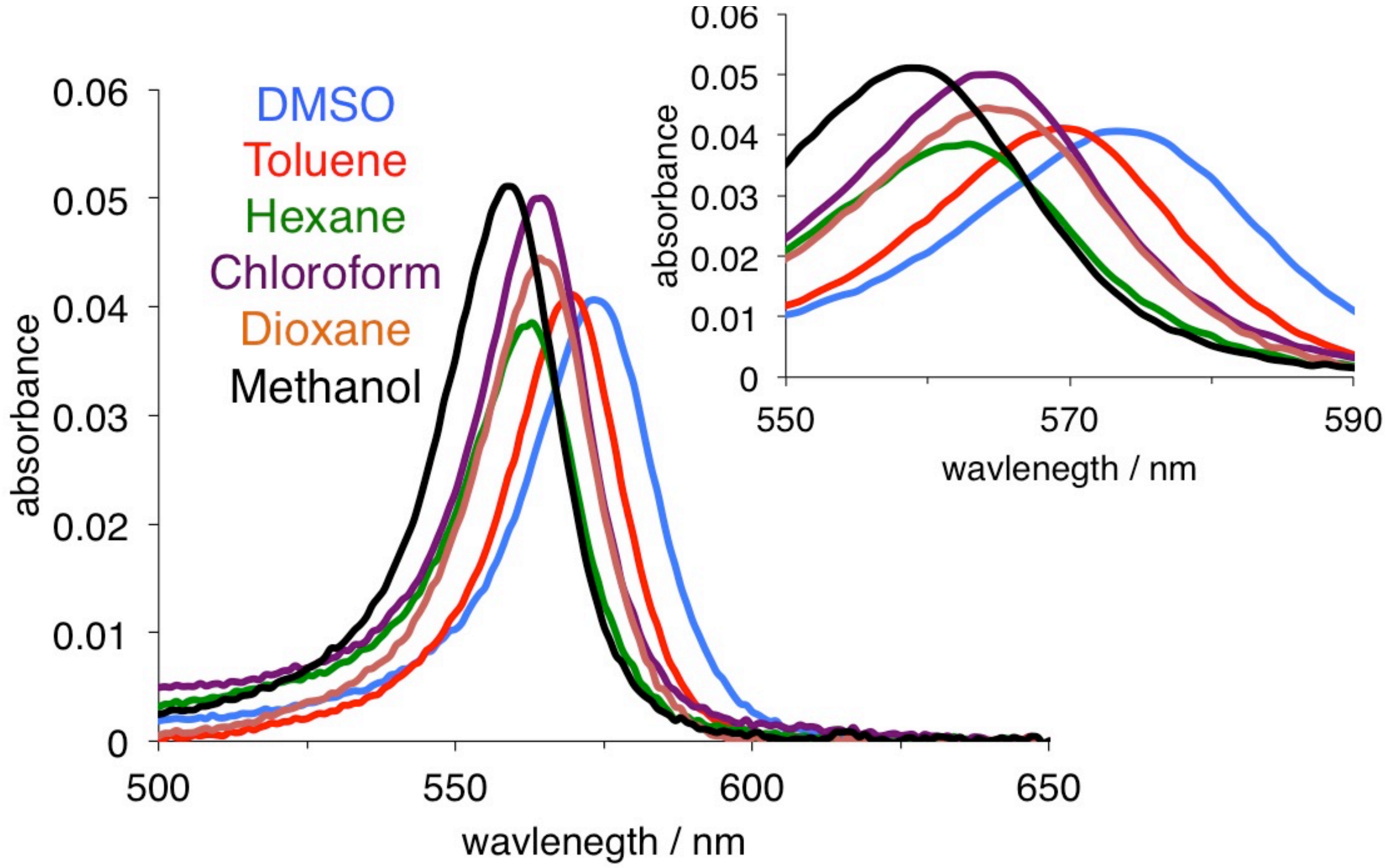


**Figure S6**. Absorbance spectra of **1** in some organic solvents.

Inset: expanded (550 – 590nm) region.
Conditions: [**1**] = 1 μM, dioxane content = 1 % v/v.

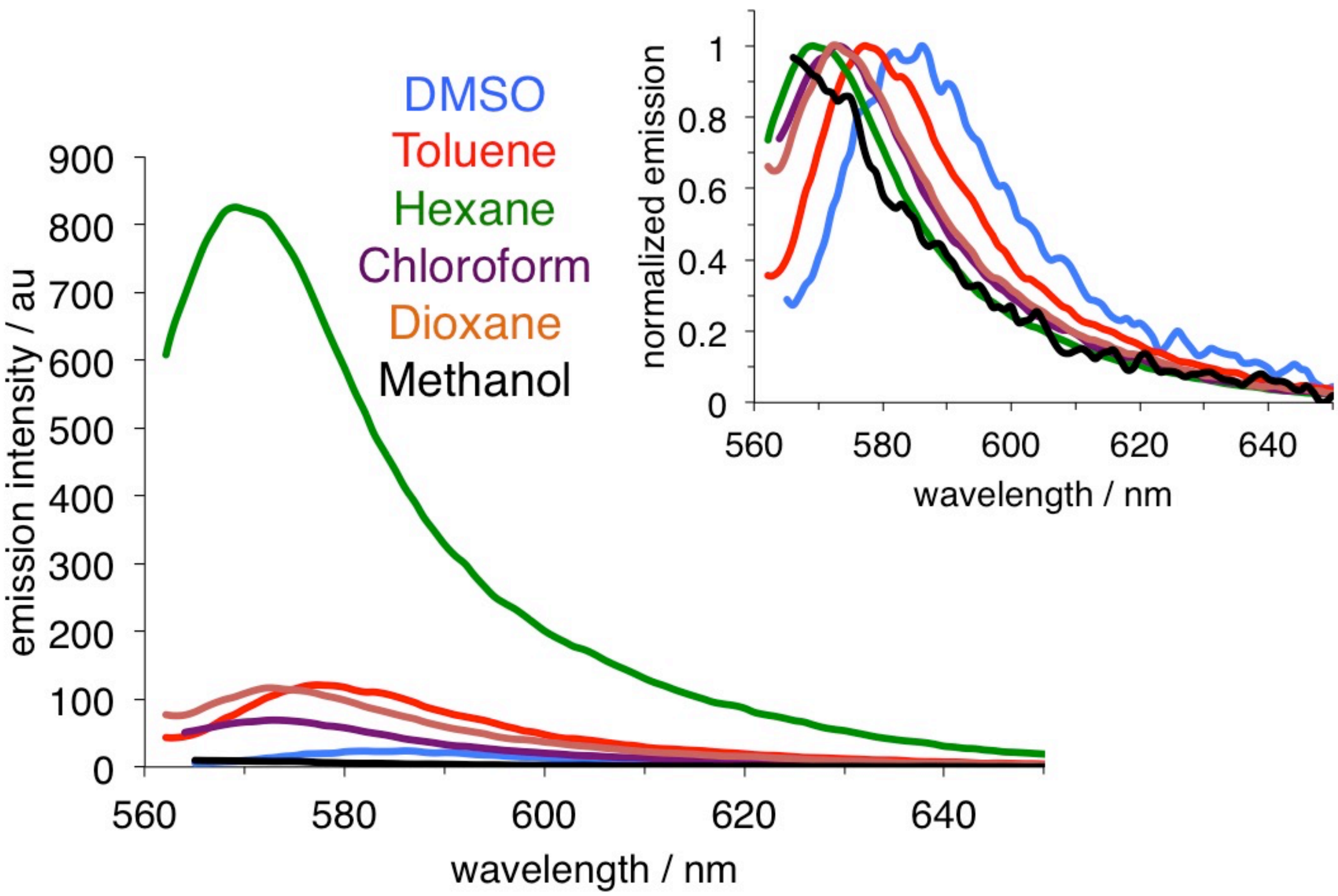


**Figure S7**. Fluorescence emission spectra of **1** in some organic solvents.

Inset: normalized emission spectra.
Conditions: [**1**] = 1 μM, dioxane content = 1 % v/v; λex = 560 nm.

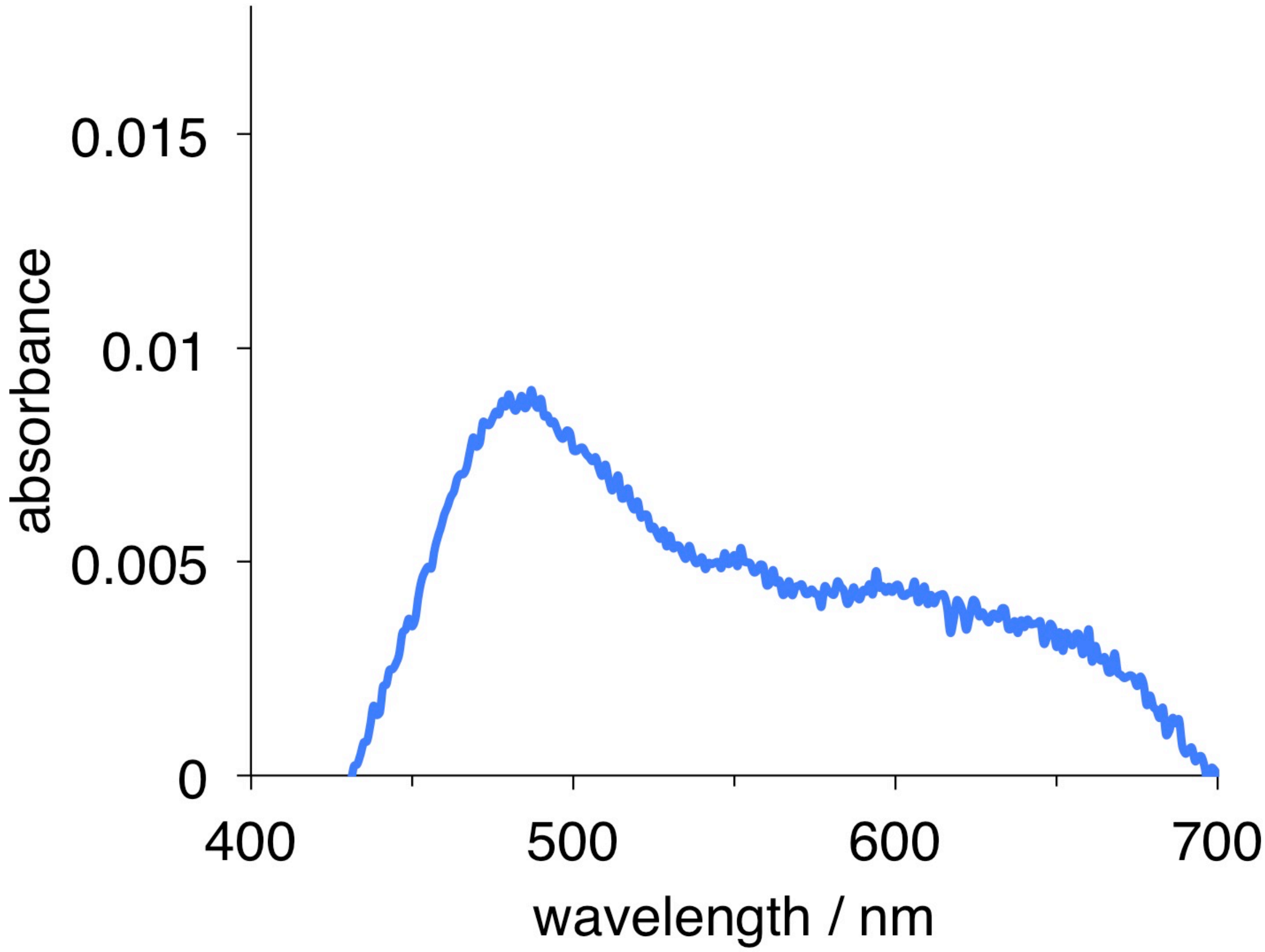


**Figure S8**. Absorbance spectrum of **1** in water.

Conditions: [**1**] = 1 μM, dioxane content = 1 % v/v
**1** was non-emissive in water.

## Dye 1 in DMSO

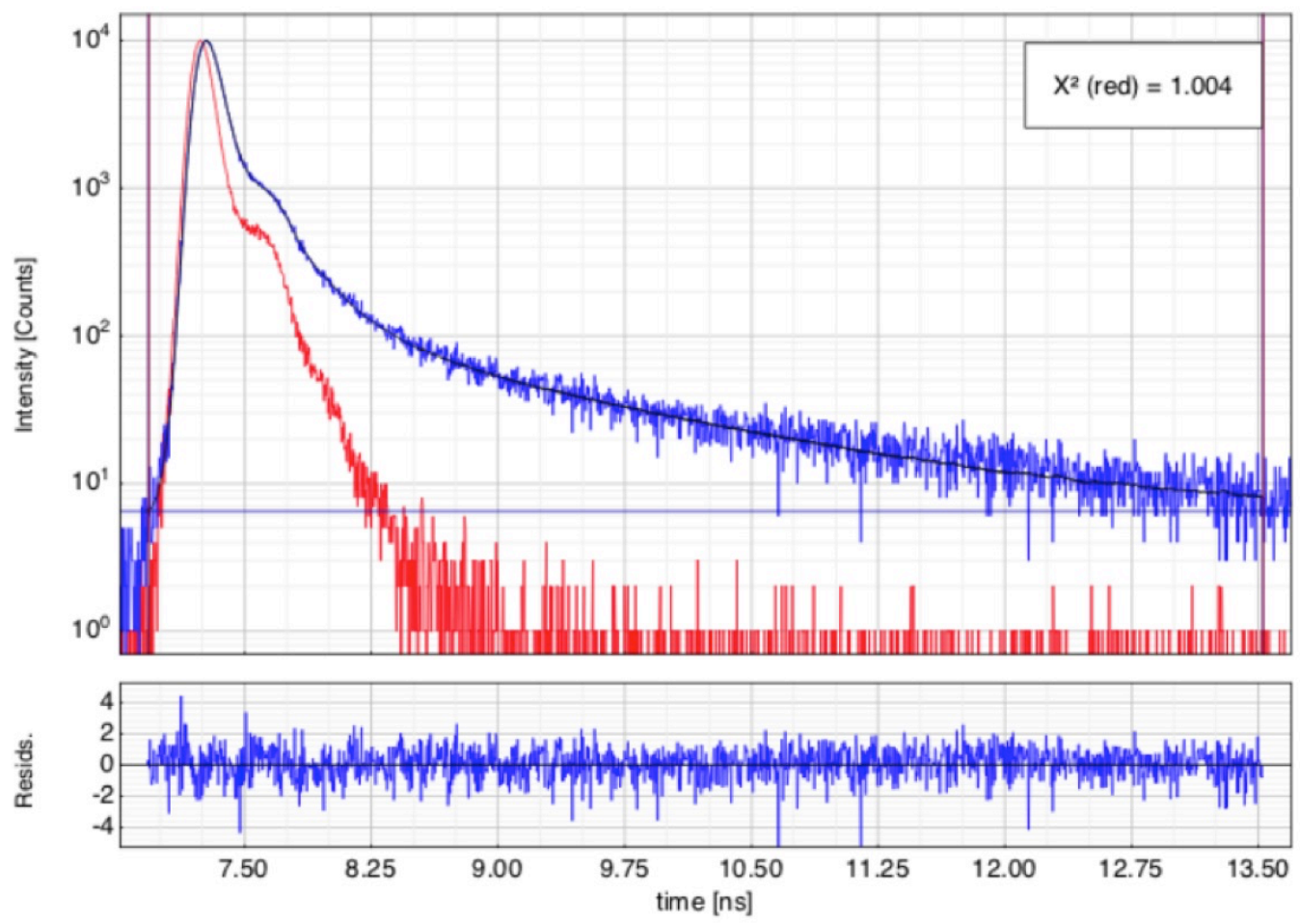


| Parameter | Value | Conf. Lower | Conf. Upper | Conf. Estimation |
|---|---|---|---|---|
| $A_1$ [Cnts] | 135.80 | -5.80 | +5.80 | Fitting |
| $\tau_1$ [ns] | 1.5284 | -0.0465 | +0.0465 | Fitting |
| $A_2$ [Cnts] | 1059.7 | -55.1 | +55.1 | Fitting |
| $\tau_2$ [ns] | 0.27381 | -0.00948 | +0.00948 | Fitting |
| $A_3$ [Cnts] | 51403 | -504 | +504 | Fitting |
| $\tau_3$ [ns] | 0.039563 | -0.000358 | +0.000358 | Fitting |
| Bkgr. Dec [Cnts] | 6.508 | -0.764 | +0.764 | Fitting |
| Bkgr. IRF [Cnts] | 0.639 | -0.550 | +0.550 | Fitting |
| Shift IRF [ns] | 0.041497 | -0.000742 | +0.000742 | Fitting |
| A Scat [Cnts] | -17290 | -4130 | +4130 | Fitting |

Average Lifetime:

$\tau_{Av.1}$=0.1885 ns (intensity weighted)

$\tau_{Av.2}$=0.0481 ns (amplitude weighted)

Fractional Intensities of the Positive Decay Components:

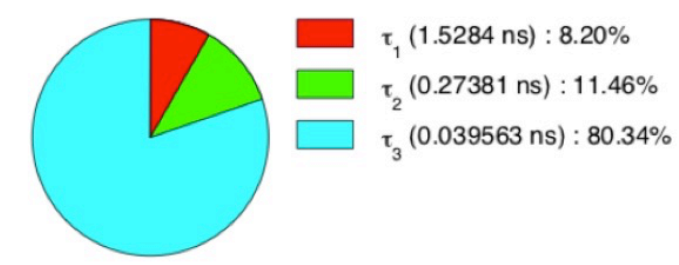


Fractional Amplitudes of the Positive Decay Components:

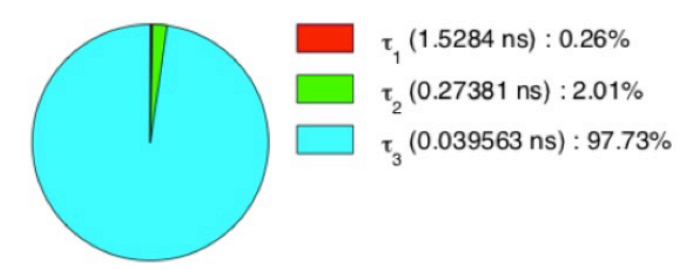


## Dye 1 in toluene

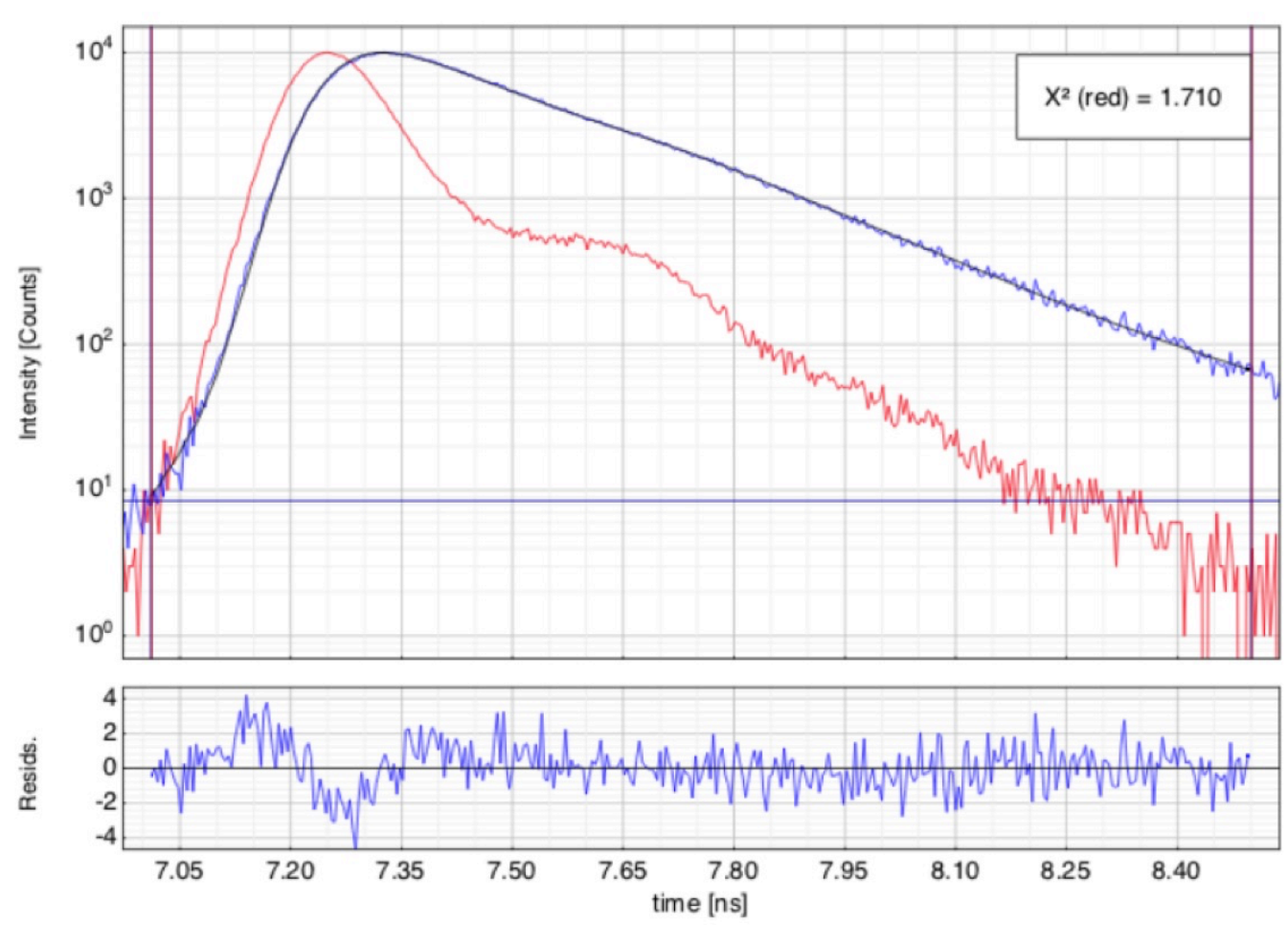


| Parameter | Value | Conf. Lower | Conf. Upper | Conf. Estimation |
|---|---|---|---|---|
| $A_1$ [Cnts] | 21231.9 | -89.4 | +89.4 | Fitting |
| $\tau_1$ [ns] | 0.172590 | -0.000585 | +0.000585 | Fitting |
| Bkgr. Dec [Cnts] | 8.49 | -2.71 | +2.71 | Fitting |
| Bkgr. IRF [Cnts] | -5.43 | -1.89 | +1.89 | Fitting |
| Shift IRF [ns] | 0.053559 | -0.000528 | +0.000528 | Fitting |

Average Lifetime:

$\tau_{Av.1}$=0.172590 ns (intensity weighted)

$\tau_{Av.2}$=0.172590 ns (amplitude weighted)

Fractional Intensities of the Positive Decay Components:

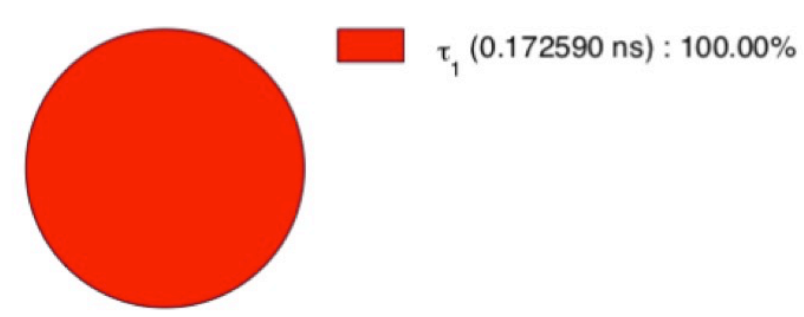


Fractional Amplitudes of the Positive Decay Components:

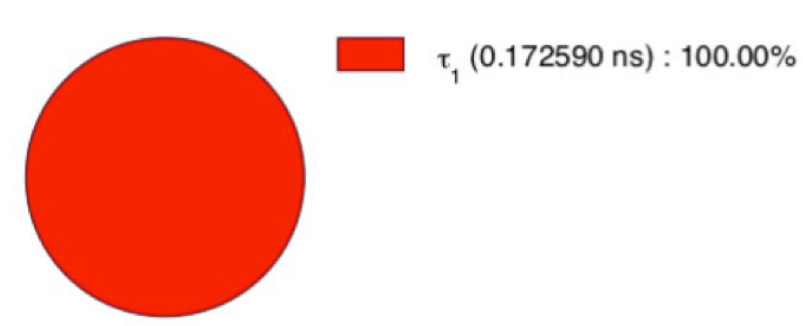


**Figure S9**. Fluorescence intensity decays of **1** in some organic solvents.

## Dye 1 in hexane

| Parameter | Value | Conf. Lower | Conf. Upper | Conf. Estimation |
|---|---|---|---|---|
| $A_1$ [Cnts] | 10088.6 | -52.3 | +52.3 | Fitting |
| $\tau_1$ [ns] | 1.62764 | -0.00582 | +0.00582 | Fitting |
| $A_2$ [Cnts] | 3311 | -135 | +135 | Fitting |
| $\tau_2$ [ns] | 0.4972 | -0.0226 | +0.0226 | Fitting |
| Bkgr. Dec [Cnts] | 0.893 | -0.837 | +0.837 | Fitting |
| Bkgr. IRF [Cnts] | -0.0076 | -0.0983 | +0.0983 | Fitting |
| Shift IRF [ns] | 0.00971 | -0.00145 | +0.00145 | Fitting |
| A Scat [Cnts] | 61080 | -5880 | +5880 | Fitting |

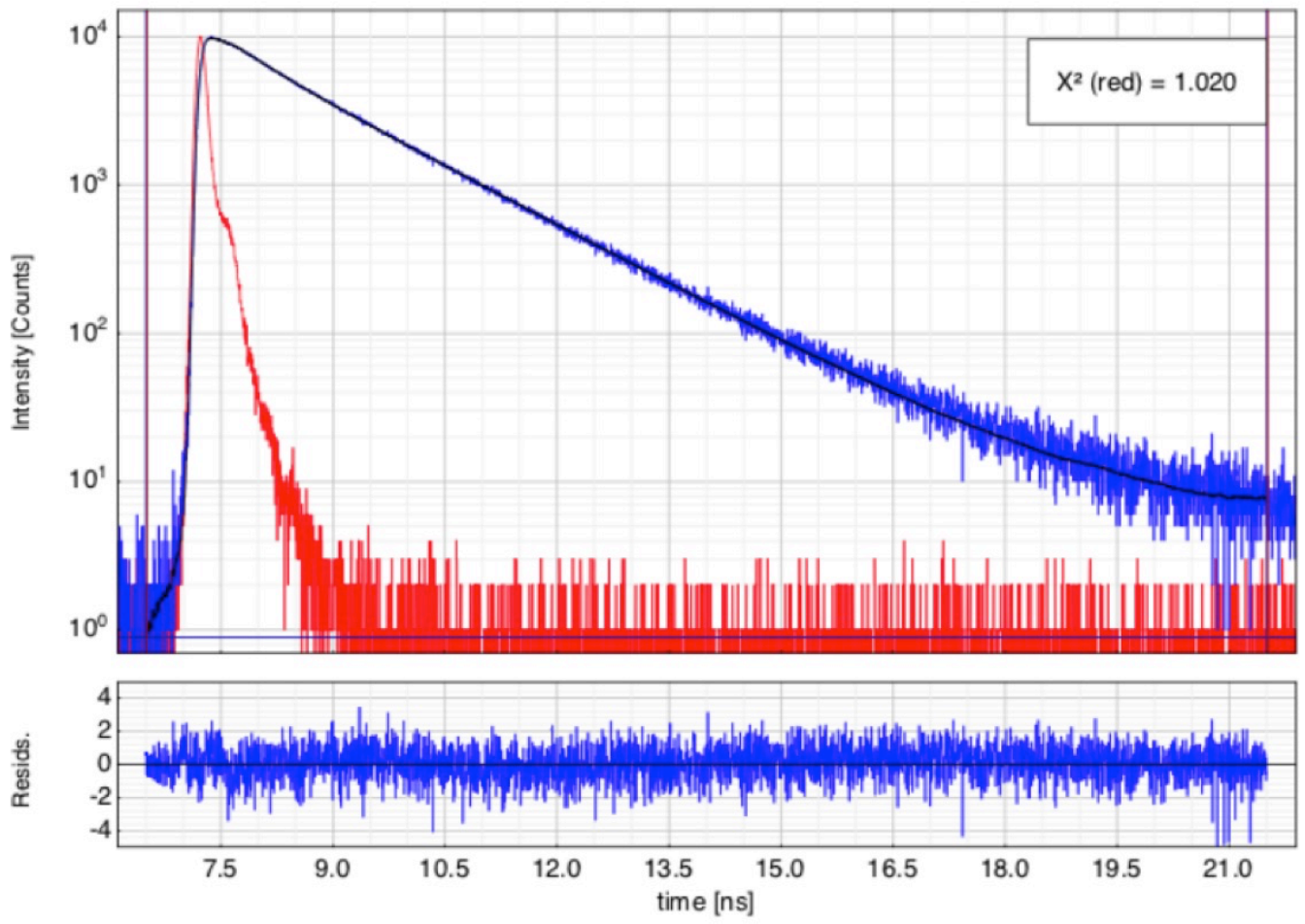


Average Lifetime:

$\tau_{Av.1}$=1.52464 ns (intensity weighted)

$\tau_{Av.2}$=1.34834 ns (amplitude weighted)

Fractional Intensities of the Positive Decay Components:

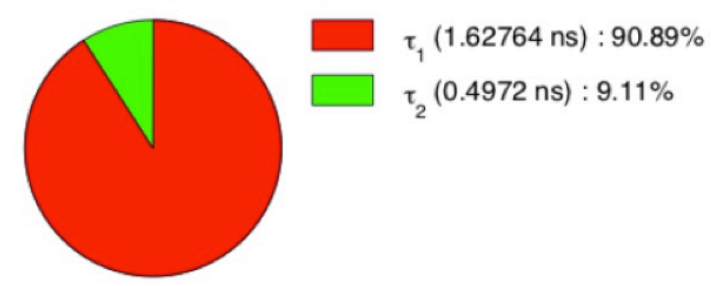


Fractional Amplitudes of the Positive Decay Components:

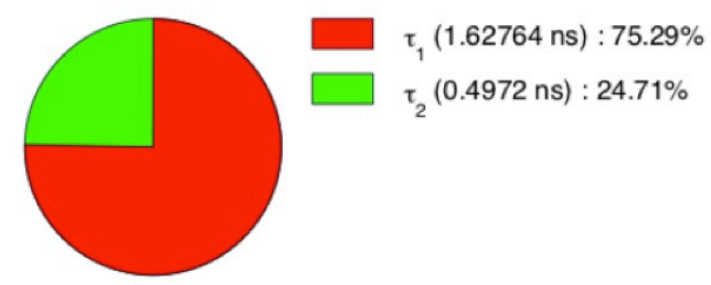


## Dye 1 in chloroform

| Parameter | Value | Conf. Lower | Conf. Upper | Conf. Estimation |
|---|---|---|---|---|
| $A_1$ [Cnts] | 266.9 | -11.7 | +11.7 | Fitting |
| $\tau_1$ [ns] | 0.6065 | -0.0214 | +0.0214 | Fitting |
| $A_2$ [Cnts] | 26037 | -105 | +105 | Fitting |
| $\tau_2$ [ns] | 0.106958 | -0.000369 | +0.000369 | Fitting |
| Bkgr. Dec [Cnts] | 2.57 | -2.12 | +2.12 | Fitting |
| Bkgr. IRF [Cnts] | -11.89 | -1.60 | +1.60 | Fitting |
| Shift IRF [ns] | 0.045425 | -0.000412 | +0.000412 | Fitting |

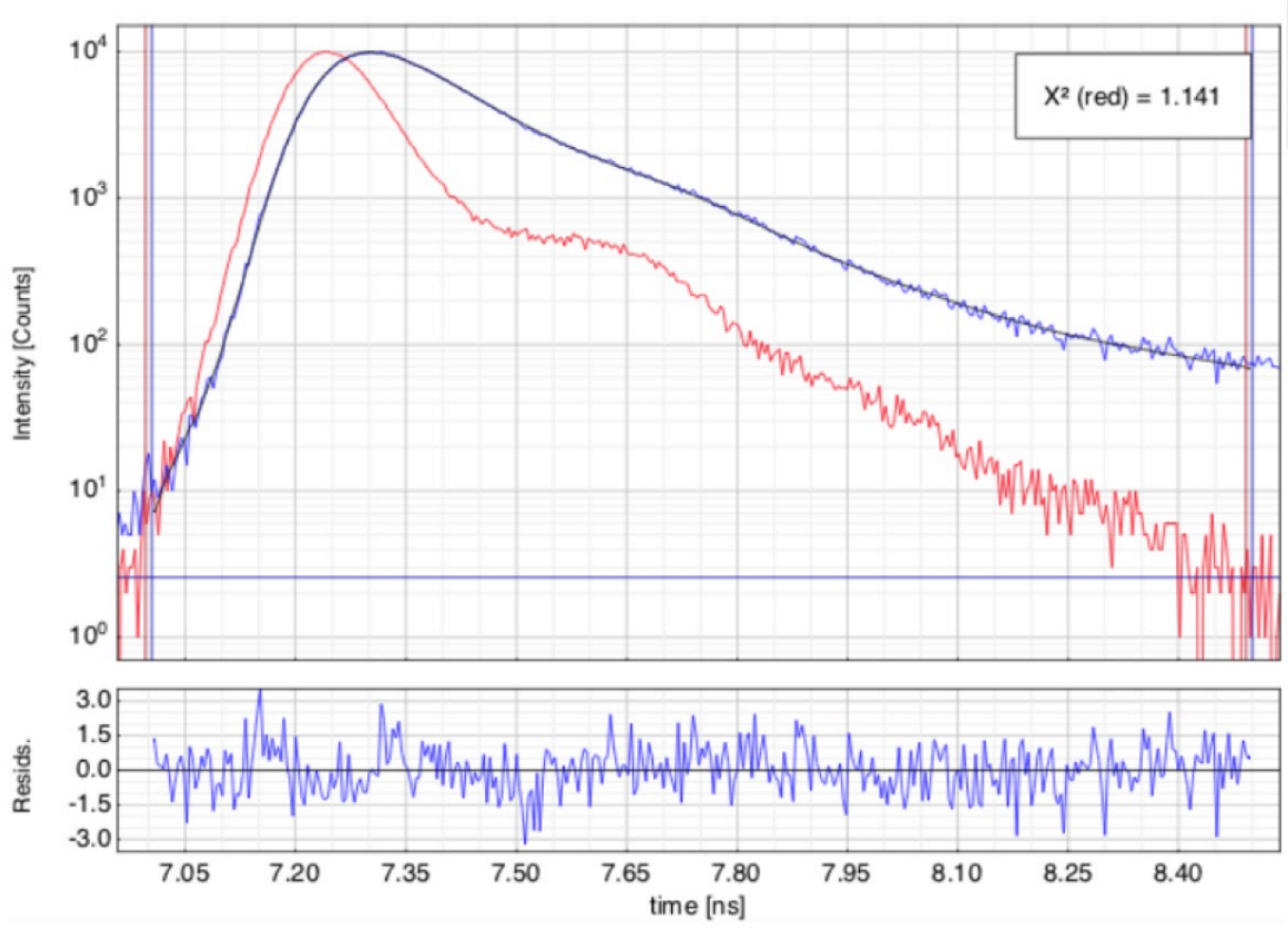


Average Lifetime:

$\tau_{Av.1}$=0.1344 ns (intensity weighted)

$\tau_{Av.2}$=0.1120 ns (amplitude weighted)

Fractional Intensities of the Positive Decay Components:

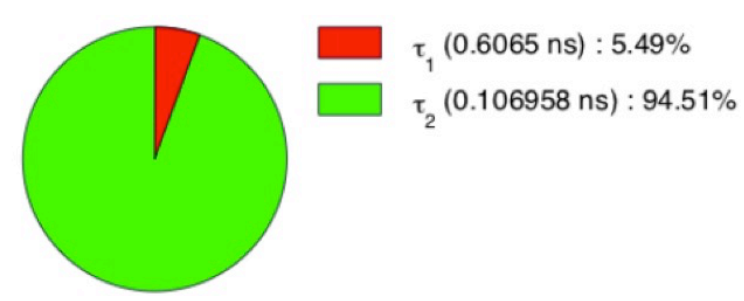


Fractional Amplitudes of the Positive Decay Components:

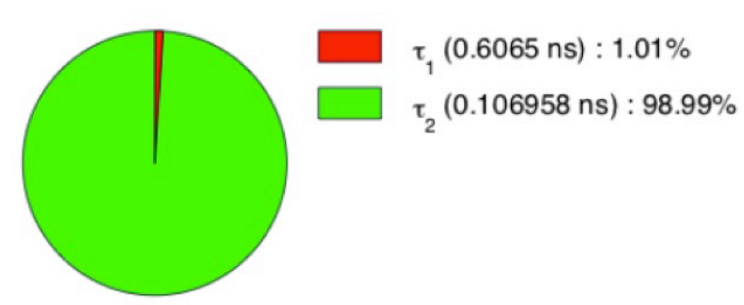


**Figure S9 (cont'd)**. Fluorescence intensity decays of **1** in some organic solvents.

**Dye 1 in dioxane**

| Parameter | Value | Conf. Lower | Conf. Upper | Conf. Estimation |
|---|---|---|---|---|
| $A_1$ [Cnts] | 338.8 | -10.3 | +10.3 | Fitting |
| $\tau_1$ [ns] | 1.2812 | -0.0238 | +0.0238 | Fitting |
| $A_2$ [Cnts] | 7955 | -267 | +267 | Fitting |
| $\tau_2$ [ns] | 0.09579 | -0.00316 | +0.00316 | Fitting |
| $A_3$ [Cnts] | 16140 | -172 | +172 | Fitting |
| $\tau_3$ [ns] | 0.15359 | -0.00135 | +0.00135 | Fitting |
| Bkgr. Dec [Cnts] | -0.387 | -0.544 | +0.544 | Fitting |
| Bkgr. IRF [Cnts] | -1.527 | -0.283 | +0.283 | Fitting |
| Shift IRF [ns] | -0.014155 | -0.000915 | +0.000915 | Fitting |
| A Scat [Cnts] | -12420 | -4090 | +4090 | Fitting |

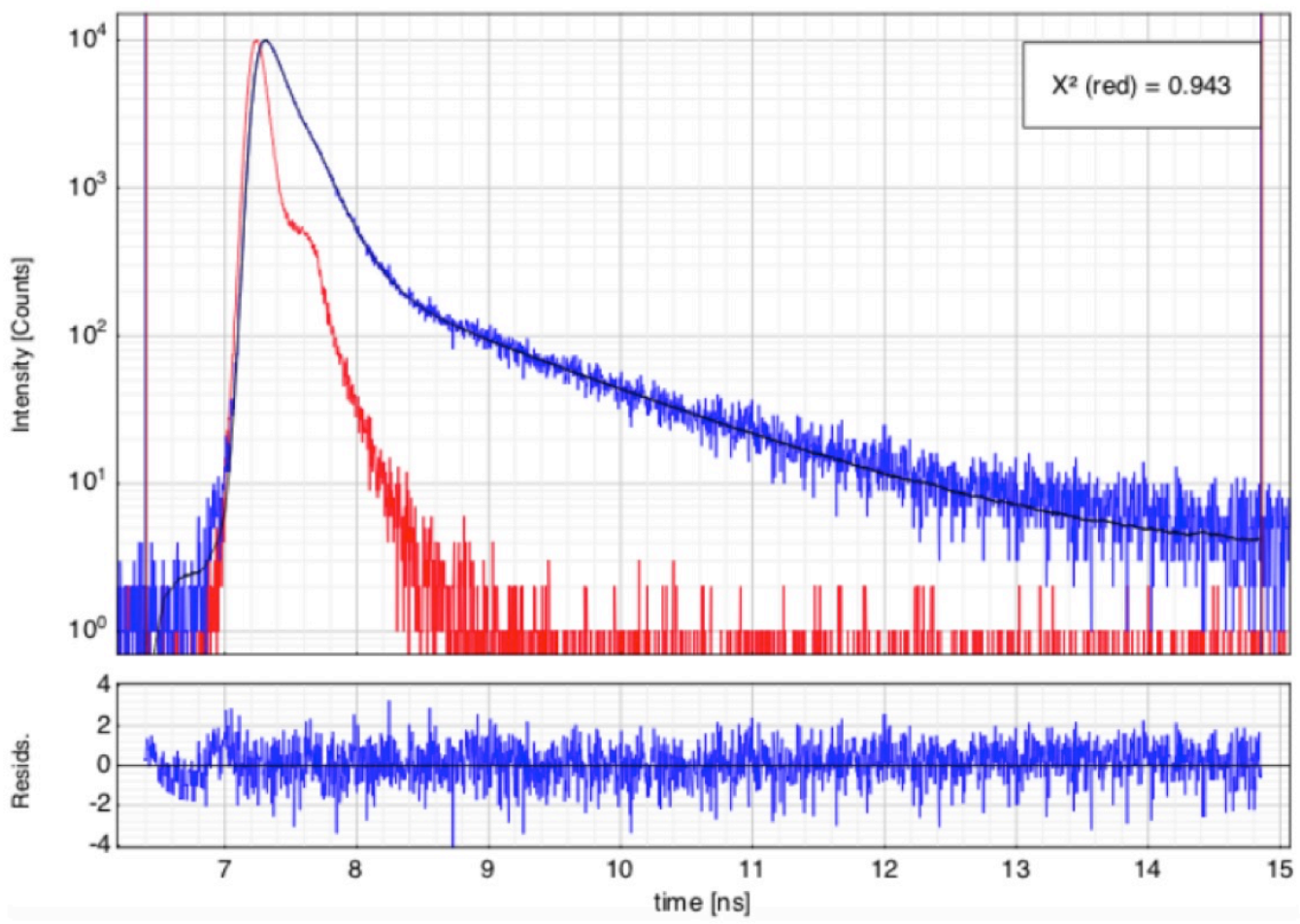


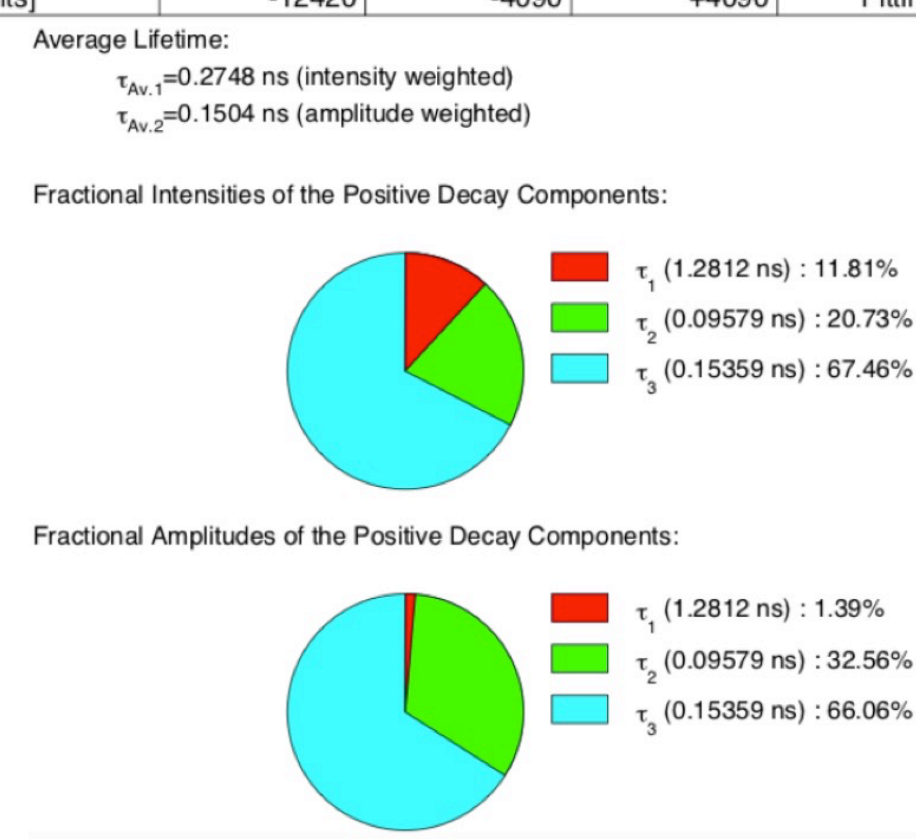


**Dye 1 in methanol**

| Parameter | Value | Conf. Lower | Conf. Upper | Conf. Estimation |
|---|---|---|---|---|
| $A_1$ [Cnts] | 382.8 | -13.2 | +13.2 | Fitting |
| $\tau_1$ [ns] | 0.4257 | -0.0175 | +0.0175 | Fitting |
| $A_2$ [Cnts] | 9296 | -283 | +283 | Fitting |
| $\tau_2$ [ns] | 0.027446 | -0.000710 | +0.000710 | Fitting |
| Bkgr. Dec [Cnts] | -18.97 | -2.82 | +2.82 | Fitting |
| Bkgr. IRF [Cnts] | -28.02 | -2.81 | +2.81 | Fitting |
| Shift IRF [ns] | -0.006445 | -0.000280 | +0.000280 | Fitting |
| A Scat [Cnts] | 371230 | -1840 | +1840 | Fitting |

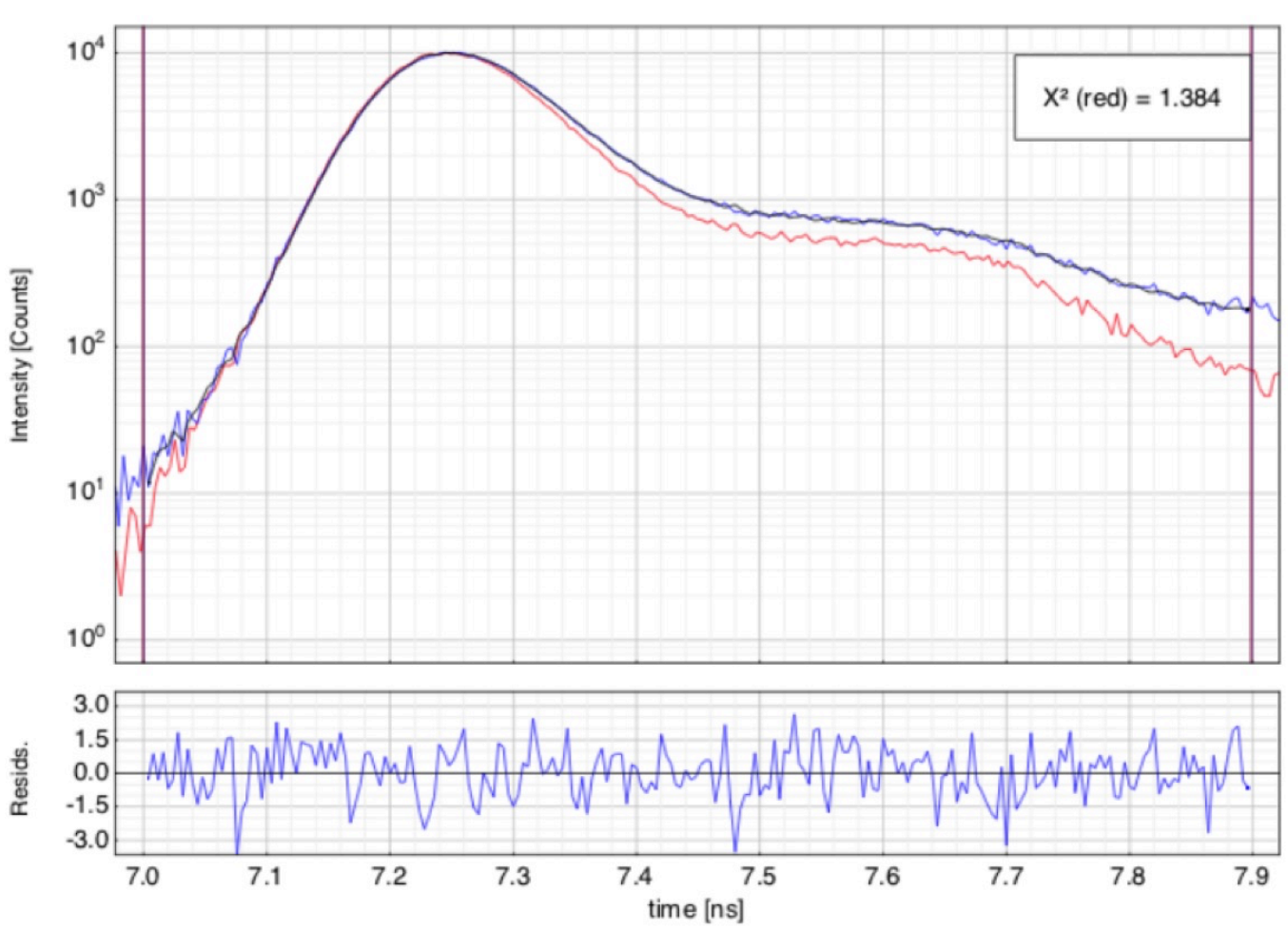


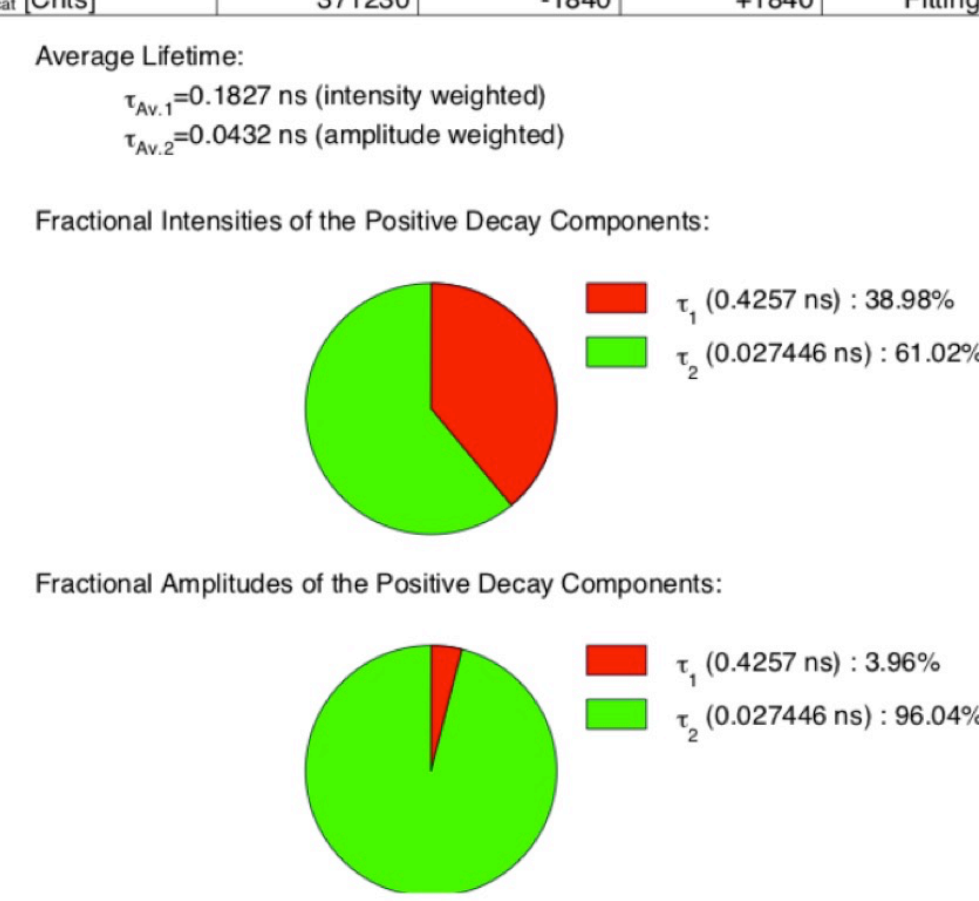


**Figure S9 (cont'd)**. Fluorescence intensity decays of **1** in some organic solvents.

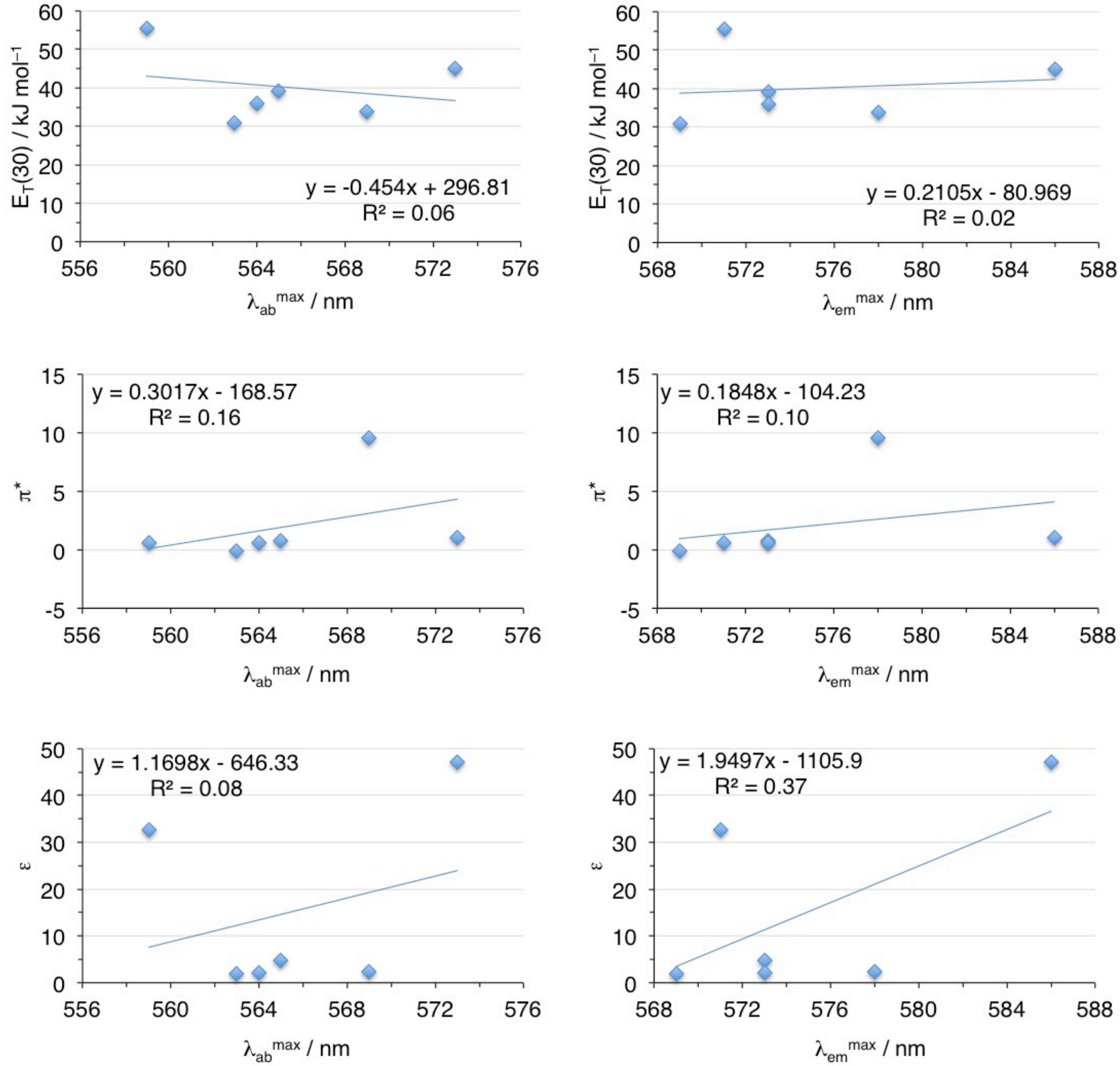


**Figure S10**. Absorption and emission maxima of **1** as a function of solvents' parameters.

Absorption data – left column; emission data – right column.
For absorption maxima ($\lambda_{ab}^{max}$) and emission maxima ($\lambda_{em}^{max}$) values see Table S3.
For solvents' parameter ($E_T(30)$, $\pi^*$ and $\varepsilon$) values see Table S4.

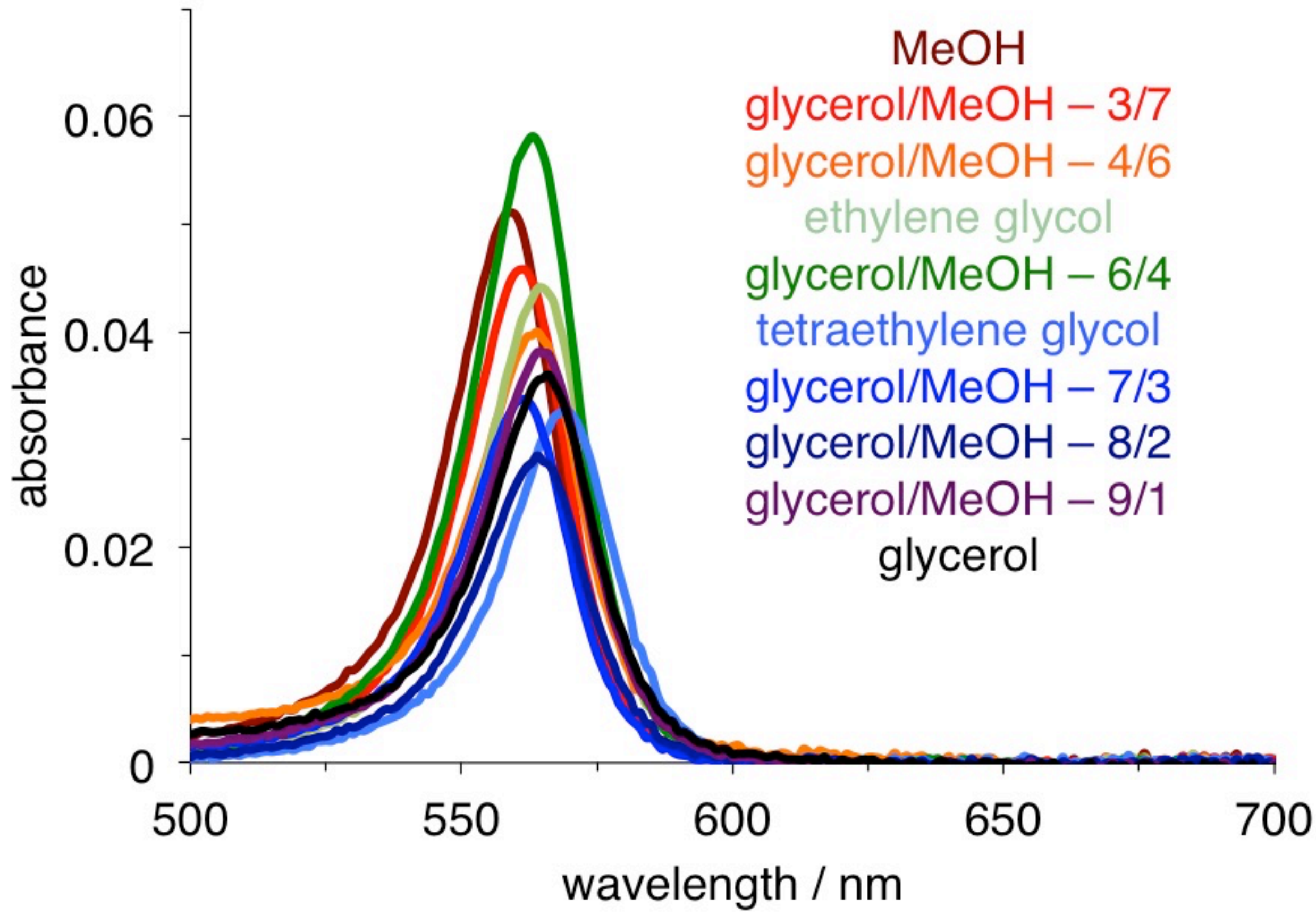


**Figure S11.** Absorbance spectra of **1** in solvents of various viscosities. See Table S5 for solvent viscosity values.

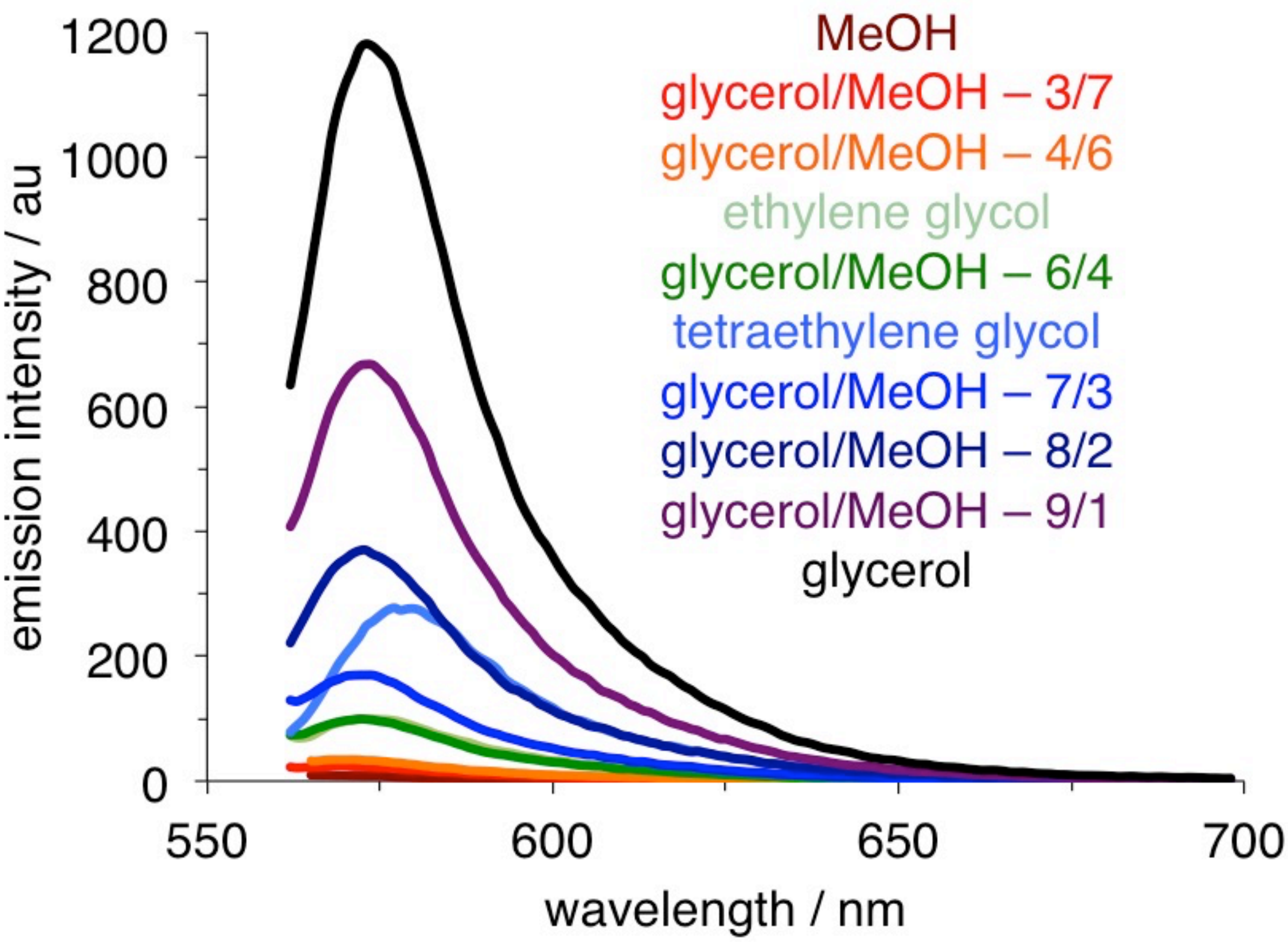


**Figure S12.** Fluorescence emission spectra of **1** in solvents of various viscosities. See Table S5 for solvent viscosity values.

**Dye 1 in MeOH (see figure S9)**

**Dye 1 in glycerol/MeOH (3/7) mixture**

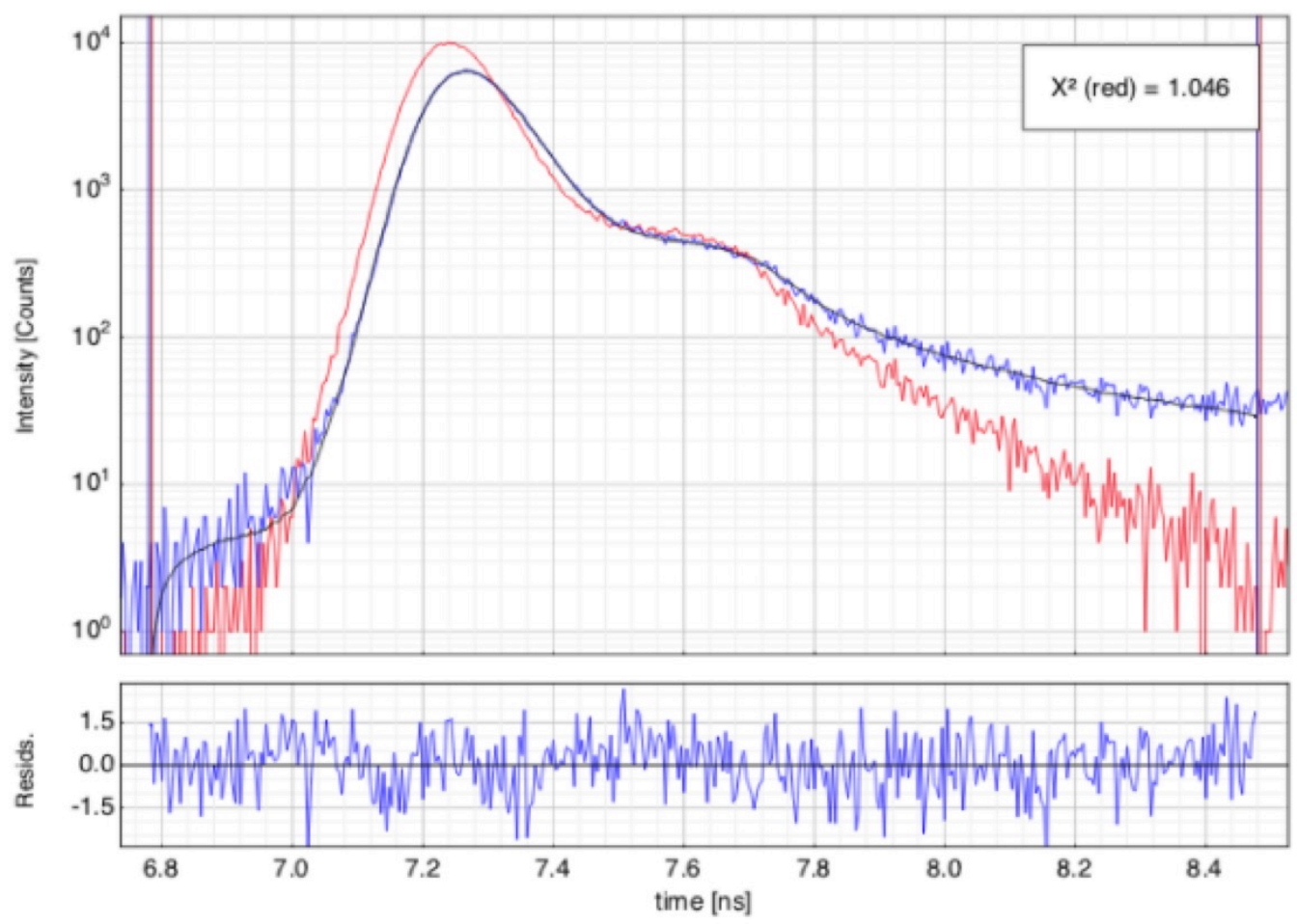


| Parameter | Value | Conf. Lower | Conf. Upper | Conf. Estimation |
|---|---|---|---|---|
| $A_1$ [Cnts] | 41490 | -255 | +255 | Fitting |
| $\tau_1$ [ns] | 0.031366 | -0.000173 | +0.000173 | Fitting |
| $A_2$ [Cnts] | 103.89 | -5.05 | +5.05 | Fitting |
| $\tau_2$ [ns] | 0.7993 | -0.0409 | +0.0409 | Fitting |
| Bkgr. $_{Dec}$ [Cnts] | 0.555 | -0.835 | +0.835 | Fitting |
| Bkgr. $_{IRF}$ [Cnts] | -4.10 | -1.29 | +1.29 | Fitting |
| Shift $_{IRF}$ [ns] | -0.012281 | -0.000447 | +0.000447 | Fitting |

Average Lifetime:

$\tau_{Av.1}$=0.077424 ns (intensity weighted)

$\tau_{Av.2}$=0.033284 ns (amplitude weighted)

Fractional Intensities of the Positive Decay Components:

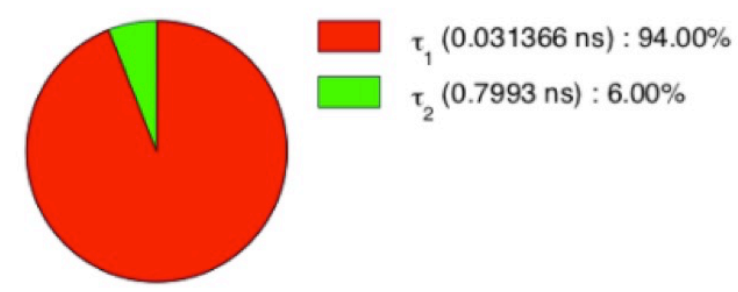


Fractional Amplitudes of the Positive Decay Components:

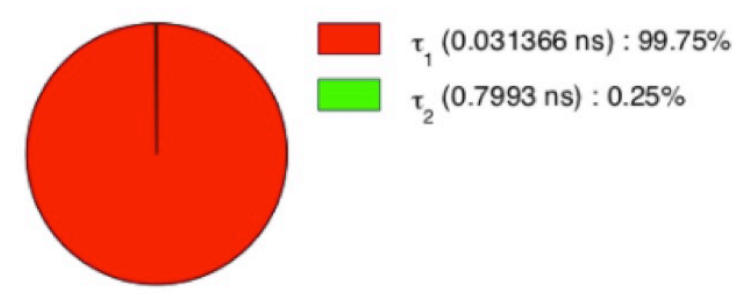


**Dye 1 in glycerol/MeOH (4/6) mixture**

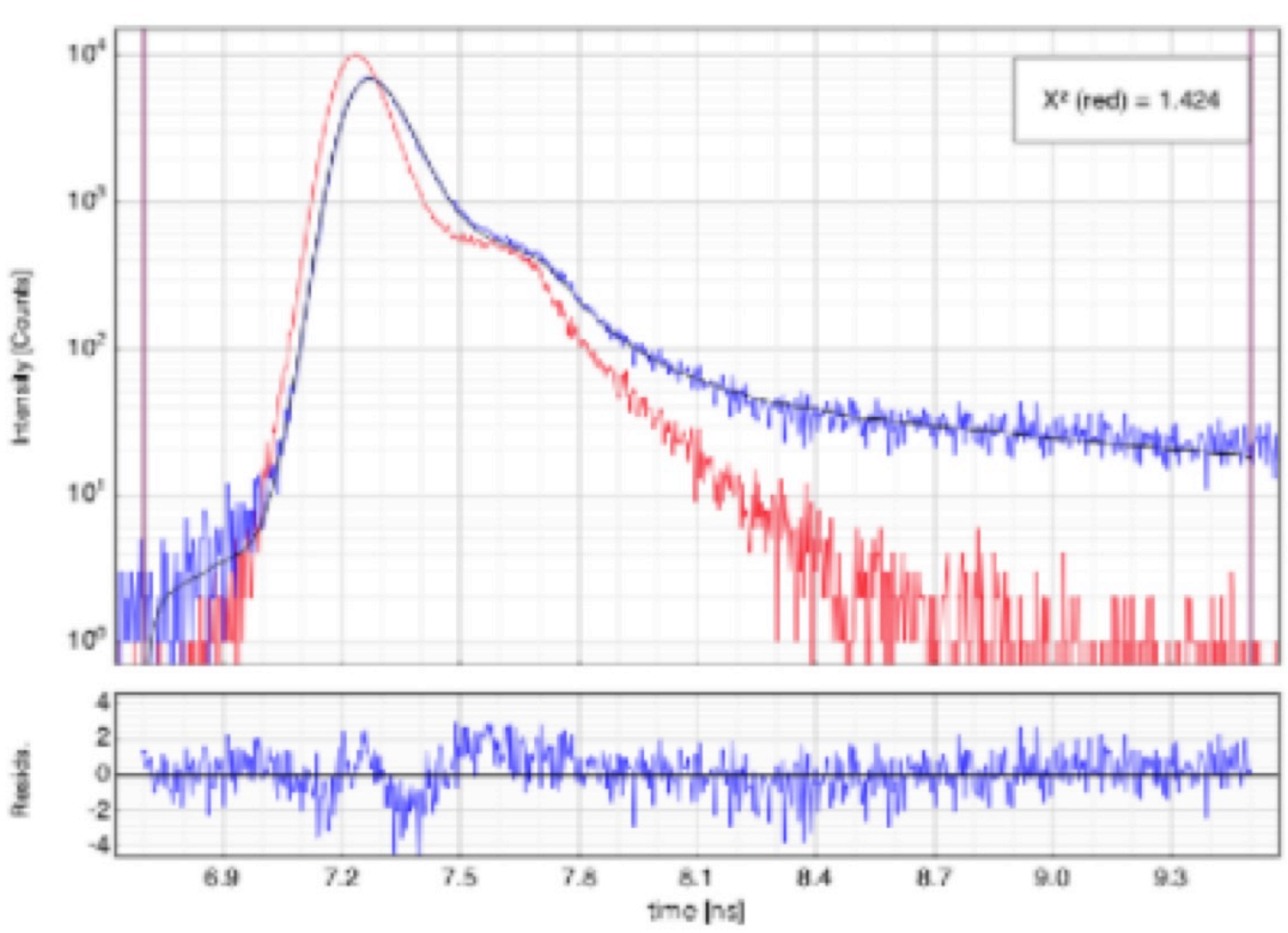


| Parameter | Value | Conf. Lower | Conf. Upper | Conf. Estimation |
|---|---|---|---|---|
| $A_1$ [Cnts] | 31275 | -264 | +264 | Fitting |
| $\tau_1$ [ns] | 0.048126 | -0.000360 | +0.000360 | Fitting |
| $A_2$ [Cnts] | 64.30 | -3.49 | +3.49 | Fitting |
| $\tau_2$ [ns] | 1.5462 | -0.0982 | +0.0982 | Fitting |
| Bkgr. $_{Dec}$ [Cnts] | 0.277 | -0.867 | +0.867 | Fitting |
| Bkgr. $_{IRF}$ [Cnts] | -2.90 | -1.11 | +1.11 | Fitting |
| Shift $_{IRF}$ [ns] | -0.017376 | -0.000683 | +0.000683 | Fitting |

Average Lifetime:

$\tau_{Av.1}$=0.140947 ns (intensity weighted)

$\tau_{Av.2}$=0.051200 ns (amplitude weighted)

Fractional Intensities of the Positive Decay Components:

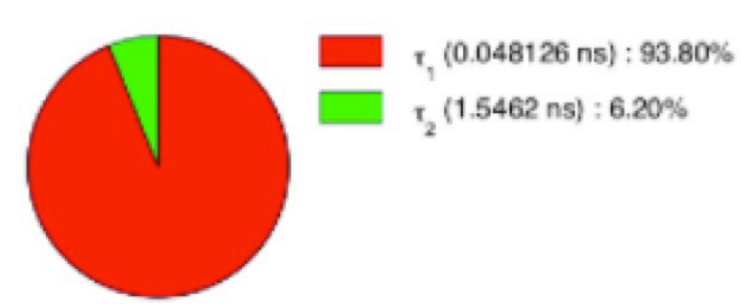


Fractional Amplitudes of the Positive Decay Components:

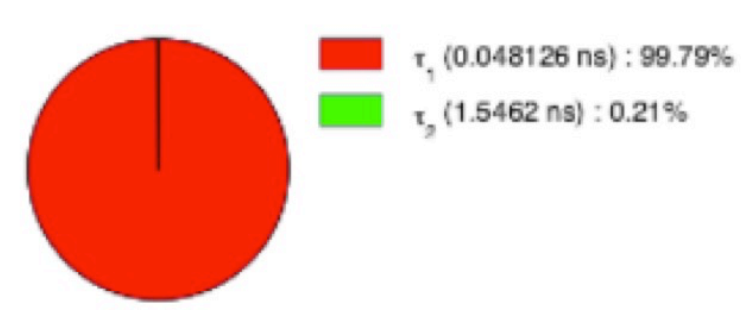


**Figure S13**. Fluorescence intensity decays of **1** in solvents of various viscosities.

## Dye 1 in ethylene glycol

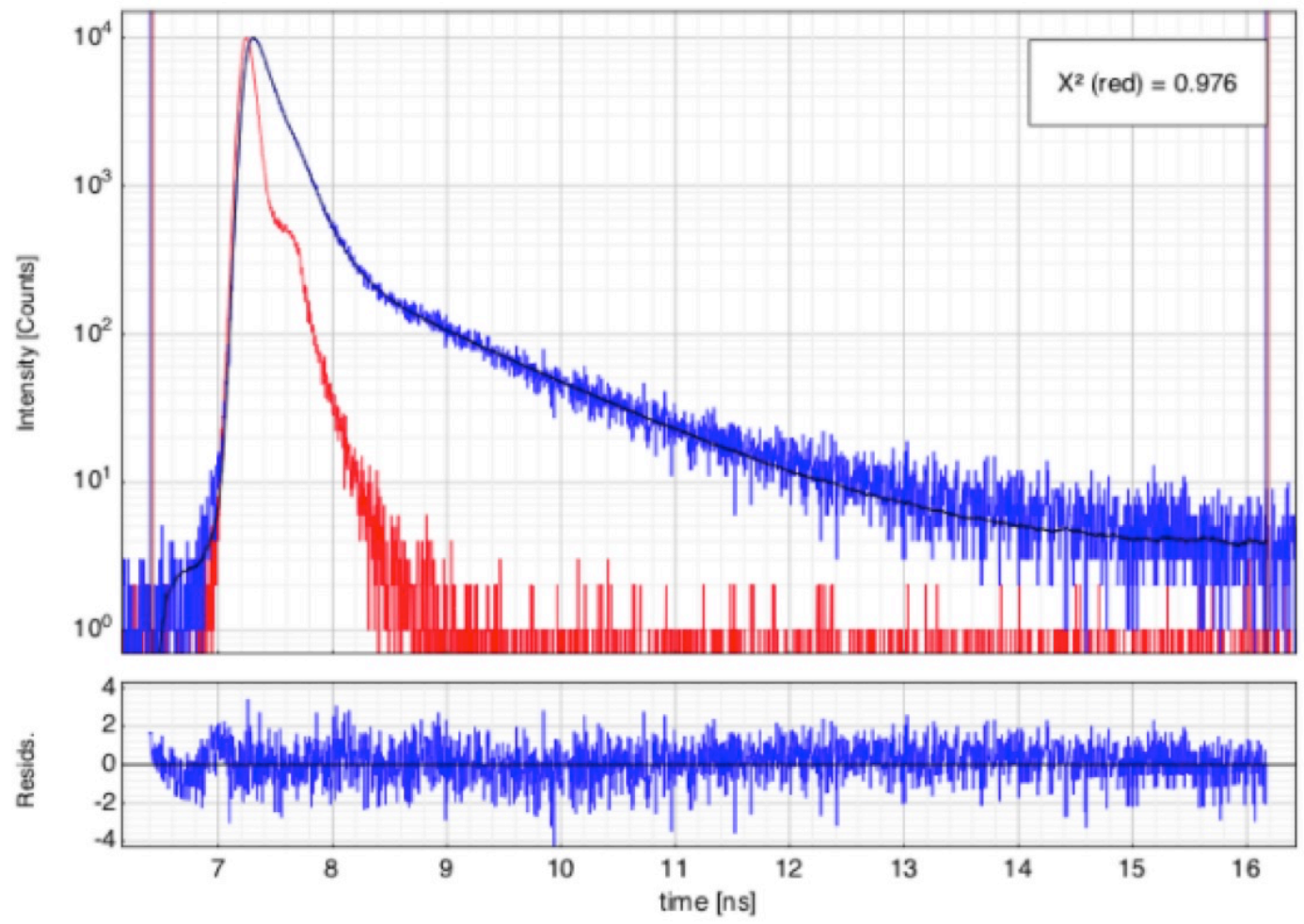


| Parameter | Value | Conf. Lower | Conf. Upper | Conf. Estimation |
|---|---|---|---|---|
| $A_1$ [Cnts] | 418.9 | -12.7 | +12.7 | Fitting |
| $\tau_1$ [ns] | 1.2048 | -0.0224 | +0.0224 | Fitting |
| $A_2$ [Cnts] | -3094 | -209 | +209 | Fitting |
| $\tau_2$ [ns] | 0.13769 | -0.00793 | +0.00793 | Fitting |
| $A_3$ [Cnts] | 24500 | -209 | +209 | Fitting |
| $\tau_3$ [ns] | 0.13743 | -0.00100 | +0.00100 | Fitting |
| Bkgr. $_{Dec}$ [Cnts] | -0.798 | -0.515 | +0.515 | Fitting |
| Bkgr. $_{IRF}$ [Cnts] | -1.864 | -0.262 | +0.262 | Fitting |
| Shift $_{IRF}$ [ns] | -0.005317 | -0.000984 | +0.000984 | Fitting |
| A $_{Scat}$ [Cnts] | 47100 | -4820 | +4820 | Fitting |

Average Lifetime:

$\tau_{Av.1}$=0.2937 ns (intensity weighted)

$\tau_{Av.2}$=0.1579 ns (amplitude weighted)

Fractional Intensities of the Positive Decay Components:

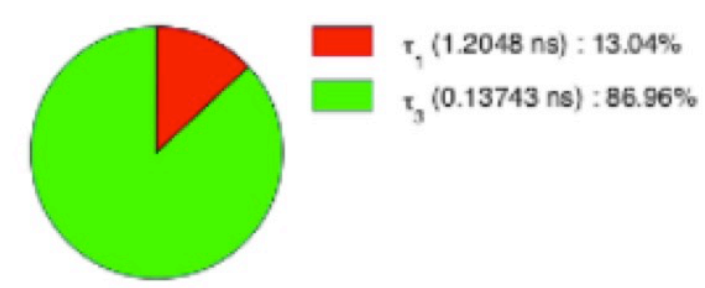


Fractional Amplitudes of the Positive Decay Components:

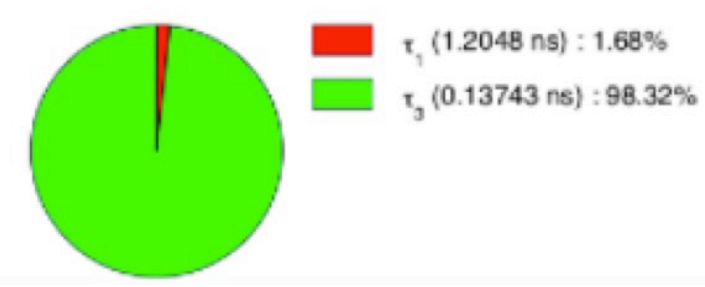


## Dye 1 in glycerol/MeOH (6/4) mixture

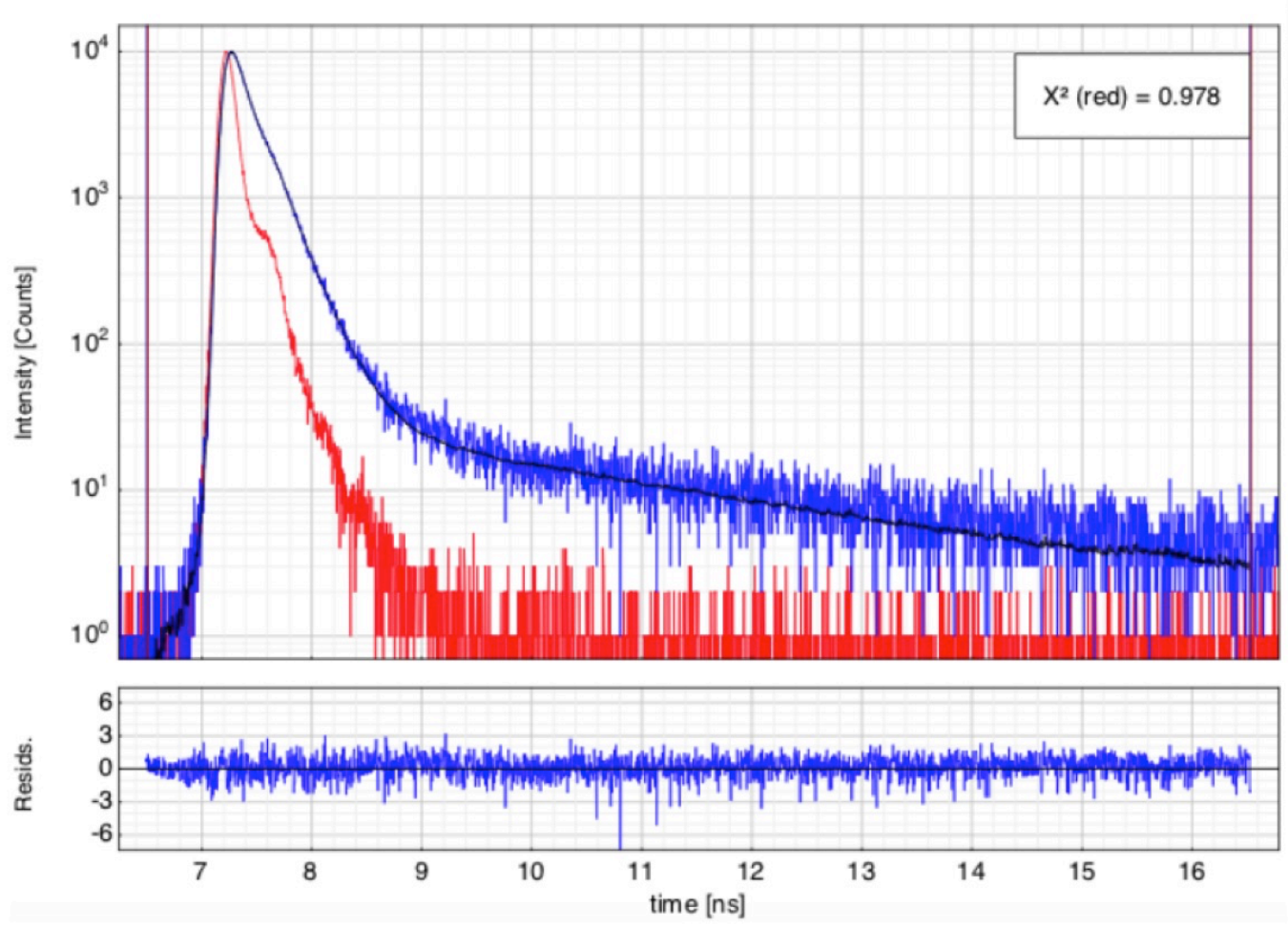


| Parameter | Value | Conf. Lower | Conf. Upper | Conf. Estimation |
|---|---|---|---|---|
| $A_1$ [Cnts] | 8030 | -139 | +139 | Fitting |
| $\tau_1$ [ns] | 0.20082 | -0.00243 | +0.00243 | Fitting |
| $A_2$ [Cnts] | 32.15 | -2.50 | +2.50 | Fitting |
| $\tau_2$ [ns] | 3.105 | -0.197 | +0.197 | Fitting |
| $A_3$ [Cnts] | 14617 | -335 | +335 | Fitting |
| $\tau_3$ [ns] | 0.08636 | -0.00183 | +0.00183 | Fitting |
| Bkgr. $_{Dec}$ [Cnts] | -0.162 | -0.469 | +0.469 | Fitting |
| Bkgr. $_{IRF}$ [Cnts] | -0.399 | -0.260 | +0.260 | Fitting |
| Shift $_{IRF}$ [ns] | 0.005576 | -0.000953 | +0.000953 | Fitting |
| A $_{Scat}$ [Cnts] | 109230 | -5440 | +5440 | Fitting |

Average Lifetime:

$\tau_{Av.1}$=0.24970 ns (intensity weighted)

$\tau_{Av.2}$=0.13116 ns (amplitude weighted)

Fractional Intensities of the Positive Decay Components:

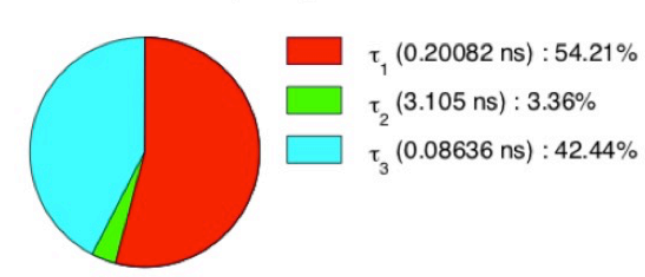


Fractional Amplitudes of the Positive Decay Components:

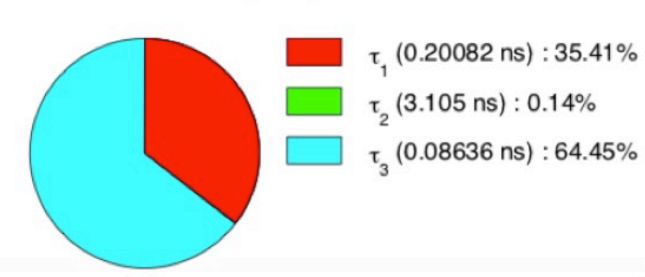


**Figure S13 (cont'd)**. Fluorescence intensity decays of **1** in solvents of various viscosities.

## Dye 1 in tetraethylene glycol

| Parameter | Value | Conf. Lower | Conf. Upper | Conf. Estimation |
|---|---|---|---|---|
| $A_1$ [Cnts] | 137.72 | -5.32 | +5.32 | Fitting |
| $\tau_1$ [ns] | 4.750 | -0.136 | +0.136 | Fitting |
| $A_2$ [Cnts] | 10180 | -125 | +125 | Fitting |
| $\tau_2$ [ns] | 0.44318 | -0.00411 | +0.00411 | Fitting |
| $A_3$ [Cnts] | 6249 | -235 | +235 | Fitting |
| $\tau_3$ [ns] | 0.21055 | -0.00829 | +0.00829 | Fitting |
| Bkgr. Dec [Cnts] | 0.813 | -0.671 | +0.671 | Fitting |
| Bkgr. IRF [Cnts] | -0.978 | -0.186 | +0.186 | Fitting |
| Shift IRF [ns] | -0.02308 | -0.00168 | +0.00168 | Fitting |
| A Scat [Cnts] | -43880 | -5240 | +5240 | Fitting |

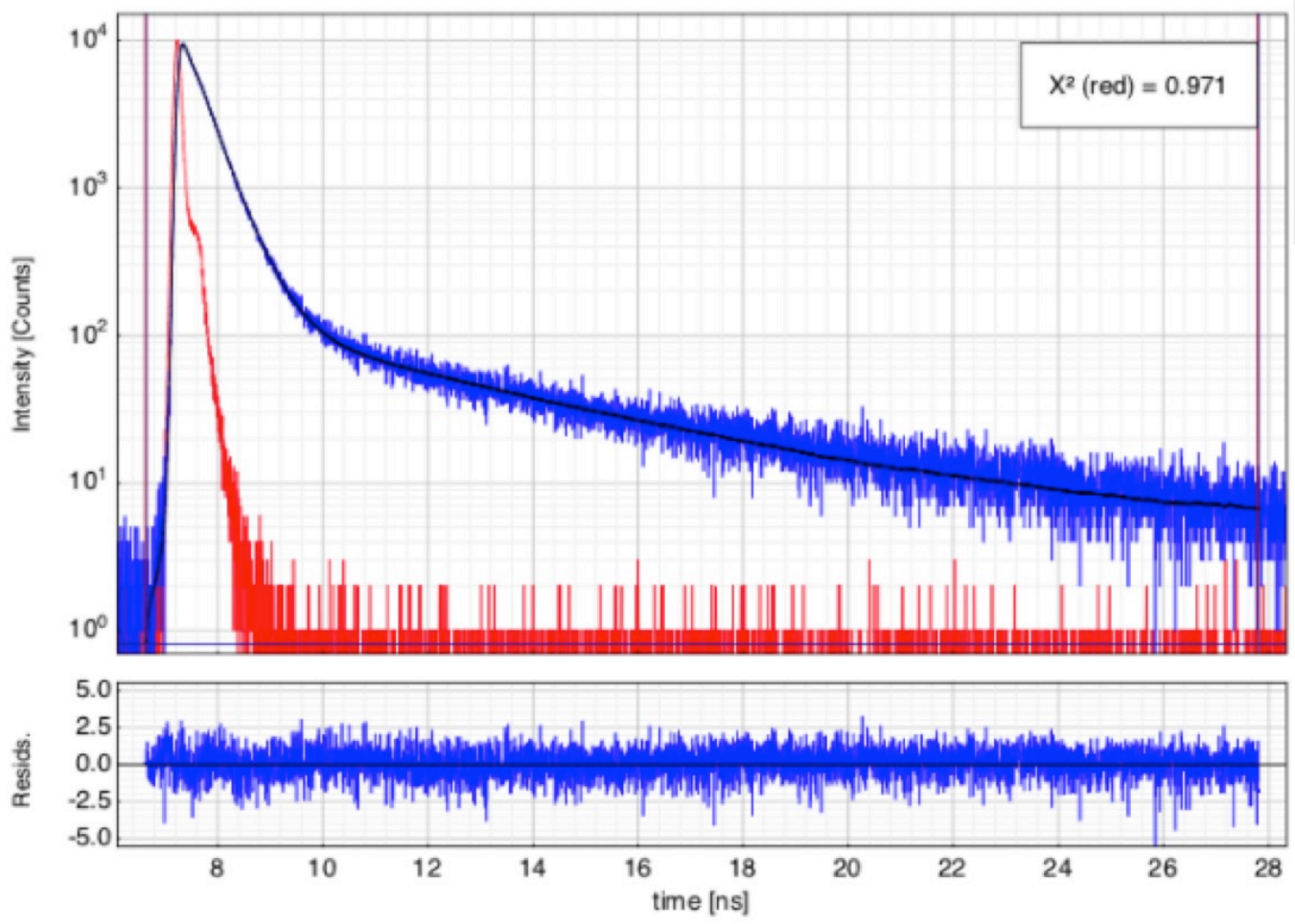


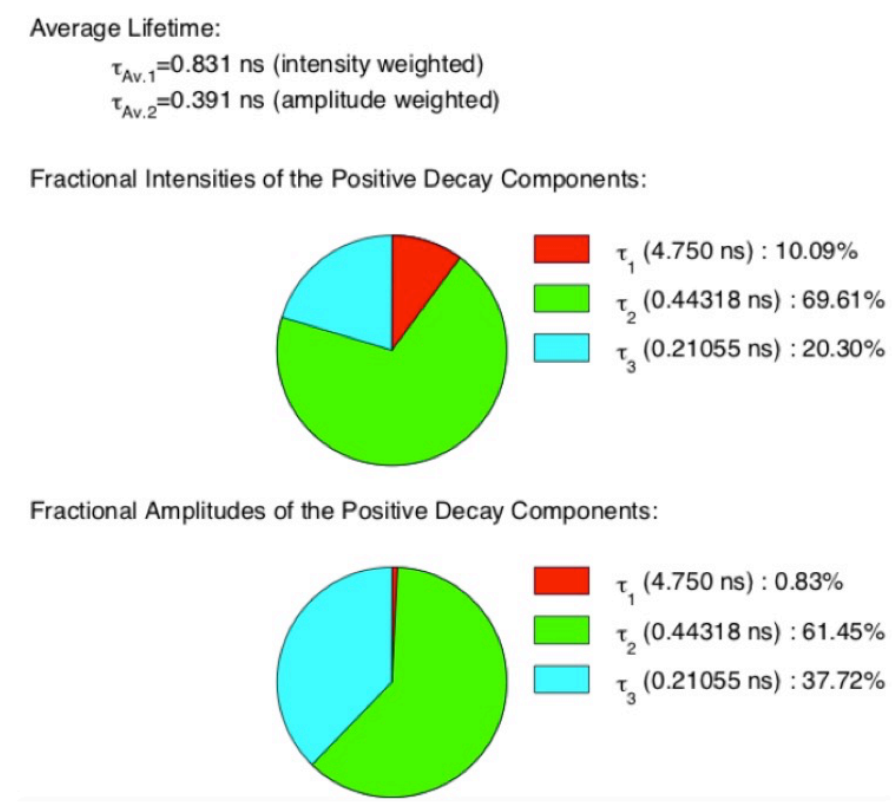


## Dye 1 in glycerol/MeOH (7/3) mixture

| Parameter | Value | Conf. Lower | Conf. Upper | Conf. Estimation |
|---|---|---|---|---|
| $A_1$ [Cnts] | 14070.1 | -91.0 | +91.0 | Fitting |
| $\tau_1$ [ns] | 0.22293 | -0.00124 | +0.00124 | Fitting |
| $A_2$ [Cnts] | 2600.4 | -41.1 | +41.1 | Fitting |
| $\tau_2$ [ns] | 0.45473 | -0.00411 | +0.00411 | Fitting |
| $A_3$ [Cnts] | 6193 | -230 | +230 | Fitting |
| $\tau_3$ [ns] | 0.06955 | -0.00296 | +0.00296 | Fitting |
| Bkgr. Dec [Cnts] | 17.18 | -1.29 | +1.29 | Fitting |
| Bkgr. IRF [Cnts] | 0.586 | -0.493 | +0.493 | Fitting |
| Shift IRF [ns] | -0.022819 | -0.000657 | +0.000657 | Fitting |
| A Scat [Cnts] | -51610 | -2470 | +2470 | Fitting |

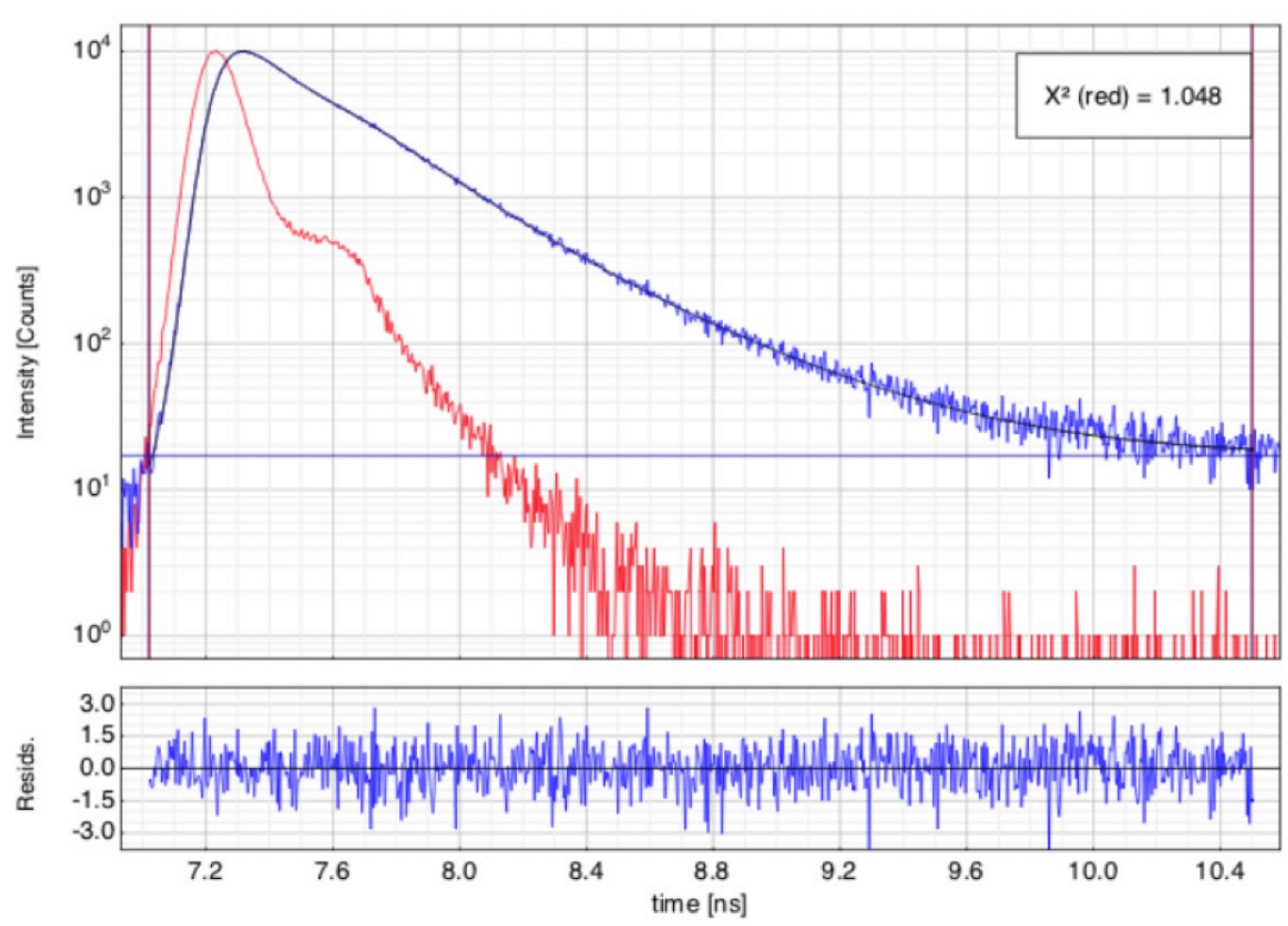


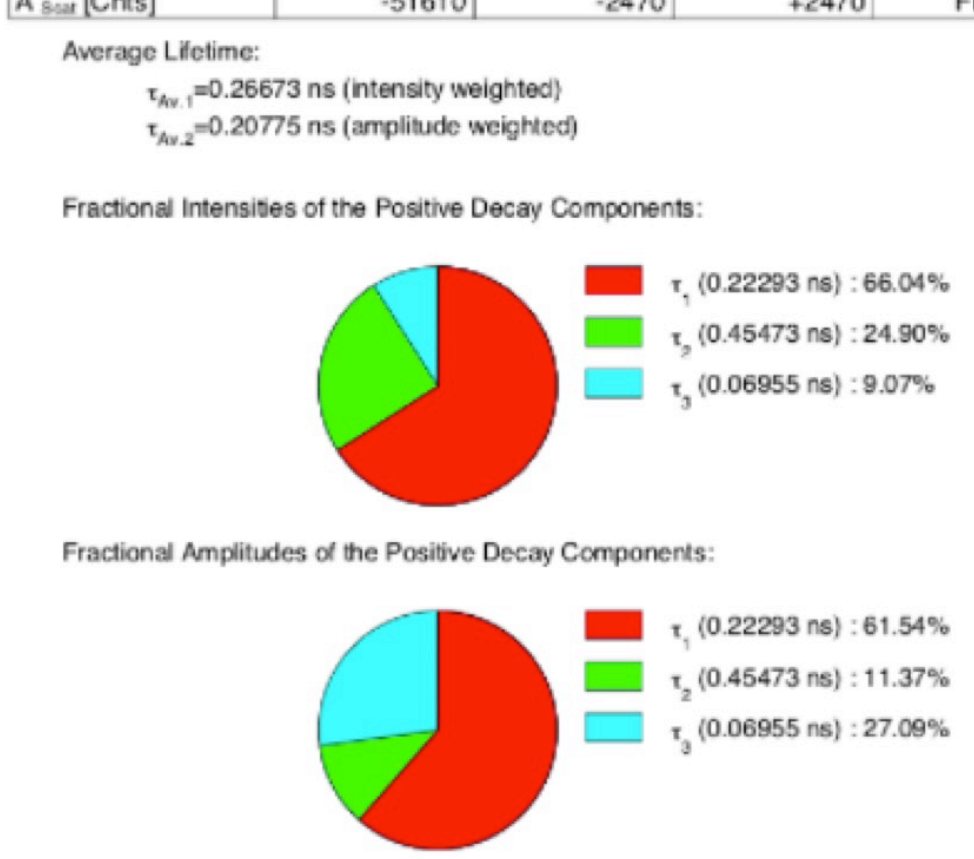


**Figure S13 (cont'd)**. Fluorescence intensity decays of **1** in solvents of various viscosities.

**Dye 1 in glycerol/MeOH (8/2) mixture**

| Parameter | Value | Conf. $_{Lower}$ | Conf. $_{Upper}$ | Conf. $_{Estimation}$ |
|---|---|---|---|---|
| $A_1$ [Cnts] | 168.8 | -10.7 | +10.7 | Fitting |
| $\tau_1$ [ns] | 1.7556 | -0.0572 | +0.0572 | Fitting |
| $A_2$ [Cnts] | 10370.0 | -67.4 | +67.4 | Fitting |
| $\tau_2$ [ns] | 0.53029 | -0.00249 | +0.00249 | Fitting |
| $A_3$ [Cnts] | 6571 | -148 | +148 | Fitting |
| $\tau_3$ [ns] | 0.20623 | -0.00504 | +0.00504 | Fitting |
| Bkgr. $_{Dec}$ [Cnts] | -0.652 | -0.705 | +0.705 | Fitting |
| Bkgr. $_{IRF}$ [Cnts] | -1.308 | -0.226 | +0.226 | Fitting |
| Shift $_{IRF}$ [ns] | -0.023389 | -0.000988 | +0.000988 | Fitting |
| A $_{Scat}$ [Cnts] | -37560 | -3280 | +3280 | Fitting |

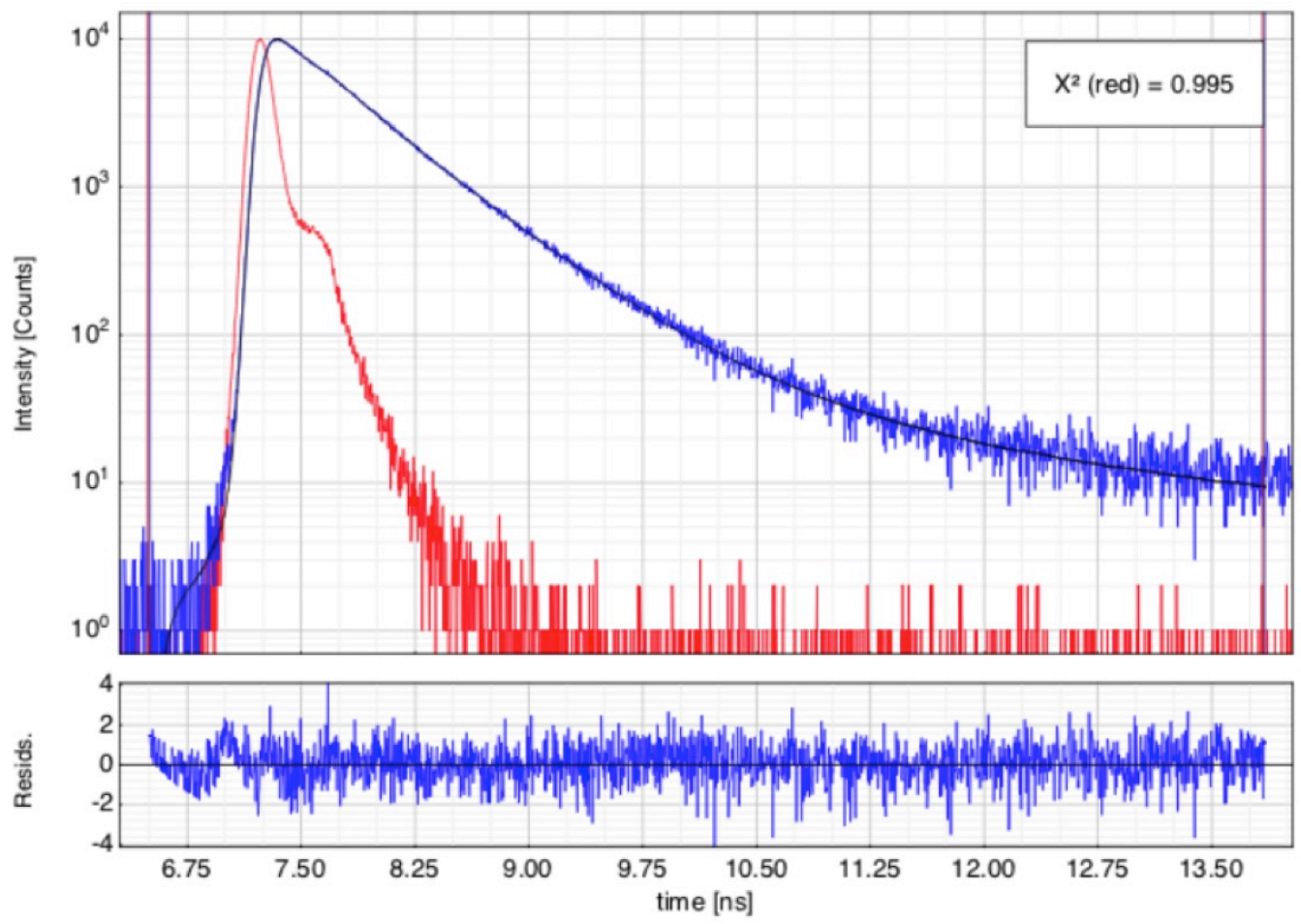


Average Lifetime:

$\tau_{Av.1}$=0.5197 ns (intensity weighted)

$\tau_{Av.2}$=0.4179 ns (amplitude weighted)

Fractional Intensities of the Positive Decay Components:

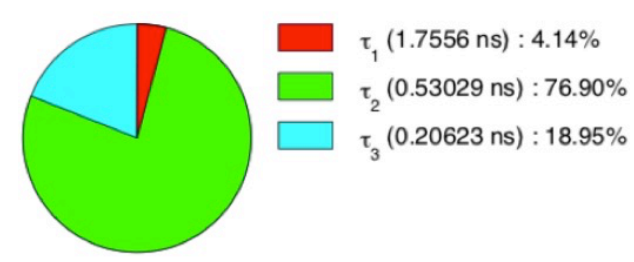


Fractional Amplitudes of the Positive Decay Components:

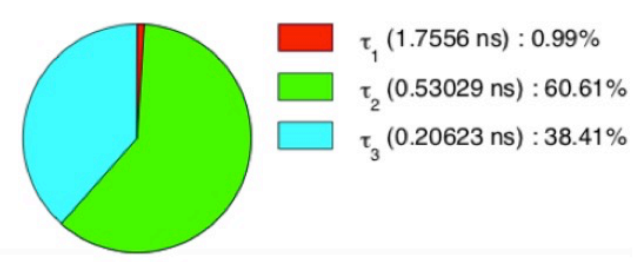


**Dye 1 in glycerol/MeOH (9/1) mixture**

| Parameter | Value | Conf. $_{Lower}$ | Conf. $_{Upper}$ | Conf. $_{Estimation}$ |
|---|---|---|---|---|
| $A_1$ [Cnts] | 7125.0 | -51.0 | +51.0 | Fitting |
| $\tau_1$ [ns] | 1.02556 | -0.00477 | +0.00477 | Fitting |
| $A_2$ [Cnts] | 6796.0 | -93.3 | +93.3 | Fitting |
| $\tau_2$ [ns] | 0.53426 | -0.00700 | +0.00700 | Fitting |
| Bkgr. $_{Dec}$ [Cnts] | -1.224 | -0.703 | +0.703 | Fitting |
| Bkgr. $_{IRF}$ [Cnts] | -1.130 | -0.145 | +0.145 | Fitting |
| Shift $_{IRF}$ [ns] | 0.04083 | -0.00121 | +0.00121 | Fitting |
| A $_{Scat}$ [Cnts] | -4990 | -3950 | +3950 | Fitting |

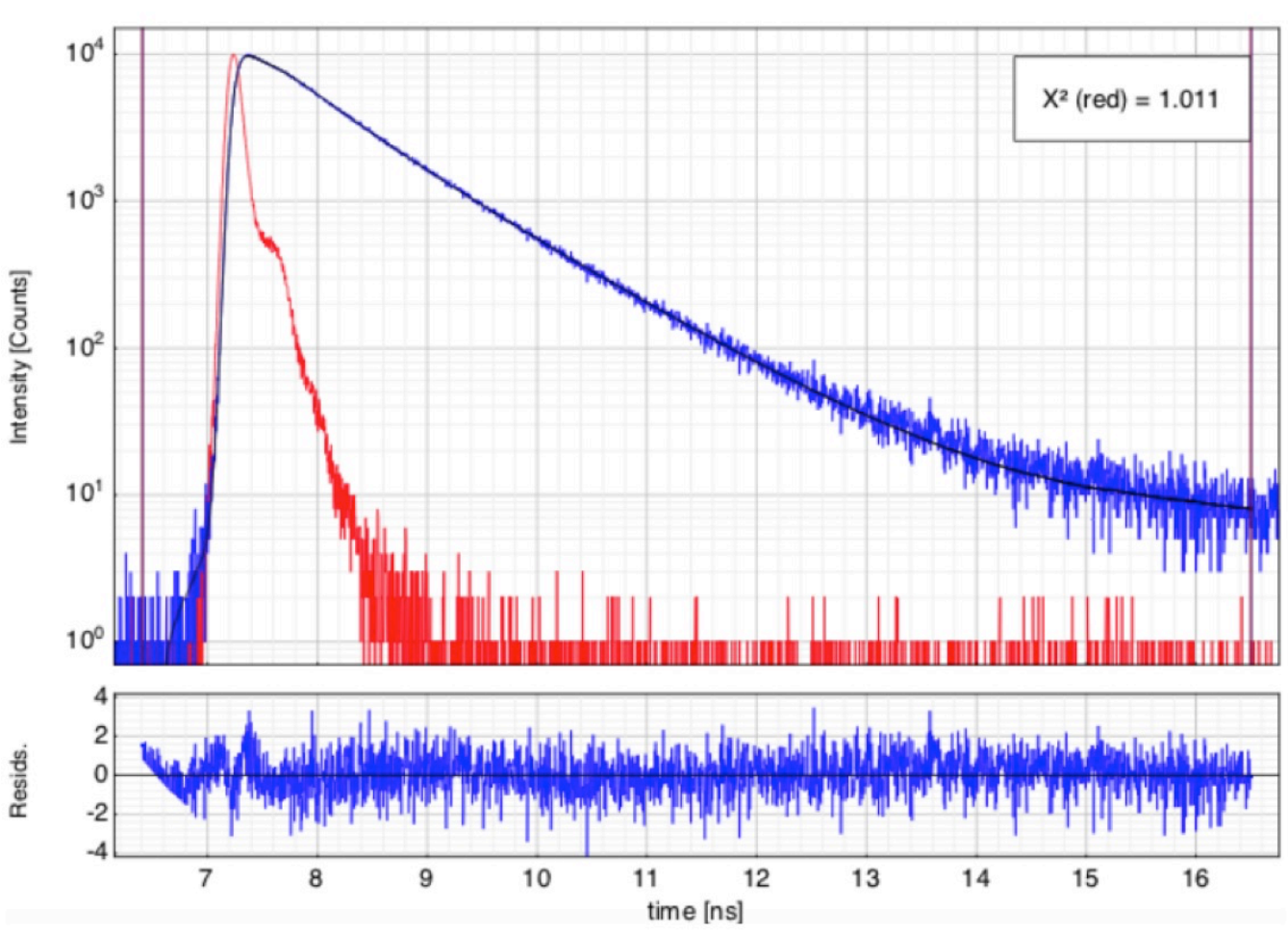


Average Lifetime:

$\tau_{Av.1}$=0.86247 ns (intensity weighted)

$\tau_{Av.2}$=0.78572 ns (amplitude weighted)

Fractional Intensities of the Positive Decay Components:

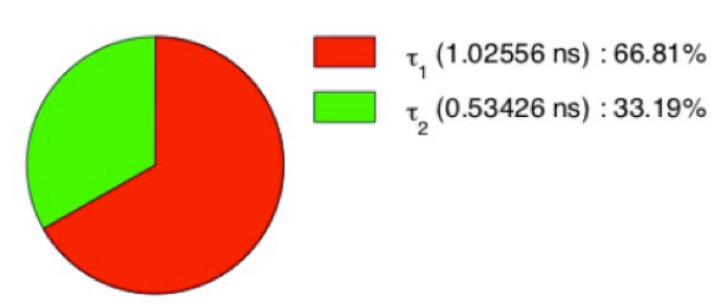


Fractional Amplitudes of the Positive Decay Components:

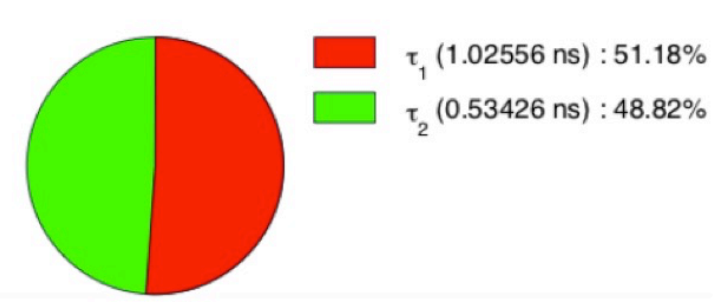


**Figure S13 (cont'd)**. Fluorescence intensity decays of **1** in solvents of various viscosities.

## Dye 1 in glycerol

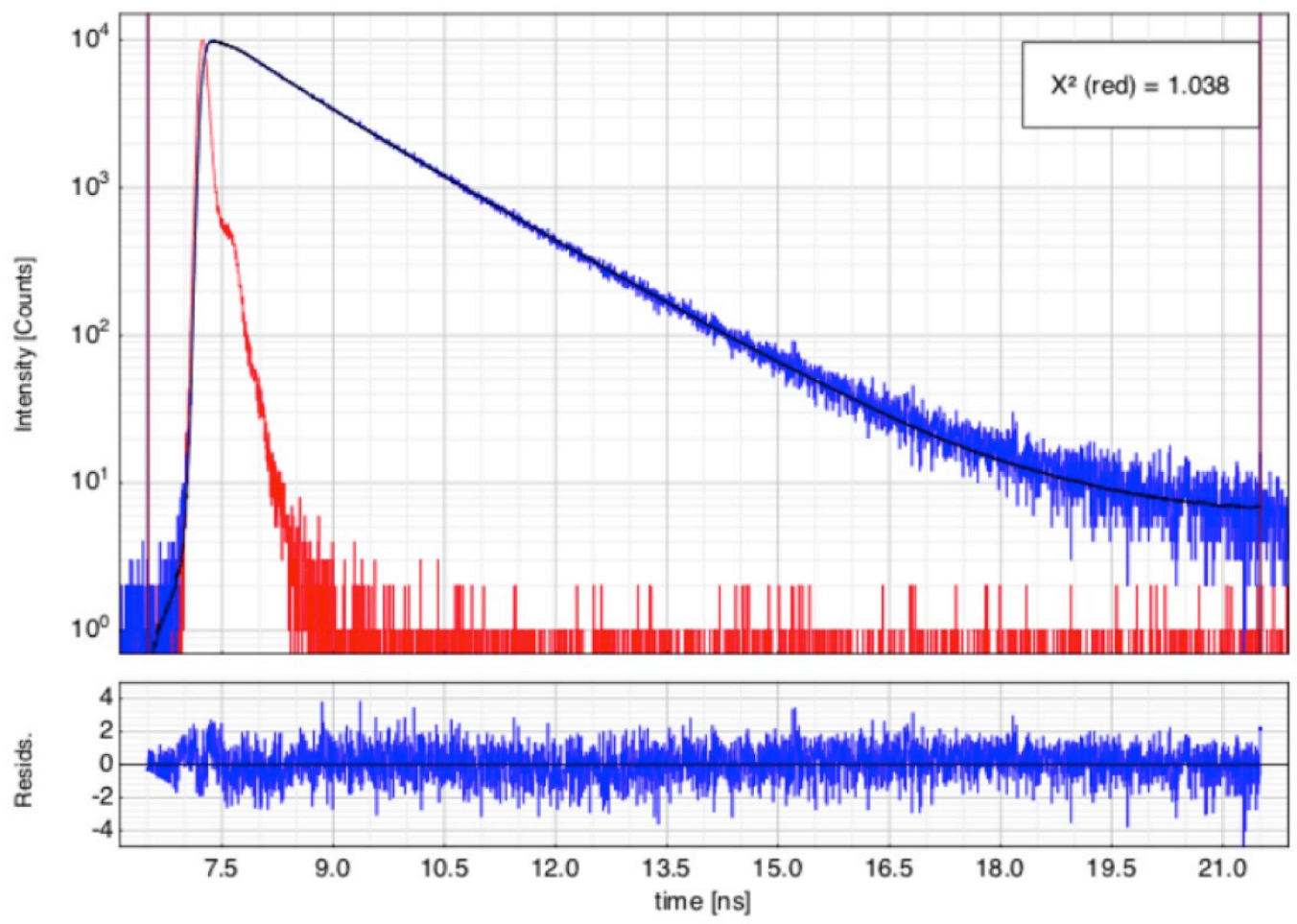


| Parameter | Value | Conf. Lower | Conf. Upper | Conf. Estimation |
|---|---|---|---|---|
| $A_1$ [Cnts] | 9723.2 | -53.8 | +53.8 | Fitting |
| $\tau_1$ [ns] | 1.51696 | -0.00581 | +0.00581 | Fitting |
| $A_2$ [Cnts] | 3018 | -103 | +103 | Fitting |
| $\tau_2$ [ns] | 0.7038 | -0.0248 | +0.0248 | Fitting |
| Bkgr. $_{Dec}$ [Cnts] | 0.336 | -0.771 | +0.771 | Fitting |
| Bkgr. $_{IRF}$ [Cnts] | -0.3900 | -0.0876 | +0.0876 | Fitting |
| Shift $_{IRF}$ [ns] | 0.04324 | -0.00146 | +0.00146 | Fitting |
| A $_{Scat}$ [Cnts] | 50220 | -5550 | +5550 | Fitting |

Average Lifetime:

$\tau_{Av.1}$=1.41459 ns (intensity weighted)

$\tau_{Av.2}$=1.32434 ns (amplitude weighted)

Fractional Intensities of the Positive Decay Components:

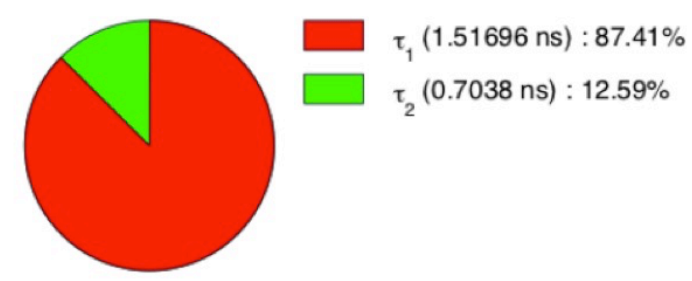


Fractional Amplitudes of the Positive Decay Components:

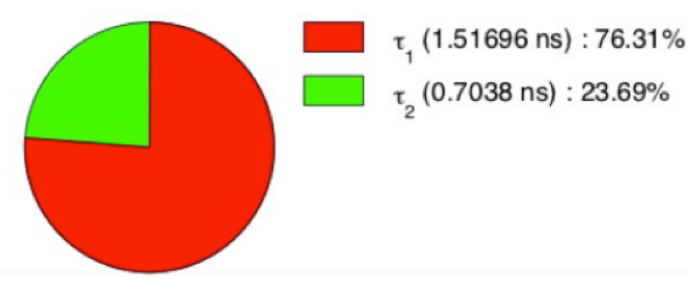


**Figure S13 (cont'd)**. Fluorescence intensity decays of **1** in solvents of various viscosities.

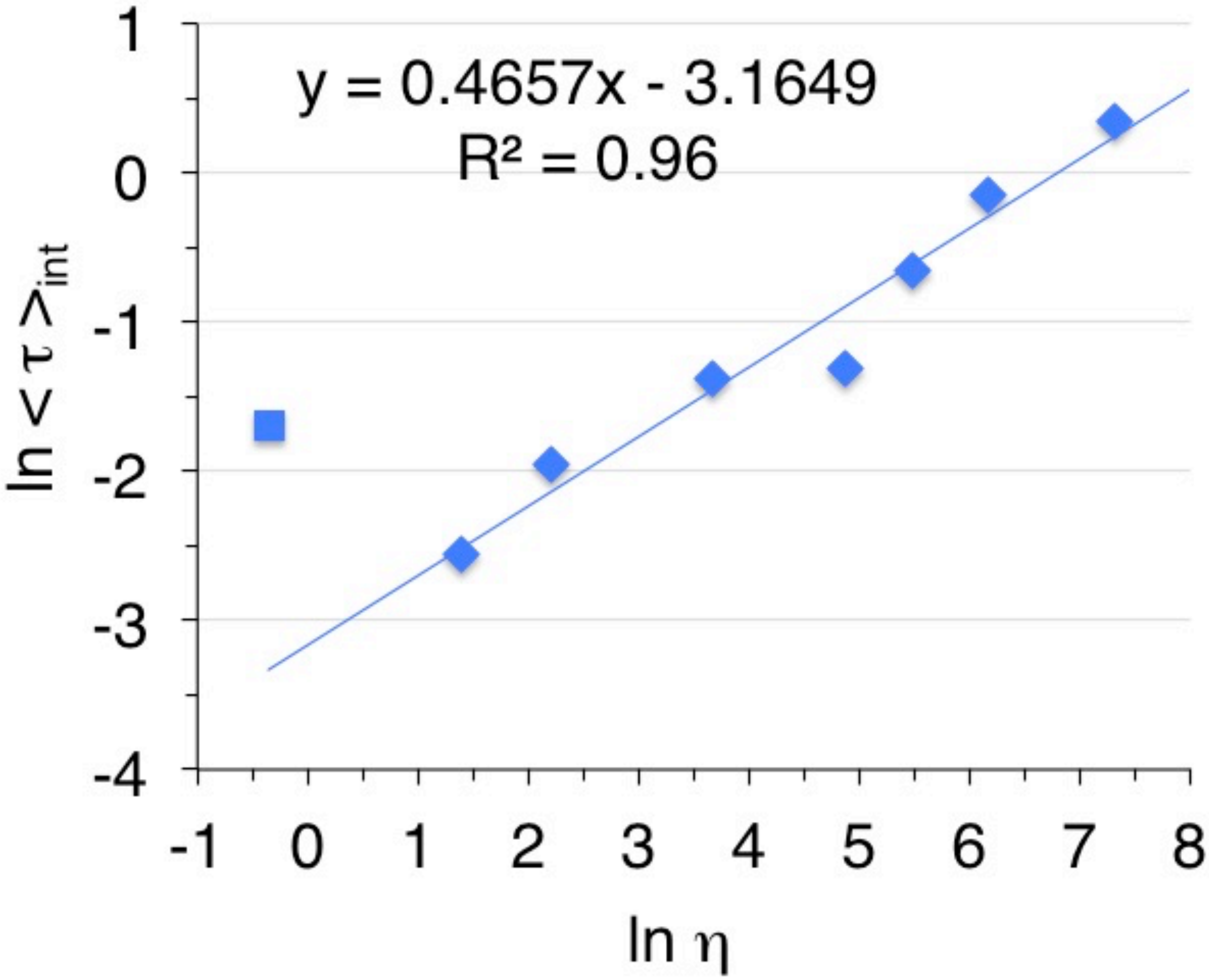


**Figure 14**. Intensity average lifetimes of dye **1** as a function of media's viscosity.

MeOH/square was not included in the correlation.
For viscosities of the solvents and related lifetimes of dye **1** see Table S5.

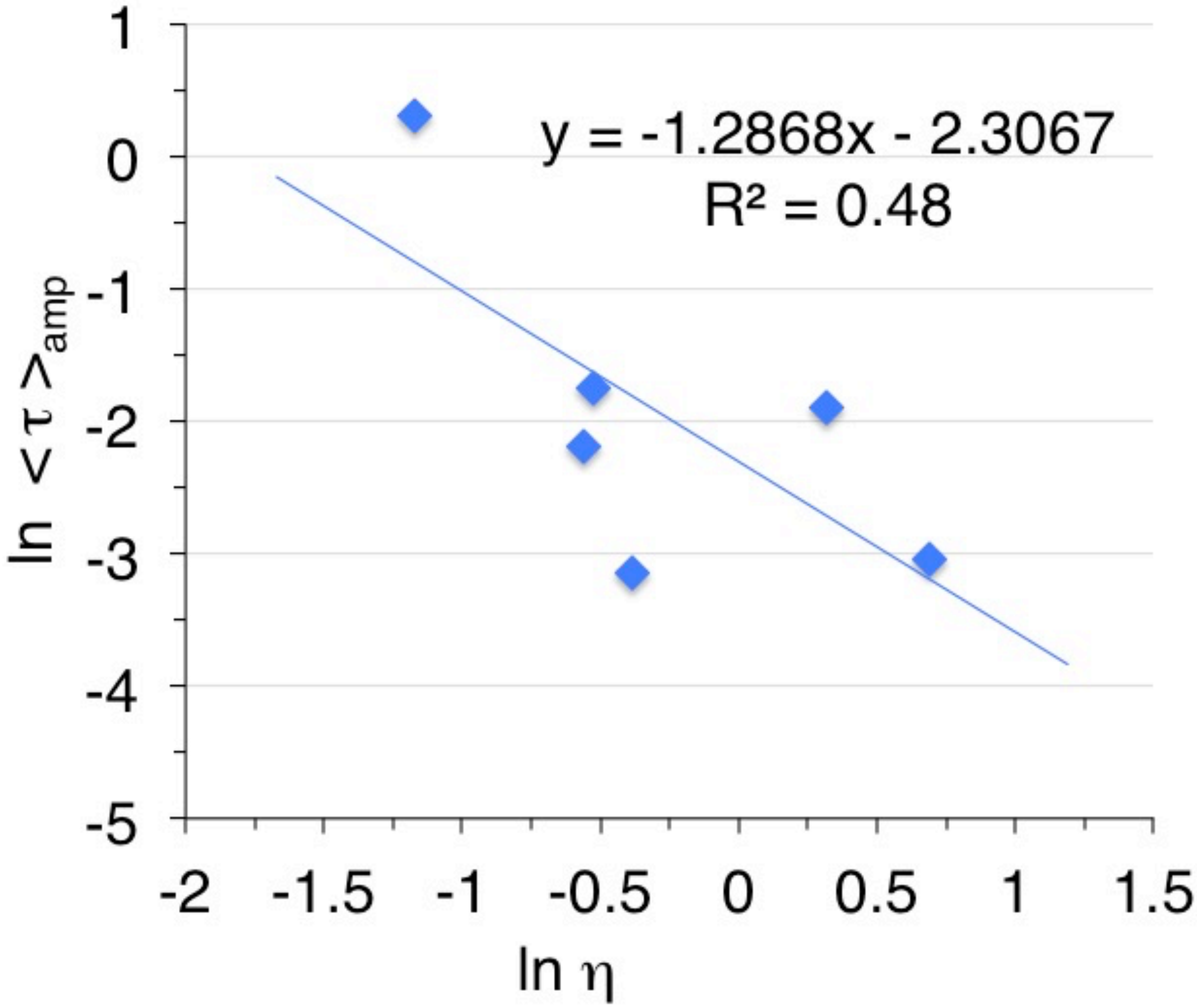


**Figure S15**. Amplitude average lifetimes of dye **1** as a function of viscosity of low-viscosity organic solvents.

Low-viscosity organic solvents: MeOH, dioxane, DMSO, toluene, hexane, chloroform.
For viscosities of the solvents and related lifetimes of dye **1** see Table S4.

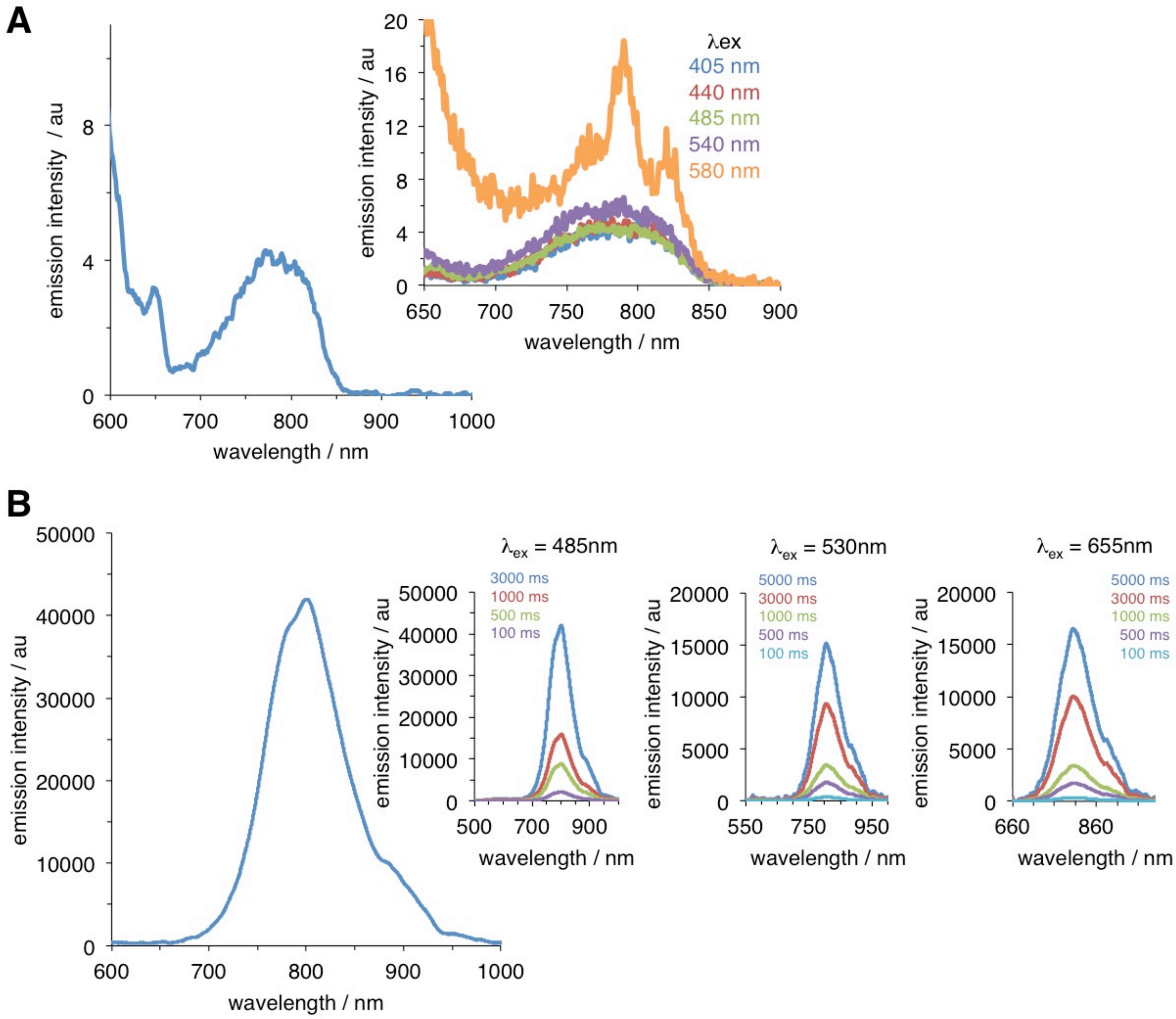


**Figure S16**. Solid-state emission spectra of **1** using various systems and conditions.

**A**: Cary Eclipse spectrophotometer; Conditions: front face, powdered sample of dye **1** in 1 mm pathlength cuvette; λex = 480 nm, 600 long pass filter. Inset: solid-state emission spectra of **1** at various excitation wavelengths.

**B**: OCEANOPTICS, powder sample of dye **1**. Integration time: 3000 ms; λex = 485nm. Inset: solid-state emission spectra of **1** at various excitations wavelengths, integrations times are shown on each spectrum.

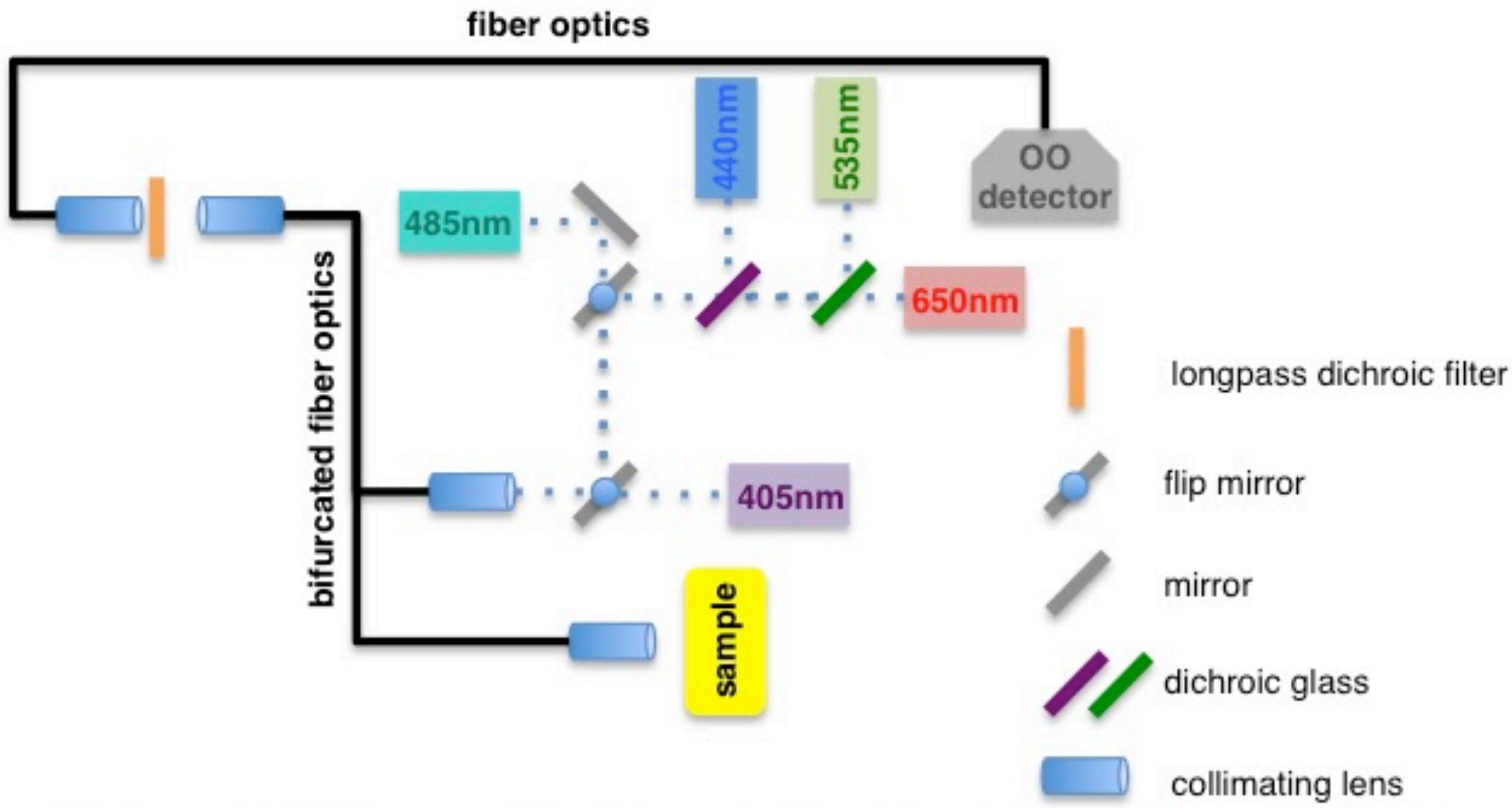


**Figure S17**. OCEANOPTICS set-up.

Schematics of the set-up; rectangles with numbers are lasers with specific wavelengths (i.e., 405, 440, 485, 535, and 650 nm).

| Parameter | Value | Conf. $_{Lower}$ | Conf. $_{Upper}$ | Conf. $_{Estimation}$ |
|---|---|---|---|---|
| $A_1$ [Cnts] | 14539 | -144 | +144 | Fitting |
| $\tau_1$ [ns] | 0.20657 | -0.00188 | +0.00188 | Fitting |
| $A_2$ [Cnts] | 4058.9 | -59.7 | +59.7 | Fitting |
| $\tau_2$ [ns] | 0.47950 | -0.00423 | +0.00423 | Fitting |
| Bkgr. $_{Dec}$ [Cnts] | 17.51 | -1.01 | +1.01 | Fitting |
| Bkgr. $_{IRF}$ [Cnts] | 0.00244 | -0.358 | +0.358 | Fitting |
| Shift $_{IRF}$ [ns] | -0.009449 | -0.000951 | +0.000951 | Fitting |

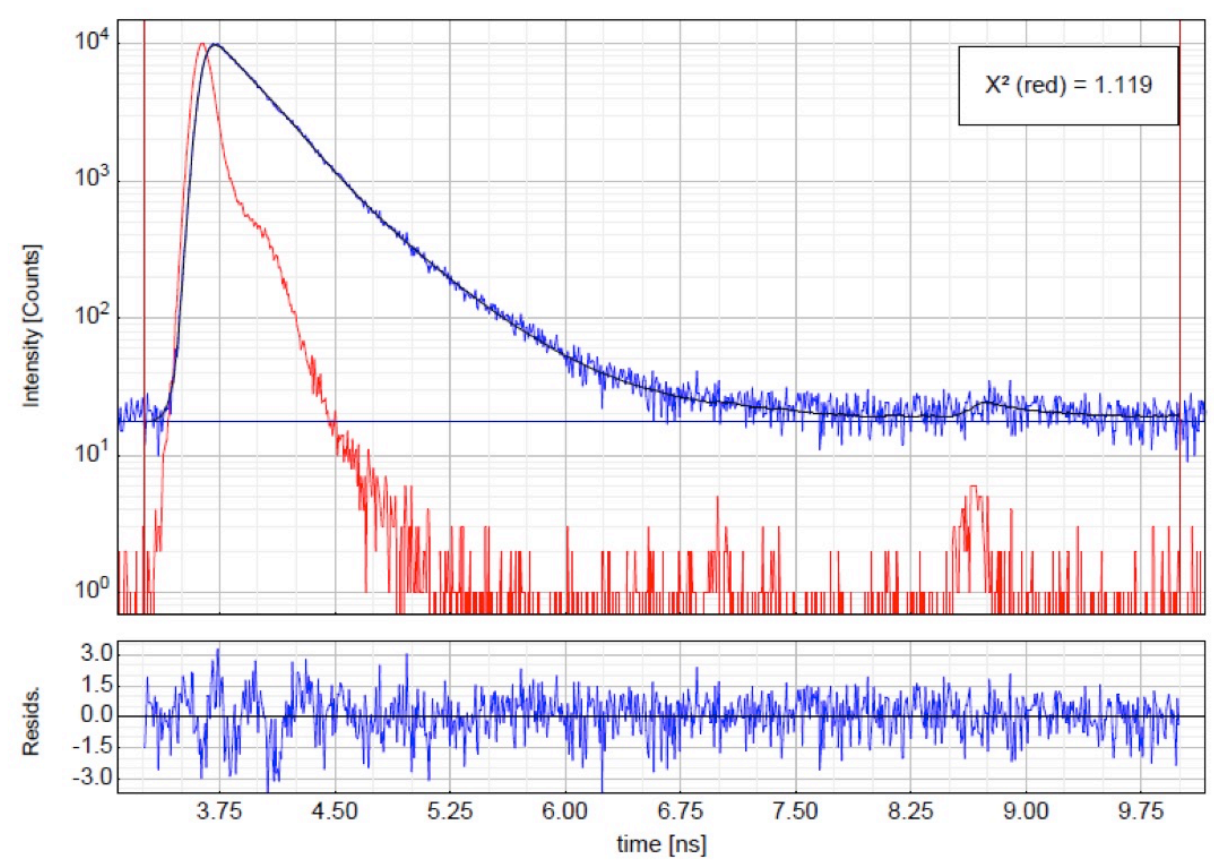


Average Lifetime:

$\tau_{Av.1}$=0.31389 ns (intensity weighted)

$\tau_{Av.2}$=0.26613 ns (amplitude weighted)

Fractional Intensities of the Positive Decay Components:

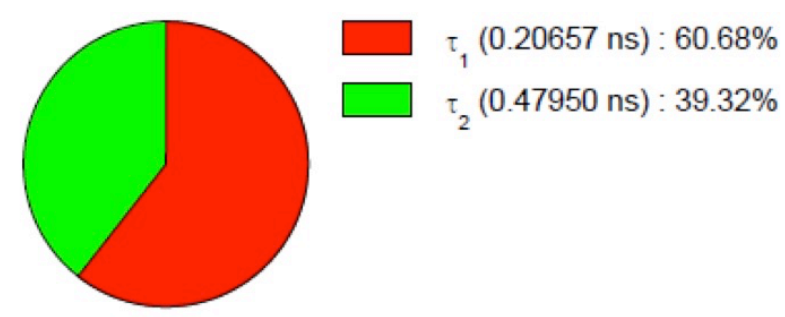


Fractional Amplitudes of the Positive Decay Components:

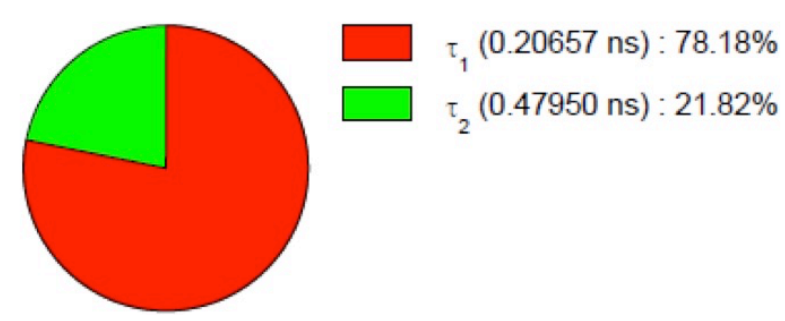


**Figure S18**. Average emission lifetimes of dye **1** in the solid state.

Conditions: λex = 485 nm, λem = 800 nm.

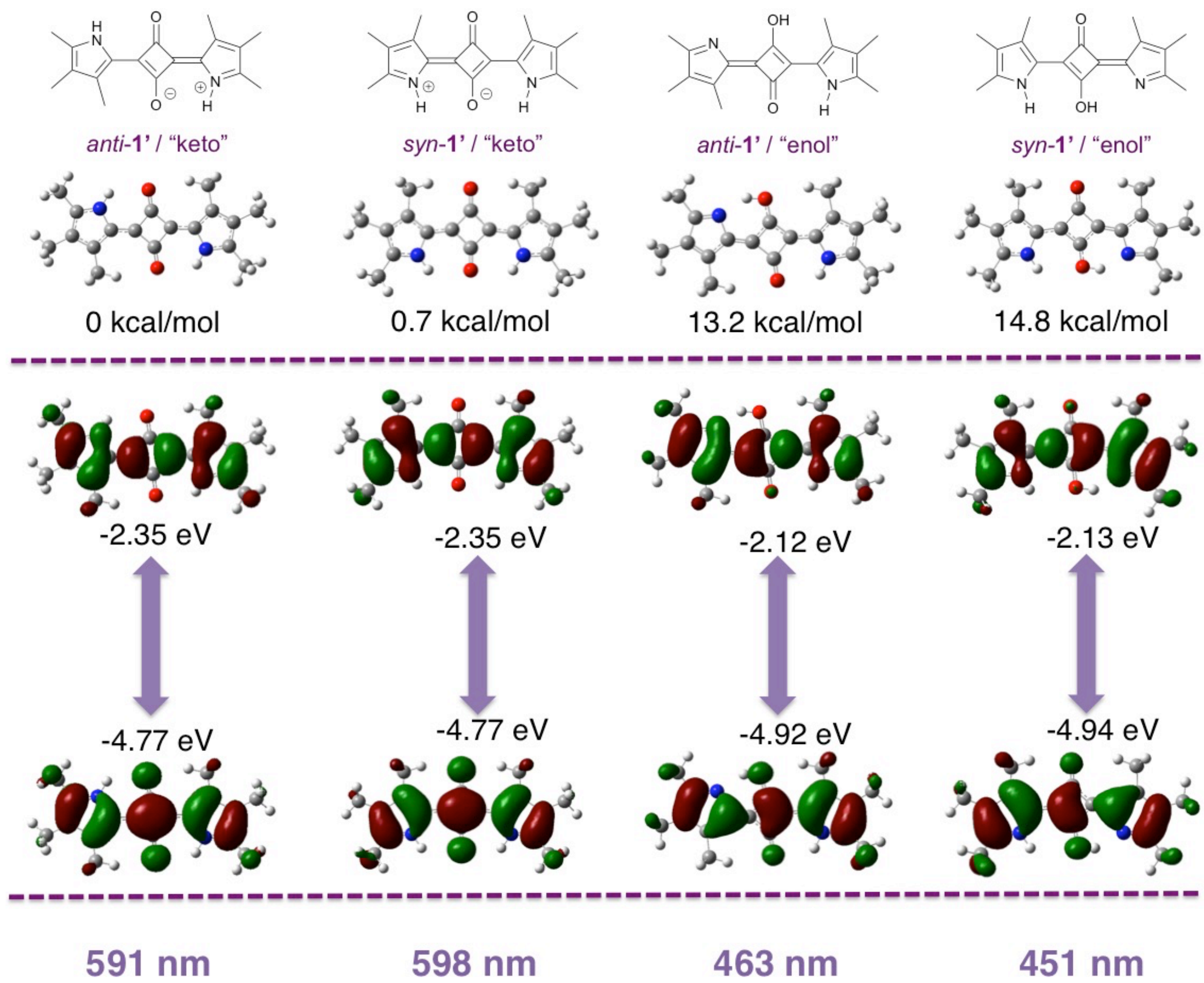


**Figure S19**. Computed energies of ground state structures (top), DFT-predicted HOMO–LUMO (middle), and TDDFT-predicted emission maxima / $\lambda_{em}^{max}$ (bottom) of various conformers and tautomers of dye **1'**.

Conformers of **1'**: anti/syn
Tautomers of **1'**: "keto"/"enol"

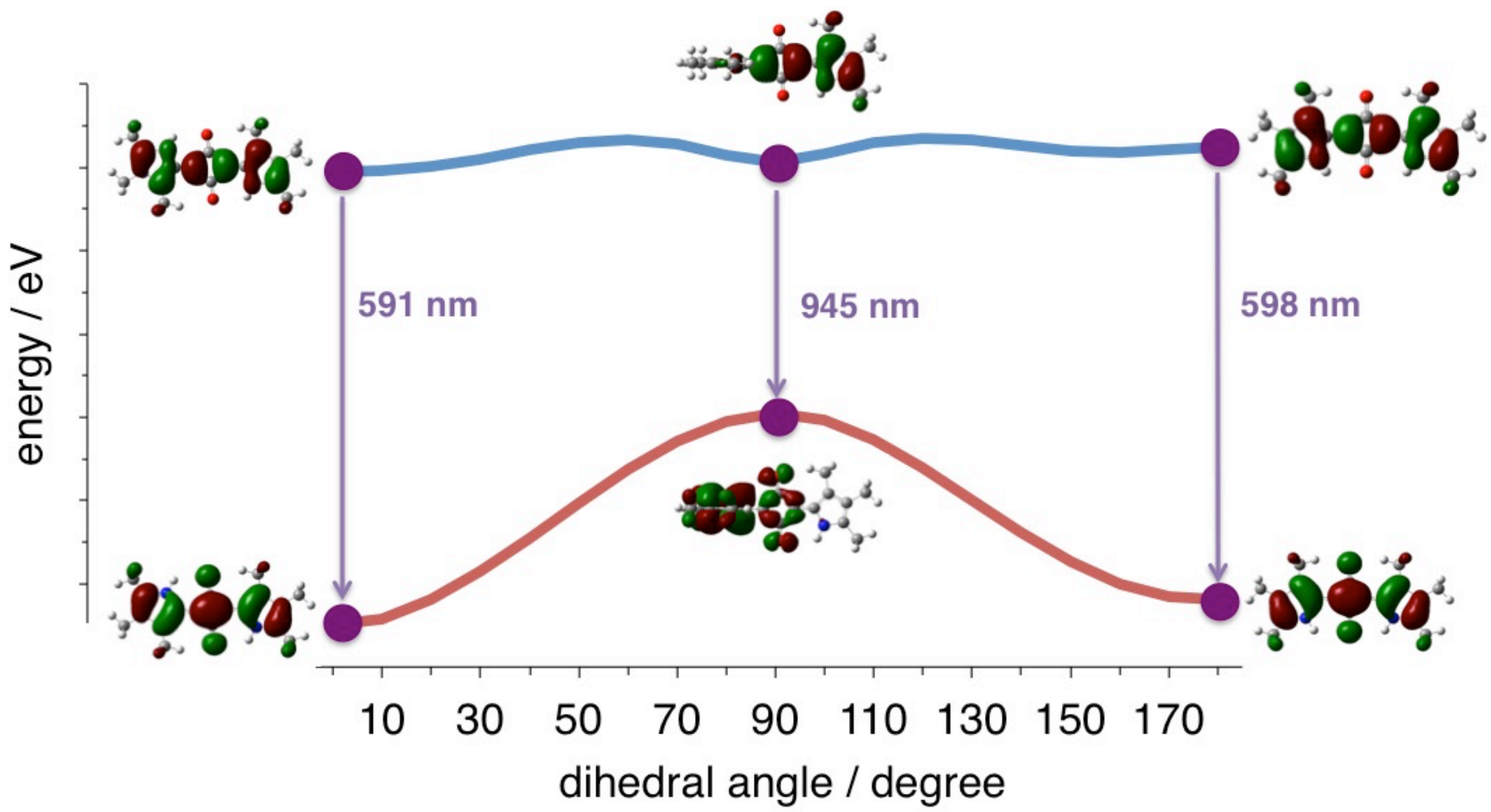


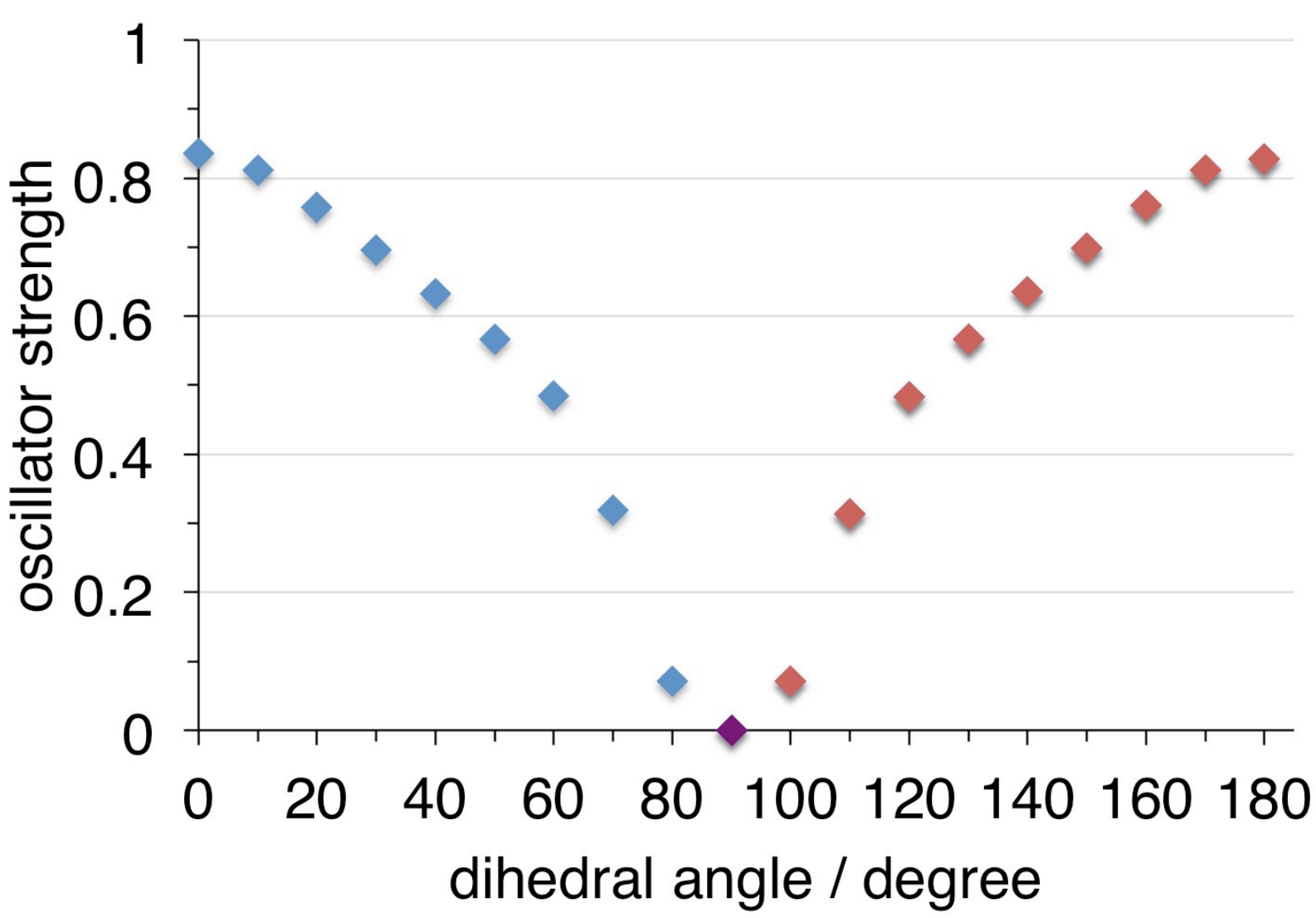


**Figure S20**. Computed energy profile of TICT (twisted intramolecular charge transfer) state and oscillator strength of **1'**.

**Top:** Energy profiles of the ground (red line) and excited state (blue line) of **1'** as a function of dihedral angle. $0^o$, $90^o$, and $180^o$ conformers are marked by purple circles: $0^o$ corresponds to planar *anti*-**1'**, $90^o$ corresponds to twisted **1'**, and $180^o$ corresponds to planar *syn*-**1'**, with the corresponding TDDFT-predicted emission maxima / $\lambda_{em}^{max}$ given next to the arrows.

**Bottom:** Computed oscillator strength as a functional of the dihedral angle of **1'**. Rotation from *anti*-**1'** conformer to $90^o$-conformer (blue symbols), $90^o$ conformer of **1'** (purple symbol), from $90^o$-conformer to *syn*-**1'** conformer (red symbols).

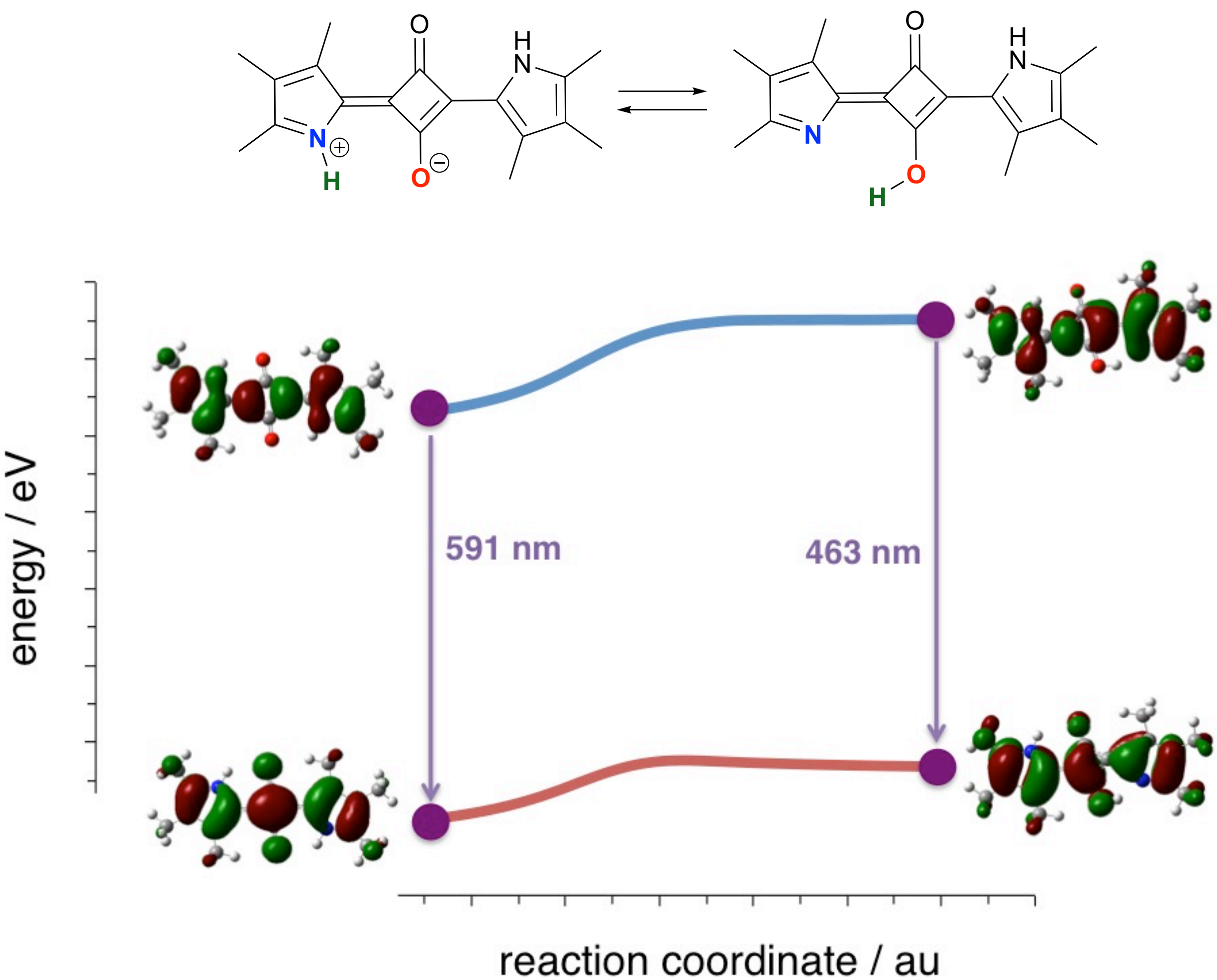


**Figure S21**. Computed energy profile of ESIPT (excited state intramolecular proton-transfer) **1’**. Ground and excited states of keto and enol forms are marked by purple circles, and the corresponding TDFT-predicted emission maxima / $\lambda_{em}^{max}$ are given next to the arrows.

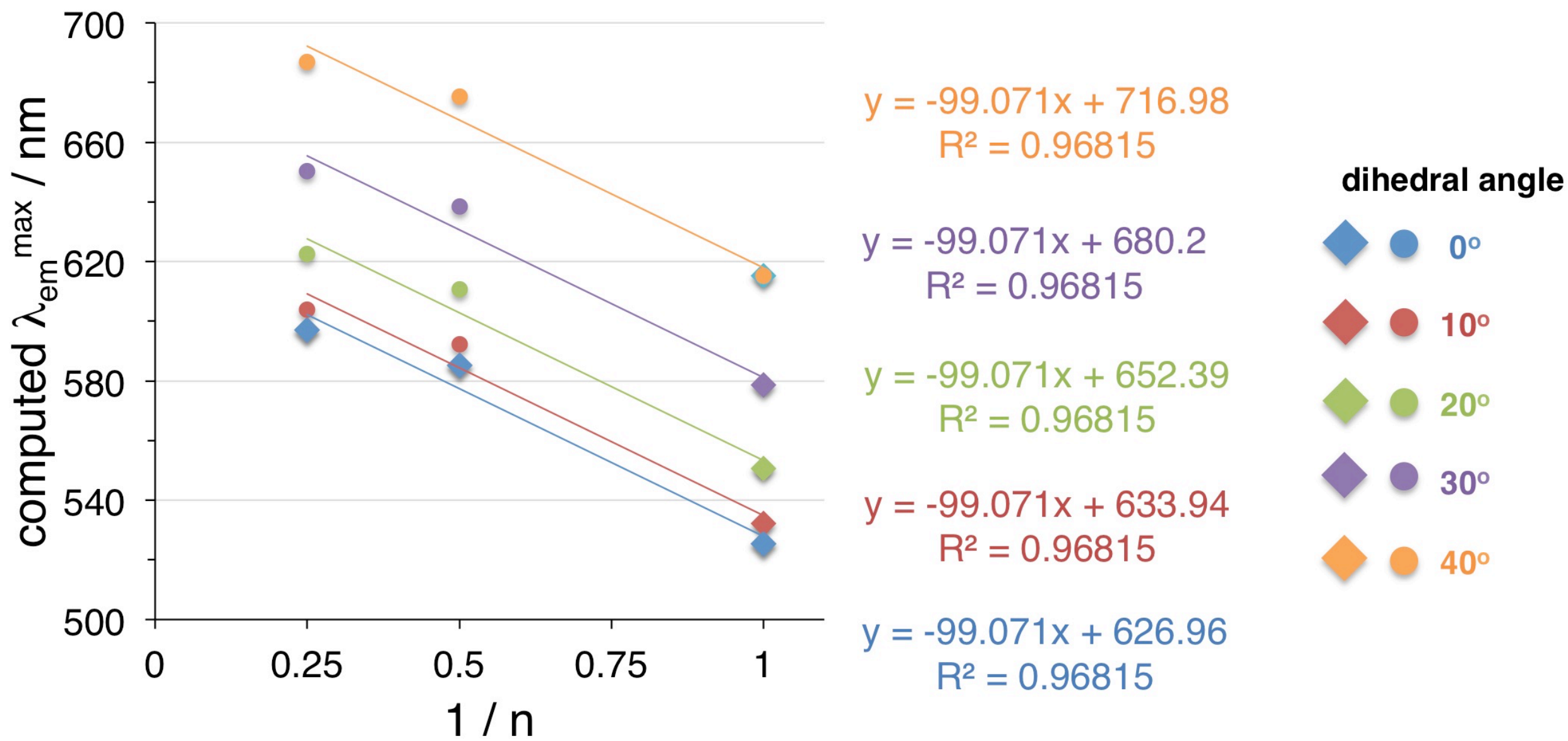


**Figure S22**. Predicted emission of aggregates of **1'** as a function of pyrrole dihedral angle.

n = aggregate (n = 1 – monomer; n = 2 – dimer; n = 4 – tetramer).

*Diamonds*: computed values from optimized structures of monomer (with dihedral angles of 0° (blue), 10° (red), 20° (green), 30° (purple), and 40°(orange) of the pyrrole unit), dimer and tetramer.
*Circles*: values obtained using additive rationale, i.e., contribution of twisting of the pyrrole unit is added to the base values of dimer or tetramer.

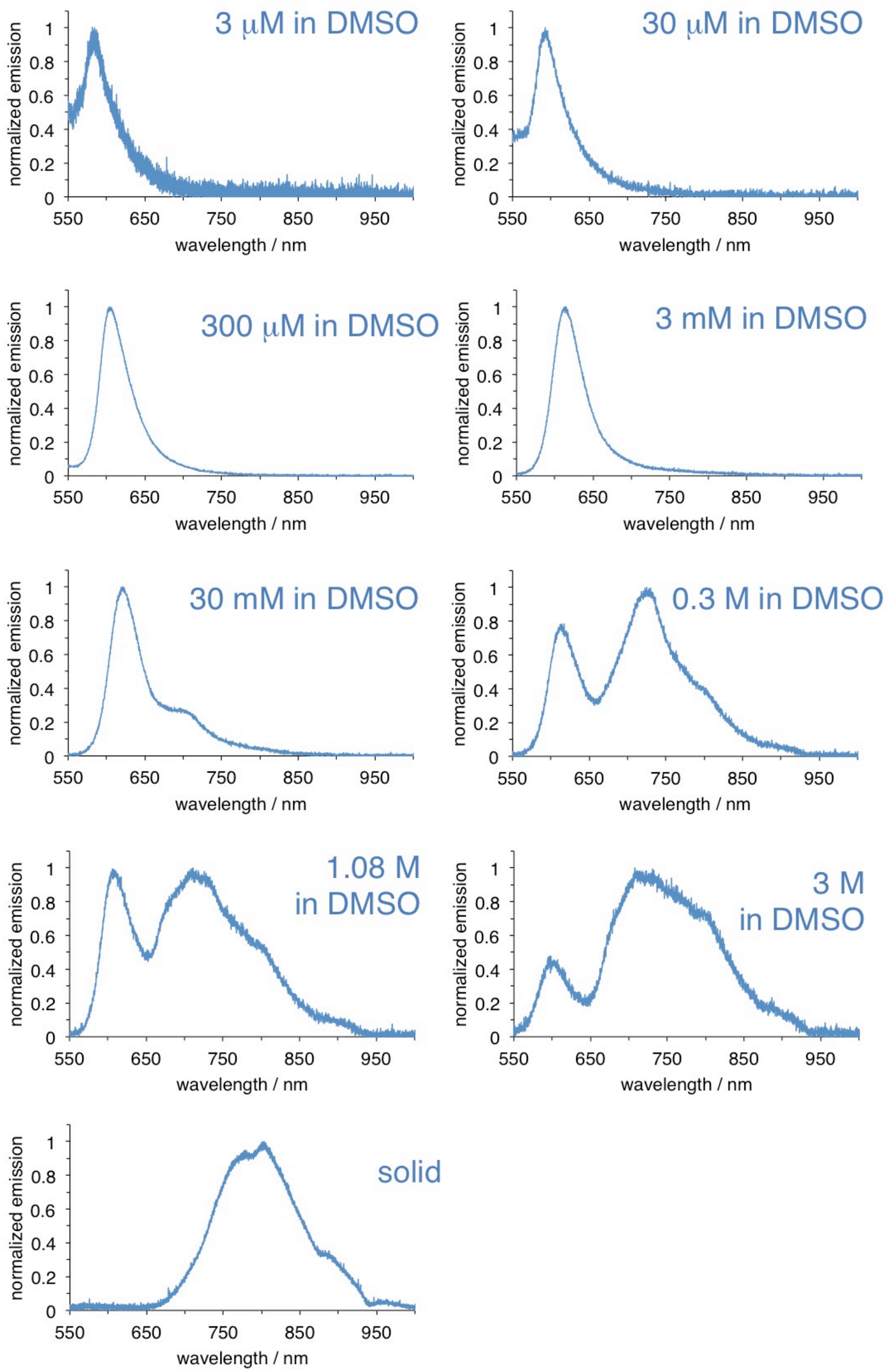


**Figure S23**. Concentration-dependent emission spectra of **1** in DMSO.

Conditions: λex = 485 nm. Concentration of **1** in DMSO is indicated on each spectrum. Heterogeneous mixtures: suspension of **1** in DMSO or **1** "wetted" with DMSO, at concentrations of **1** being 0.3 M and above.

A

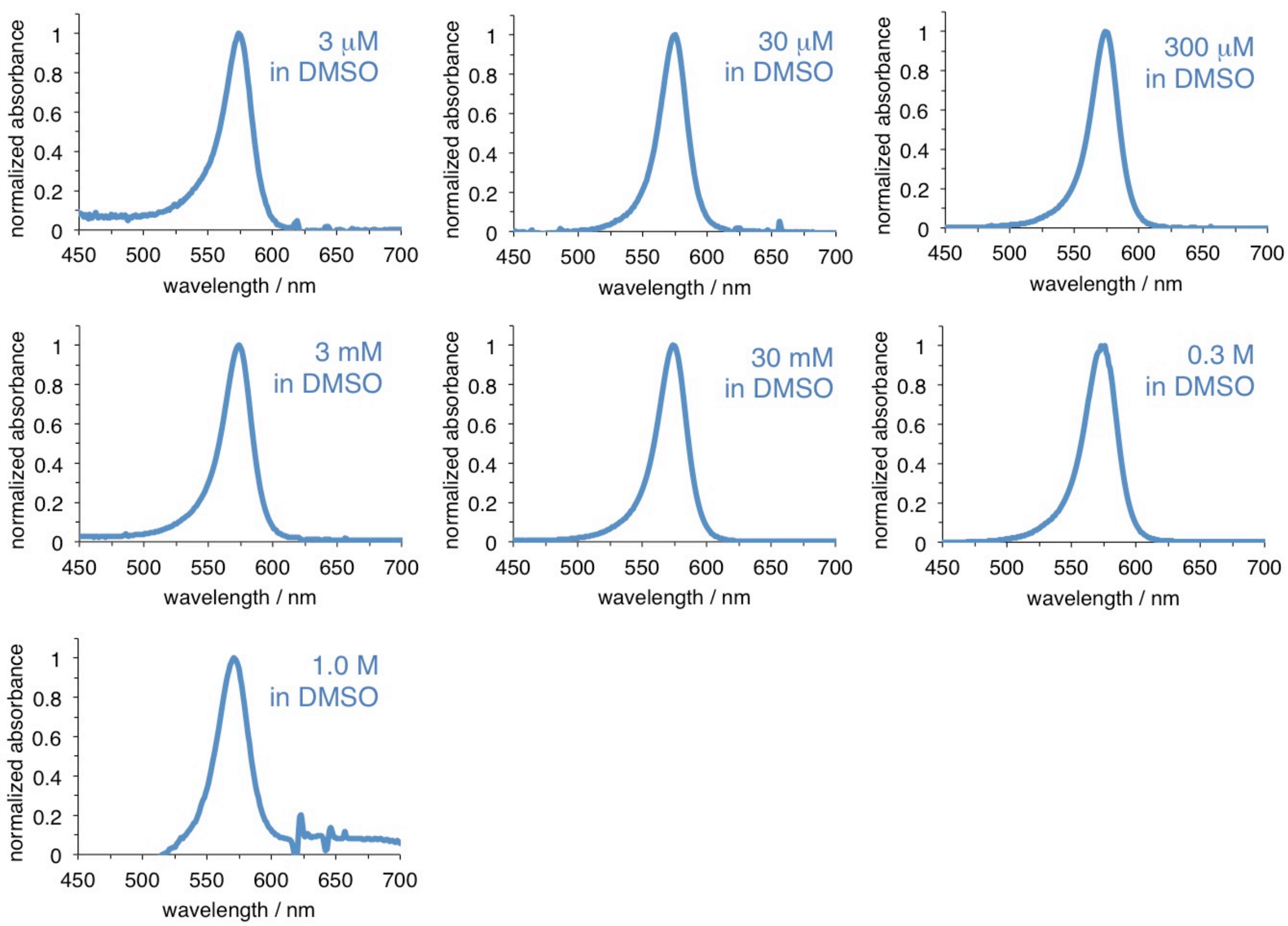


B

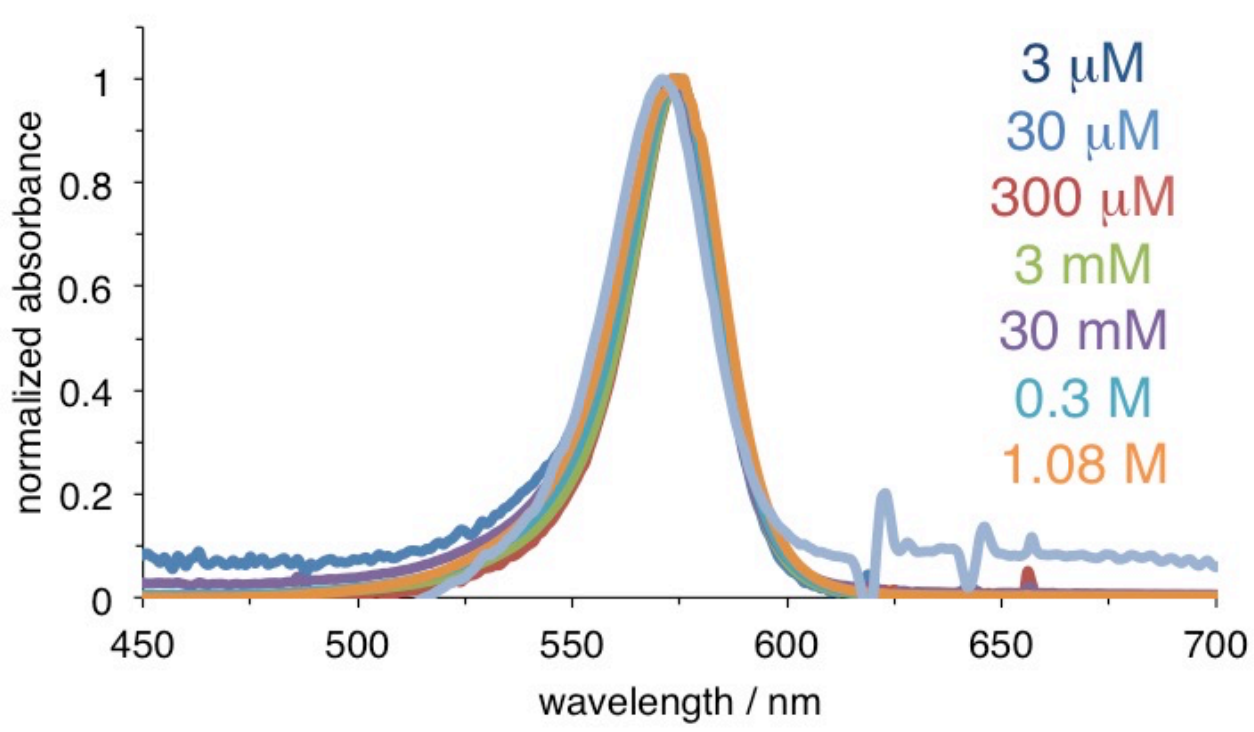


C

| [1] / mM | $\lambda_{ab}^{max}$ / nm |
|---|---|
| 0.003 | 574 |
| 0.03 | 575 |
| 0.3 | 574 |
| 3 | 574 |
| 30 | 574 |
| 300 | 576 |
| 1000 | 571 |

**Figure S24**. Concentration-dependent absorption spectra of **1**. **A**: absorption spectra of **1** at various concentrations in DMSO. Heterogeneous mixtures: suspension of **1** in DMSO or **1** "wetted" with DMSO, at concentrations of **1** being 0.3 M and above. **B**: overlaid absorption spectra as a function of concentration; **C**: Table of absorption maximum ($\lambda_{ab}^{max}$) as a function of concentration of dye **1**.

Conditions: 3 μM sample was acquired in 0.1 cm quartz cuvette; 30 μM and 300 μM samples was acquired using demountable quartz cell with 0.01 cm pathlength. Sample with concentrations in 3.0 mM – 1.0 M range were acquired using glass coverslips (small amount of sample was placed in the middle of the cover slip, followed by placing another coverslip on top). Absorption spectra of higher concentration samples (i.e., heterogeneous mixtures: suspension of **1** in DMSO or **1** "wetted" with DMSO, at concentrations of **1** above 1.0 M) as well as the solid **1**, were not possible to obtain due to excessive scattering.

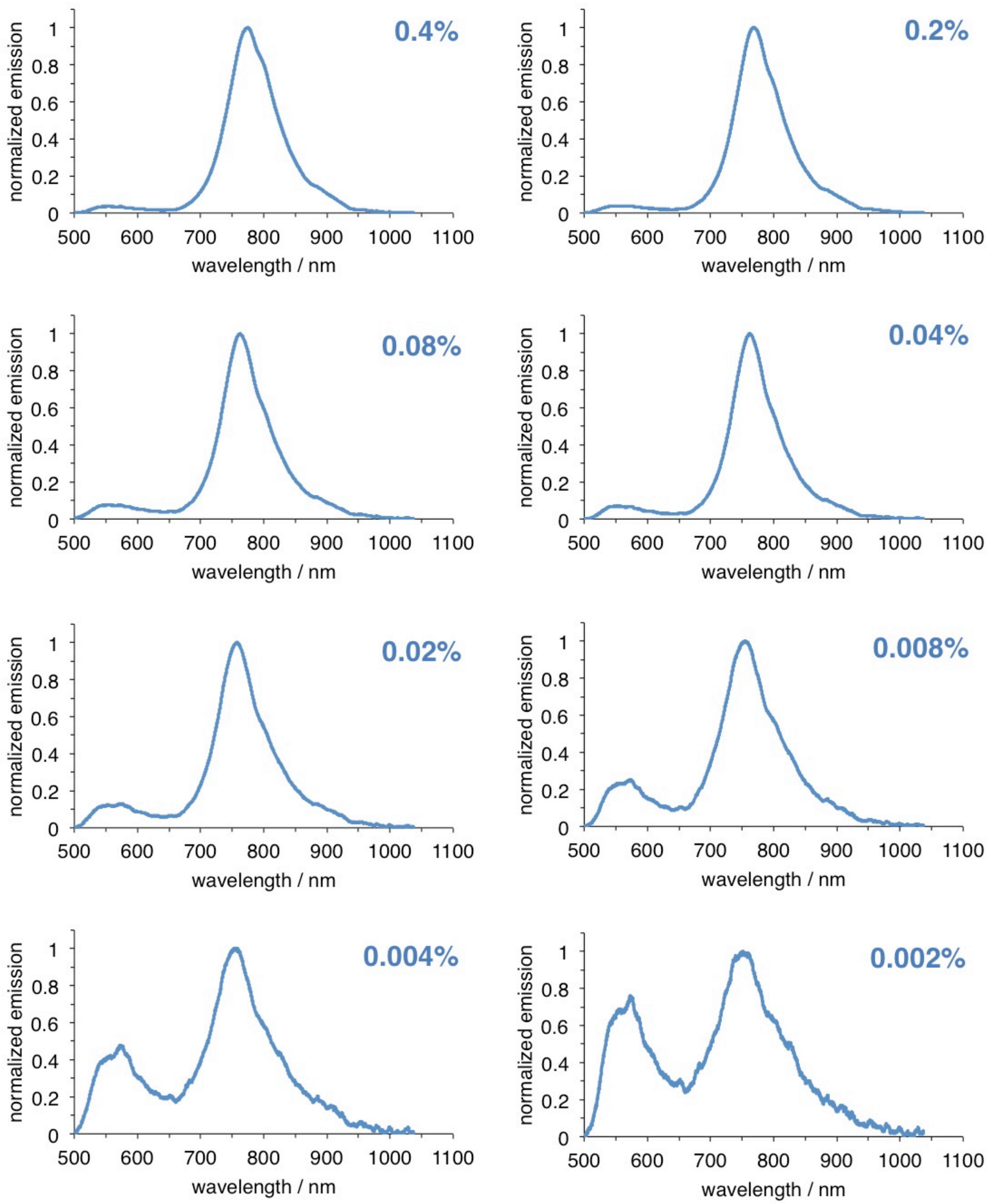


**Figure S25**. Concentration-dependent emission spectra of **1** in KBr.
Conditions: $\lambda$ex = 485 nm. Concentration of **1** in KBr (%, wt/wt) is indicated on each spectrum.

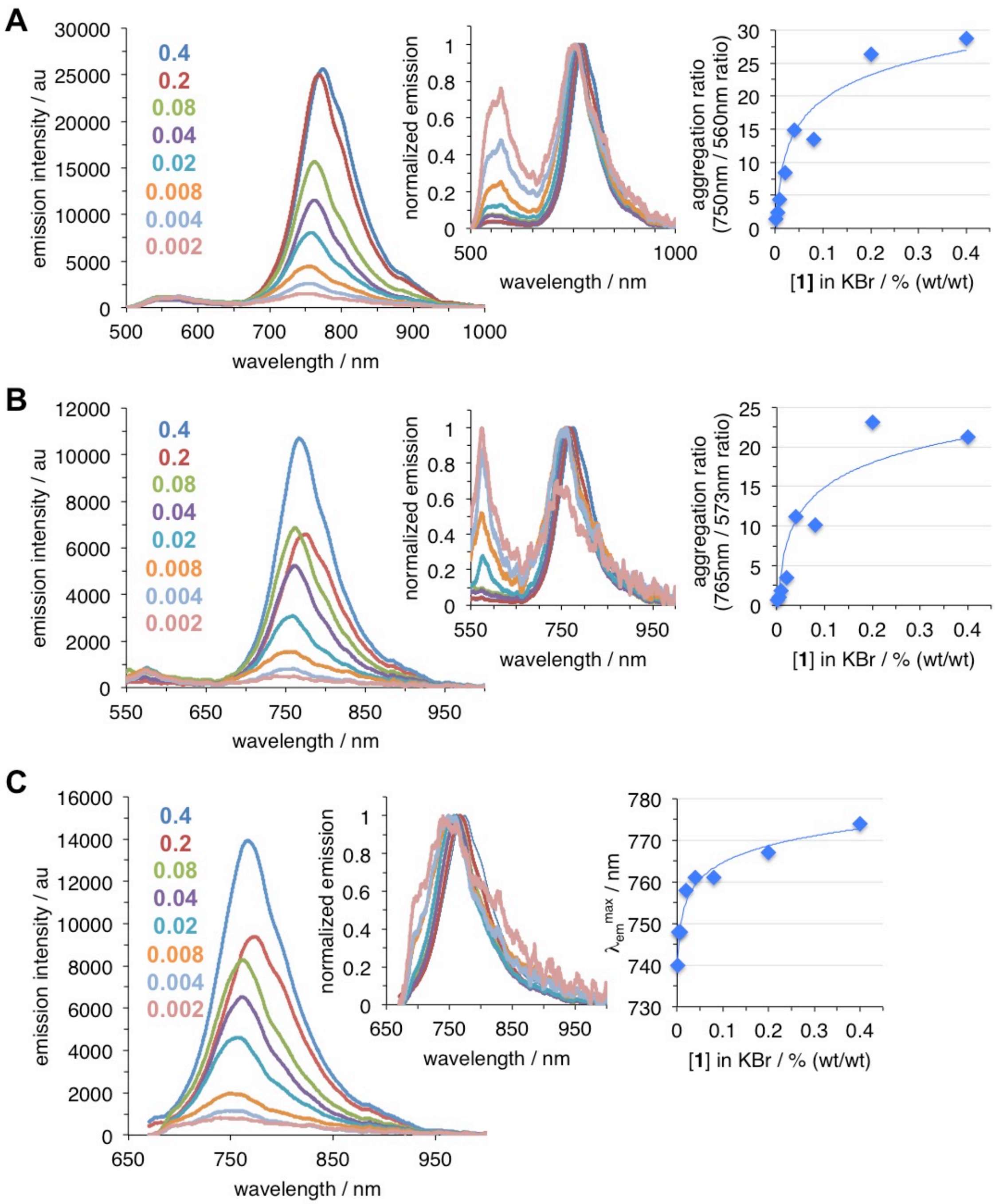


**Figure S26**. Excitation dependent emission spectra of **1** in KBr. Concentrations of **1** in KBr (0.4, 0.2, 0.08, 0.04, 0.02, 0.008, 0.004, 0.002 % wt/wt) are given next to the spectra.

**A**: λex = 485nm; integration time: 1000ms. Insets: normalized emission spectra (left), effect of concentration of **1** on the relative intensities of the emission maxima peaks (right).

**B**: λex = 530nm; integration time: 1000ms. Insets: normalized emission spectra (left), effect of concentration of **1** on the relative intensities of the emission maxima peaks(right).

**C**: λex = 655nm; integration time: 1000ms. Insets: normalized emission spectra (left), effect of concentration of **1** on the emission maxima.

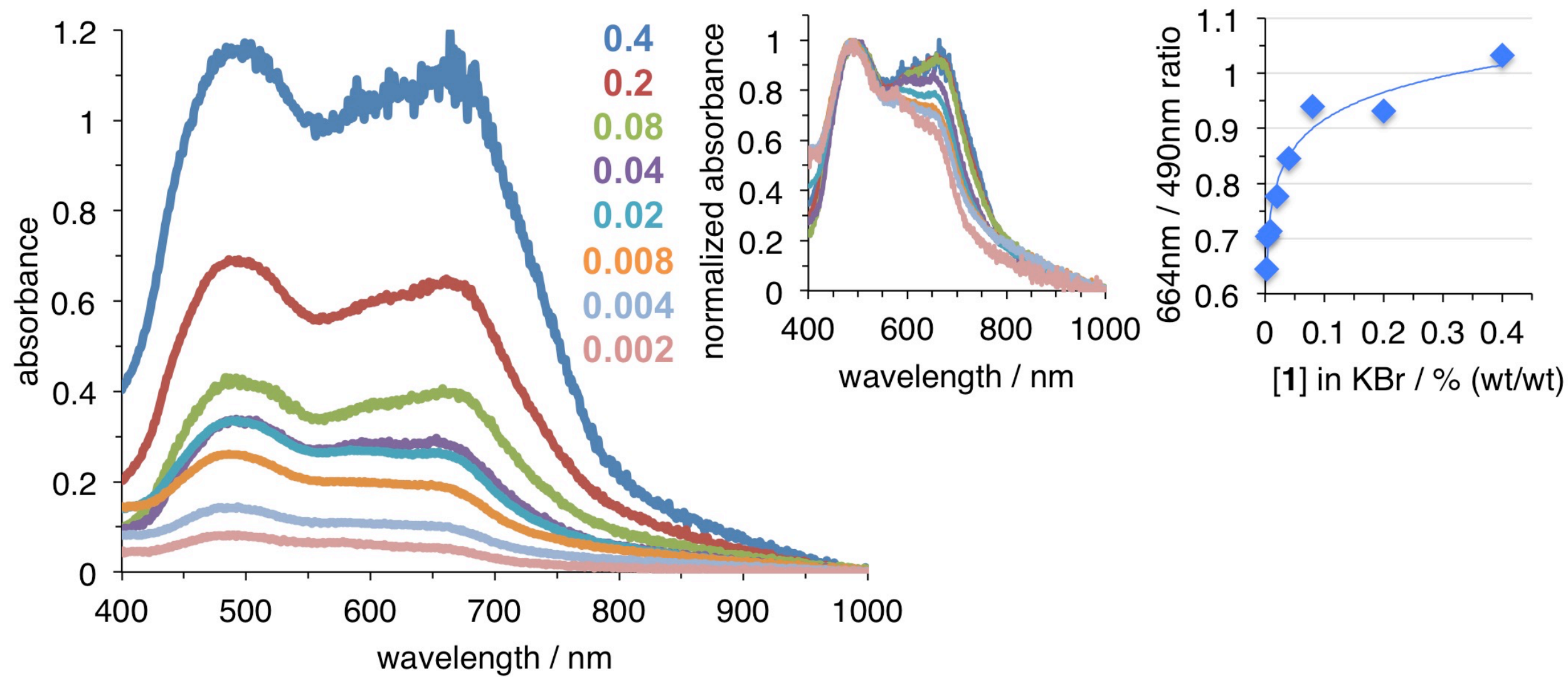


**Figure S27**. Concentration dependent absorbance spectra of **1** in KBr.
Concentrations of **1** in KBr (0.4, 0.2, 0.08, 0.04, 0.02, 0.008, 0.004, 0.002 % wt/wt) are given next to the spectra. Insets: normalized absorbance (left), effect of concentration of **1** on the ratio of the absorbance intensities at 664nm and 490nm.

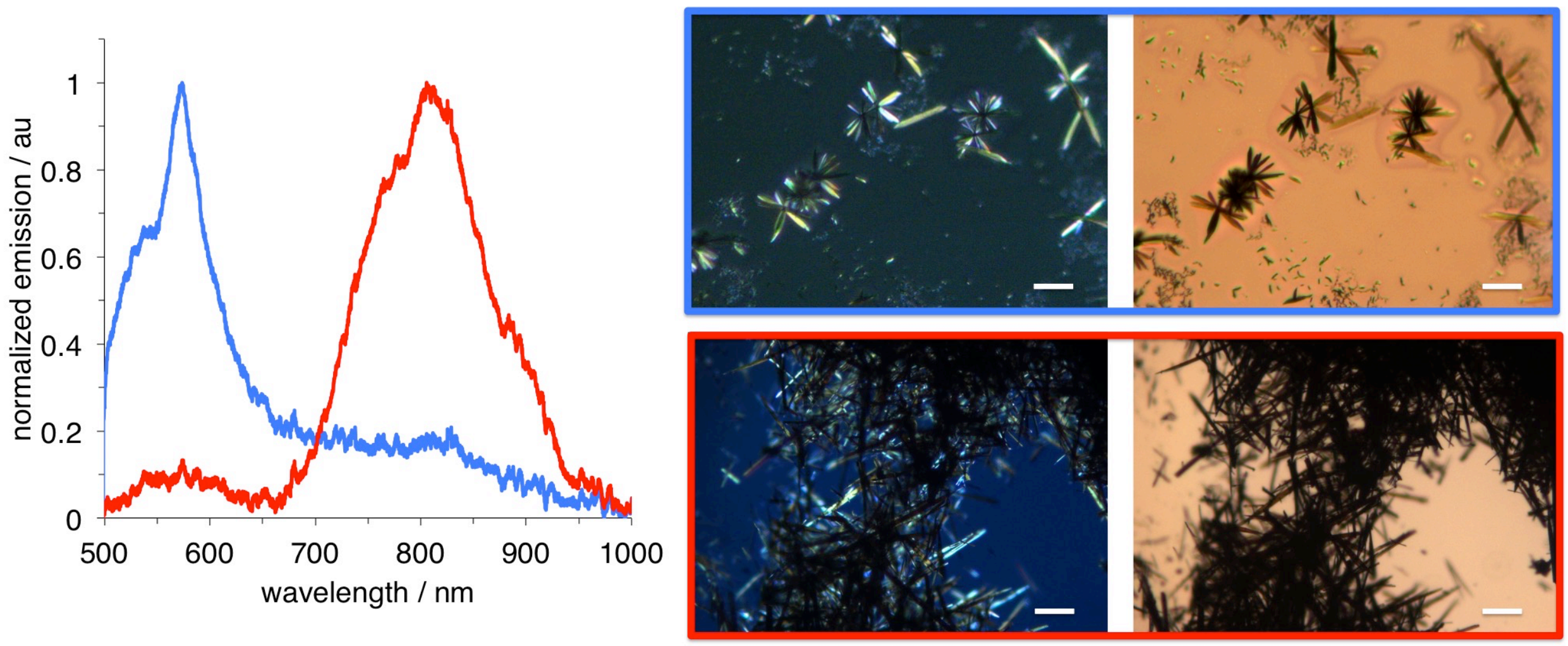


**Figure S28**. Normalized emission spectra of dye **1** and the respective optical microscopy images.

Disaggregated dye – blue trace (emission spectrum) and blue box (images); left – polarized light exposure, and right – direct field exposure.
Aggregated dye – red trace (emission spectrum) and red box (images): left – polarized light exposure, and right – direct field exposure.

Conditions for emission spectra: $\lambda_{ex}$ = 485 nm, front face (bifurcated fiber), laser diode; integration time 1000 ms.

Scale bar in all optical microscopy images is 20 $\mu$m.

**Table S1**. Solvent parameters and NH– and $CH_3$–groups resonances in the respective solvents.

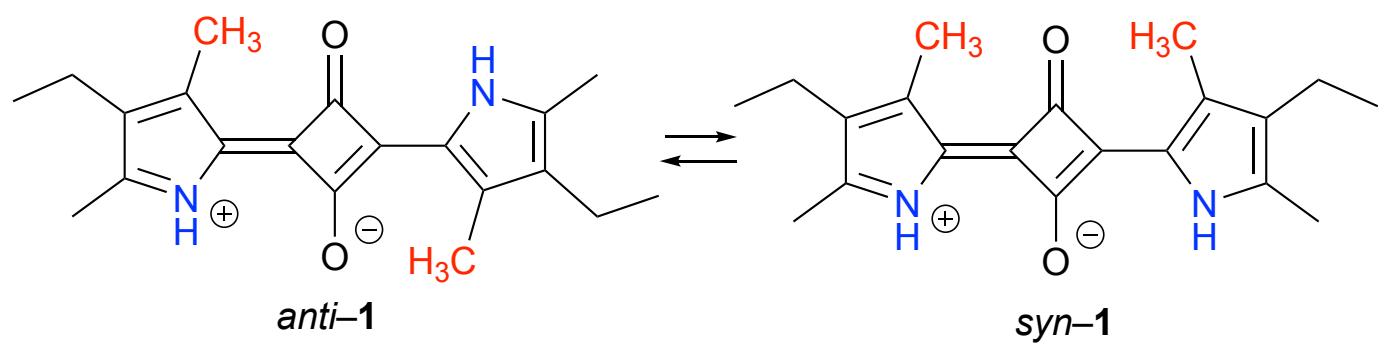


| Solvent | $E_T(30)$ / kcal mol$^{-1}$ [a] | $\pi^{*}$ [b] | $\varepsilon$ [c] | NH / ppm [d] | $CH_3$ / ppm [d] |
|---|---|---|---|---|---|
| $CH_3$-$C_6H_5$ | 33.9 | 0.535 | 2.379[e] | 10.12 / 9.90 | 2.77 / 2.74 |
| $CHCl_3$ | 39.1 | 0.760 | 4.806[f] | 10.03 / 9.83 | 2.60 / 2.56 |
| $NO_2$-$C_6H_5$ | 41.2 | 1.029 | 34.82[e] | 10.05 / 9.94 | 2.38 / 2.28 |
| DMSO | 45.1 | 1.000 | 47.2[g] | 11.25 | 2.54 |
| $CH_3OH$ | 55.4 | 0.586 | 32.63[e] | 8.54 | 2.57 |
| $CH_3CN$ | 45.6 | 0.713 | 37.5[f] | 10.02 | 2.53 |
| $CH_3C(O)CH_3$ | 42.2 | 0.683 | 20.70[e] | 10.46 | 2.57 |
| $NC_5H_5$ | 40.5 | 0.867 | 12.3[e] | 11.62 | 2.81 |

[a] – data taken from ref. S13.
[b] – data taken from ref. S14.
[c] – data taken from ref. S15.
[d] – deuterated version of the solvents was used to record $^1$H NMR spectra of **1**. See Figure S4 and copies of full $^1$H NMR spectra of **1** in various solvents for details.
[e] – data given at 25 $^{o}$C.
[f] – data given at 20 $^{o}$C.
[g] – data taken from ref. S16.

**Table S2**. Computed energies and chemical shifts of NH-resonances of *anti*- and *syn*-conformers of dye **1'** using various solvent models.[a]

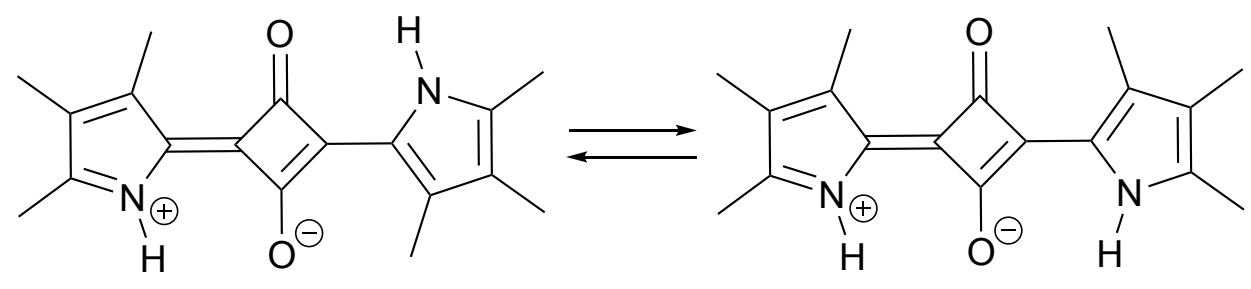


| Solvent model | ΔG (kJ/mol) | | Shielding relative to TMS (ppm) | |
|---|---|---|---|---|
| | | | computed | Δ ($\Delta_{exp}$)[b] |
| | ***anti-NH*** | ***syn-NH*** | ***anti-NH / syn-NH*** | |
| $CDCl_3$ medium | 0 | 3.676 | 9.69 / 9.47 | 0.22 (0.20) |
| DMSO medium | 0 | 1.575 | 9.67 / 9.50 | 0.17 (0.00) |
| $CDCl_3$ medium + 1 coordinated $CDCl_3$ molecule | 0 | 4.988 | 9.63 / 9.43 | 0.20 (0.20) |
| DMSO medium + 1 coordinated DMSO molecule | 0 | 5.514 | 11.29 / 11.30 | 0.01 (0.00) |
| MeOH medium + 1 coordinated MeOH molecule | 0 | – 1.100 | 10.79 / 10.76 | 0.03 (0.00) |
| $NO_2$-Ph medium + 1 coordinated $NO_2$-Ph molecule | 0 | 0.525 | 9.33 / 8.75 | 0.58 (0.11) |
| Acetone medium + 1 coordinated acetone molecule | 0 | 2.100 | 10.06 / 9.98 | 0.08 (0.00) |

[a] – Ref S4.
[b] – Δ = δ (anti-NH) – δ (syn-NH). Experimental Δ values are given in parentheses for comparison. See Figure S4 and $^1$H NMRs for actual spectra/δ values.

**Table S3**. Photophysical properties of dye **1** in various organic solvents.[a]

| **solvent** | **$\lambda_{ab}^{max}$ / nm** | **$\lambda_{em}^{max}$ / nm** | **QY / %[e]** | **<τ>$_{int}$ & <τ>$_{amp}$ / ns** |
|---|---|---|---|---|
| | this work [ref.S9] | this work [ref.S9] | this work [ref.S9[f]] | |
| DMSO | 573 [–[b]] | 586 [–[b]] | 1.9 [–[b]] | 0.189 & 0.048 |
| toluene | 569 [564] | 578 [583] | 6.73 [0.06] | 0.173 & 0.173 |
| hexane | 563 [563] | 569 [574] | 40.8 [0.49] | 1.525 & 1.348 |
| chloroform | 564 [564] | 573 [578] | 3.7 [0.07] | 0.134 & 0.112 |
| dioxane | 564 [–[b]] | 573 [–[b]] | 1.7 [–[b]] | 0.275 & 0.150 |
| methanol | 559 [559] | 571 [574] | 0.5 [<0.01] | 0.183 & 0.043 |
| water | 487[c] [–[b]] | –[d] [–[b]] | –[d] | –[d] |

[a] – conditions: [**dye 1**] = 1 μM; see Figures S6 – S9 for spectra. QY = quantum yield; <τ>$_{int}$ and <τ>$_{amp}$ are intensity average lifetime and amplitude average lifetime, respectively. See ref. S17 for the description of and the differences between <τ>$_{int}$ and <τ>$_{amp}$.
[b] – not reported.
[c] – broad spectrum.
[d] – non-emissive.
[e] – Rhodamine 101 in ethanol was used as the standard.
[f] – absolute values reported using integrated sphere method.

**Table S4**. Solvent properties.

| **solvent** | **$E_T(30)$[a] / kcal mol$^{-1}$** | **π*[b]** | **ε[c]** | **viscosity / cP** |
|---|---|---|---|---|
| DMSO | 45.1 | 1.000 | 47.2[d] | 2.0[g] |
| toluene | 33.9 | 0.535 | 2.379[e] | 0.6[h] |
| hexane | 31.0 | –0.081 | 1.89[f] | 0.3[h] |
| chloroform | 39.1 | 0.760 | 4.806[f] | 0.6[g] |
| dioxane | 36.0 | 0.553 | 2.209[e] | 1.4[g] |
| methanol | 55.4 | 0.586 | 32.63[e] | 0.7[g] |
| water | 63.1 | 1.090 | 78.54[e] | 1.0[g] |

[a] – data taken from ref. S13.
[b] – data taken from ref. S14.
[c] – data taken from ref. S15.
[d] – data taken from ref. S16.
[e] – data given at 25 °C.
[f] – data given at 20 °C.
[g] – data taken from ref. S18.
[h] – data taken from ref. S19.

**Table S5**. Average fluorescence lifetimes of dye **1** in solvents of various viscosities.

| solvent | solvent viscosity / cP[a] | $<\tau>_{INT}$ of 1 / ns | $<\tau>_{AMP}$ of 1 / ns |
|---|---|---|---|
| methanol | 0.7 | 0.183 | 0.043 |
| glycerol/methanol (3/7)[b] | 4.0 | 0.077 | 0.033 |
| glycerol/methanol (4/6)[b] | 9.0 | 0.141 | 0.051 |
| glycerol/methanol (6/4)[b] | 39 | 0.250 | 0.131 |
| glycerol/methanol (7/3)[b] | 131 | 0.268 | 0.208 |
| glycerol/methanol (8/2)[b] | 239 | 0.520 | 0.418 |
| glycerol/methanol (9/1)[b] | 477 | 0.862 | 0.786 |
| glycerol | 1501 | 1.415 | 1.324 |

[a] – data taken from ref. 18.
[b] – solvent mixtures were prepared in ratios given in parentheses (v/v).

# $^{1}$H NMR SPECTRA OF DYE 1 IN DEUTERATED SOLVENTS

# AND

# 2D NMR SPECTRA OF DYE 1

$^{1}$H NMR of **1** in $CDCl_3$

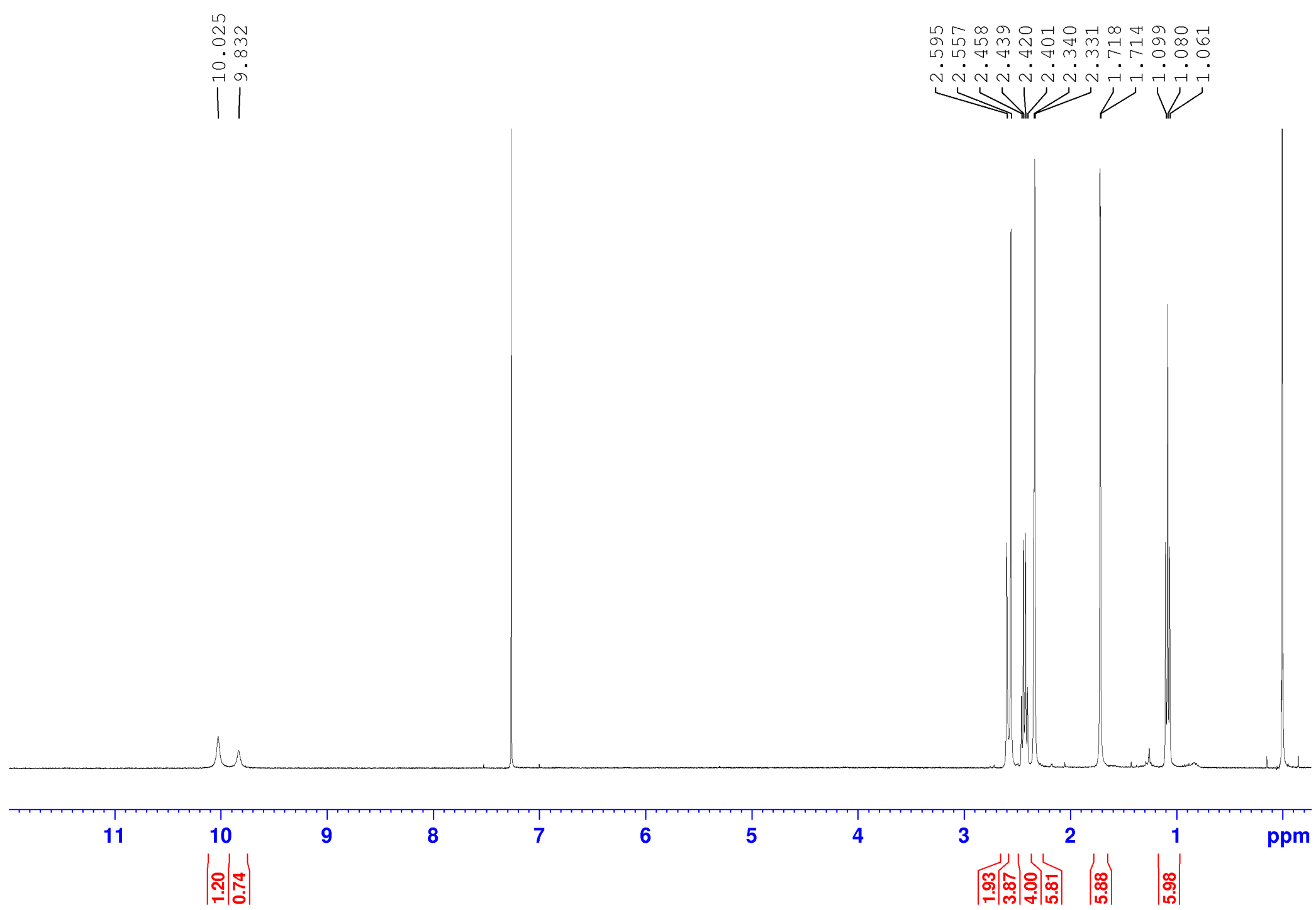


$^{1}$H NMR of **1** in pyridine-$d_5$

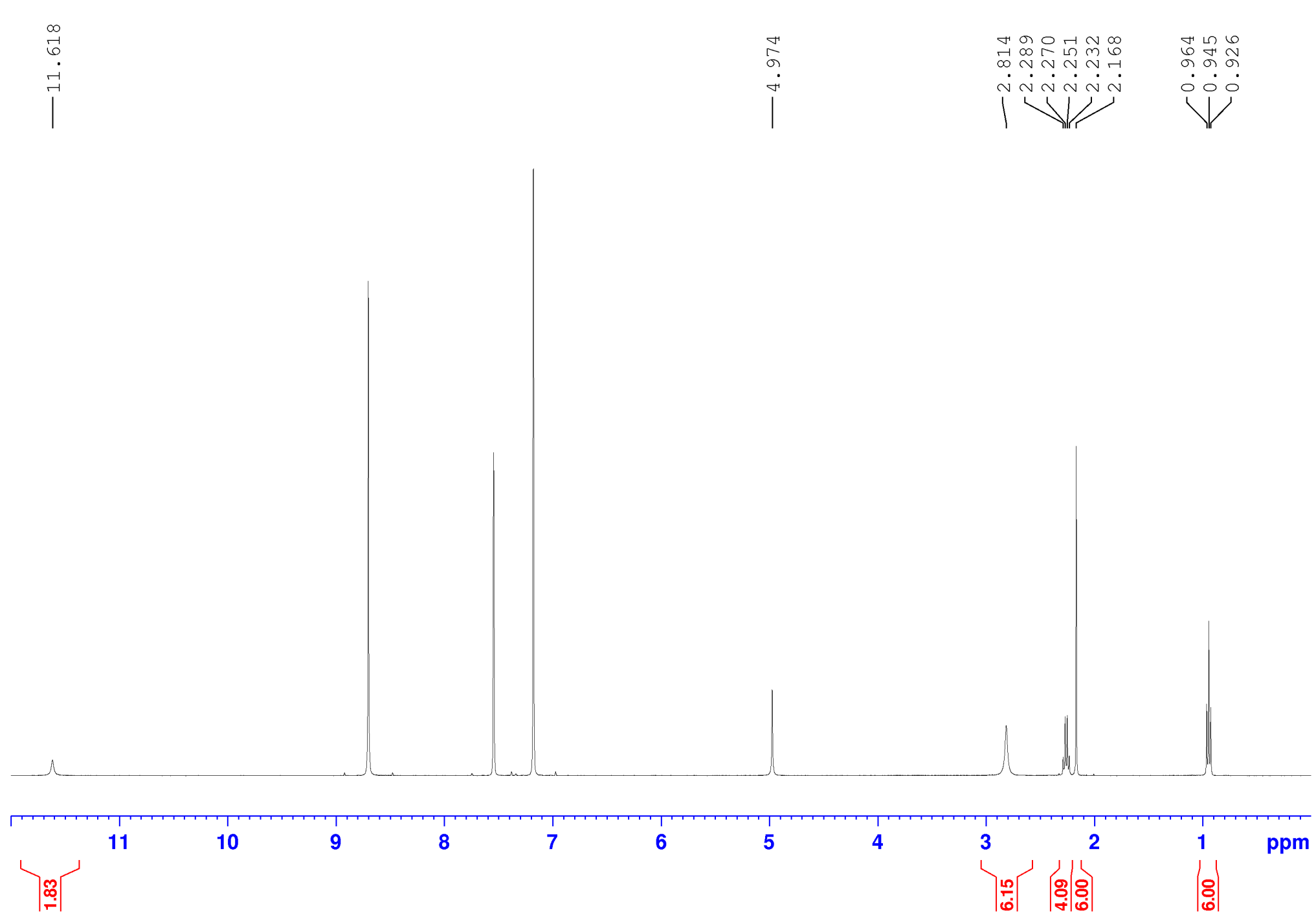

$^{1}$H NMR of **1** in toluene-d$_8$

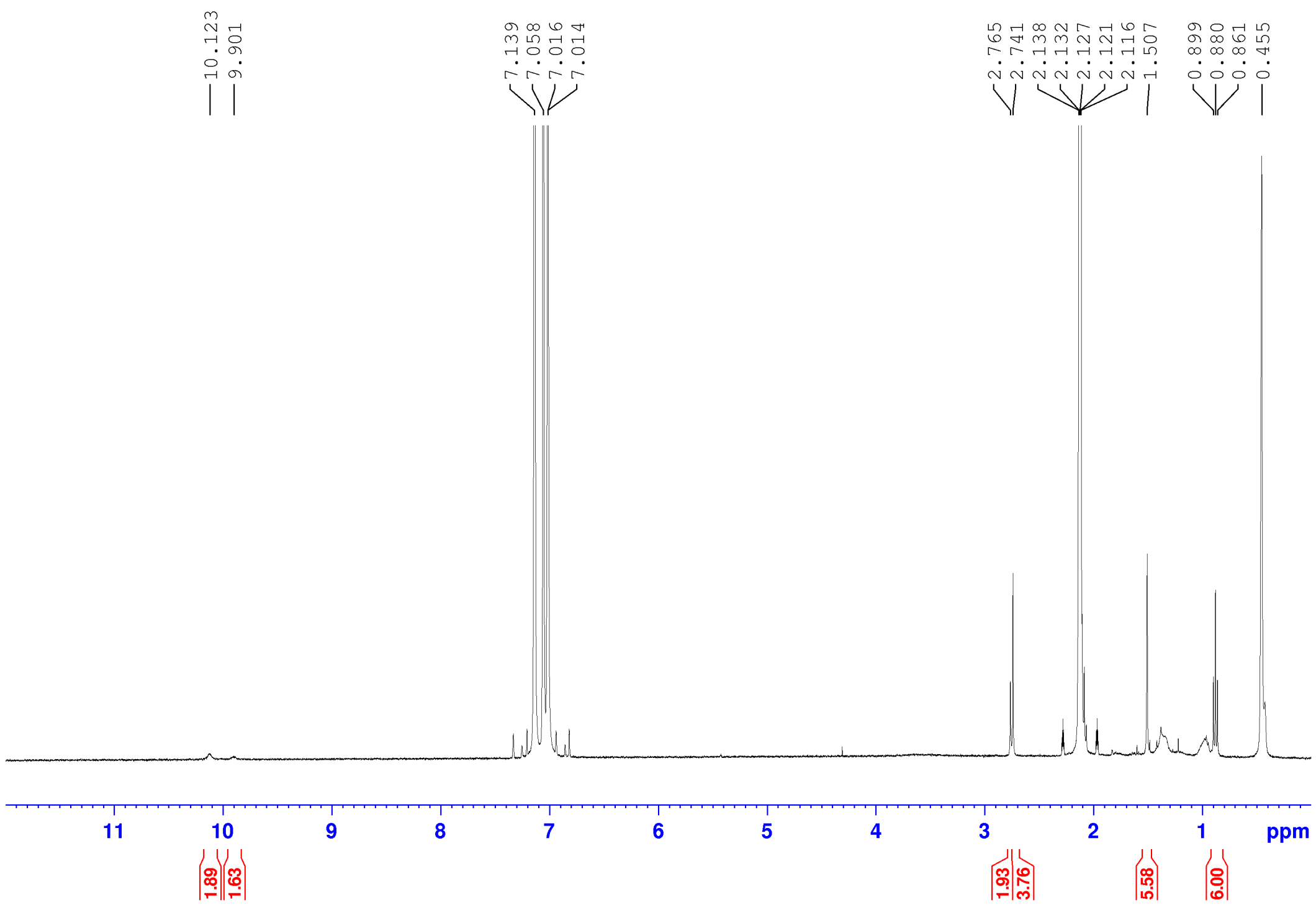


$^{1}$H NMR of **1** in nitrobenzene-d$_5$

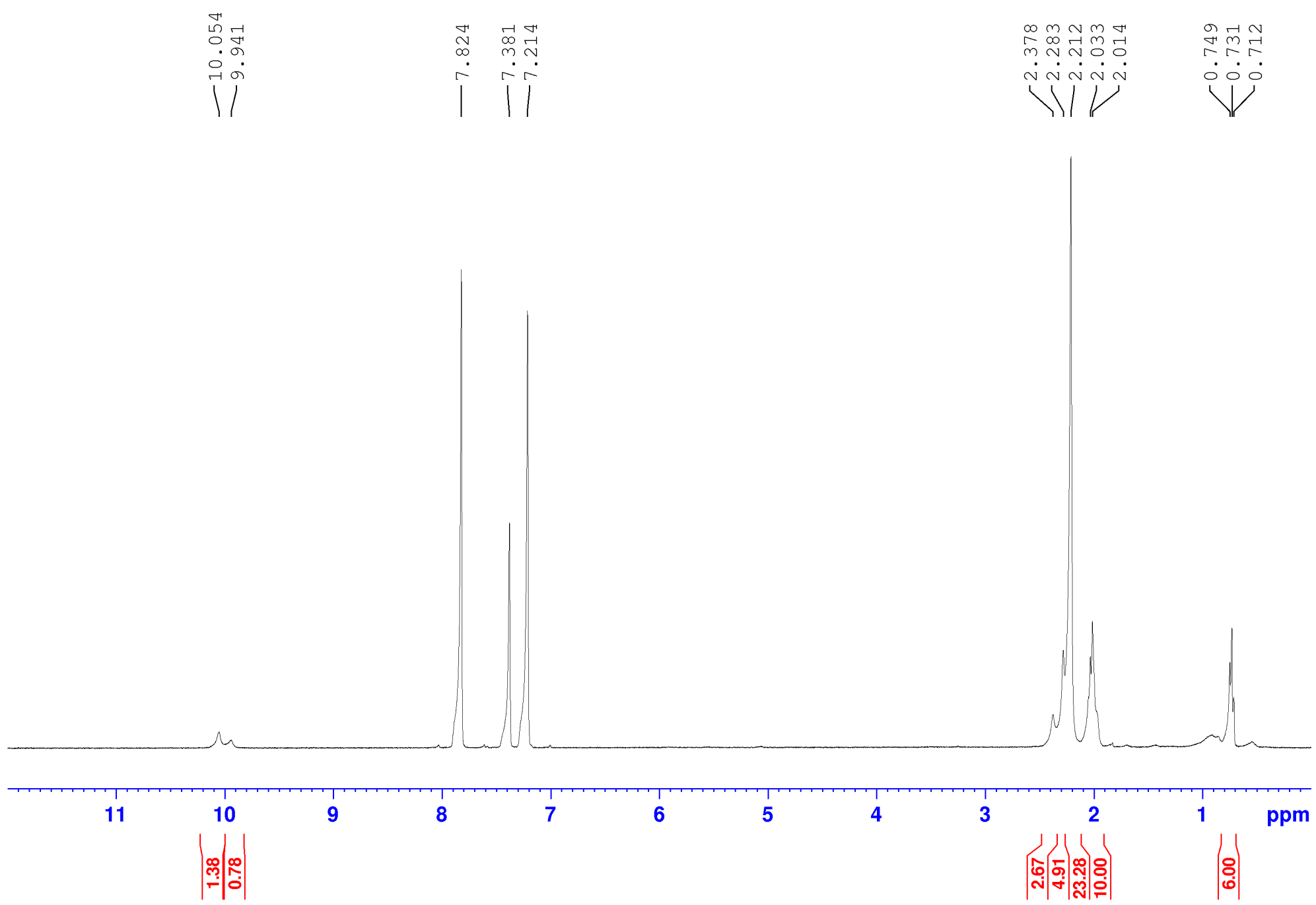

$^{1}$H NMR of **1** in DMSO-d$_6$

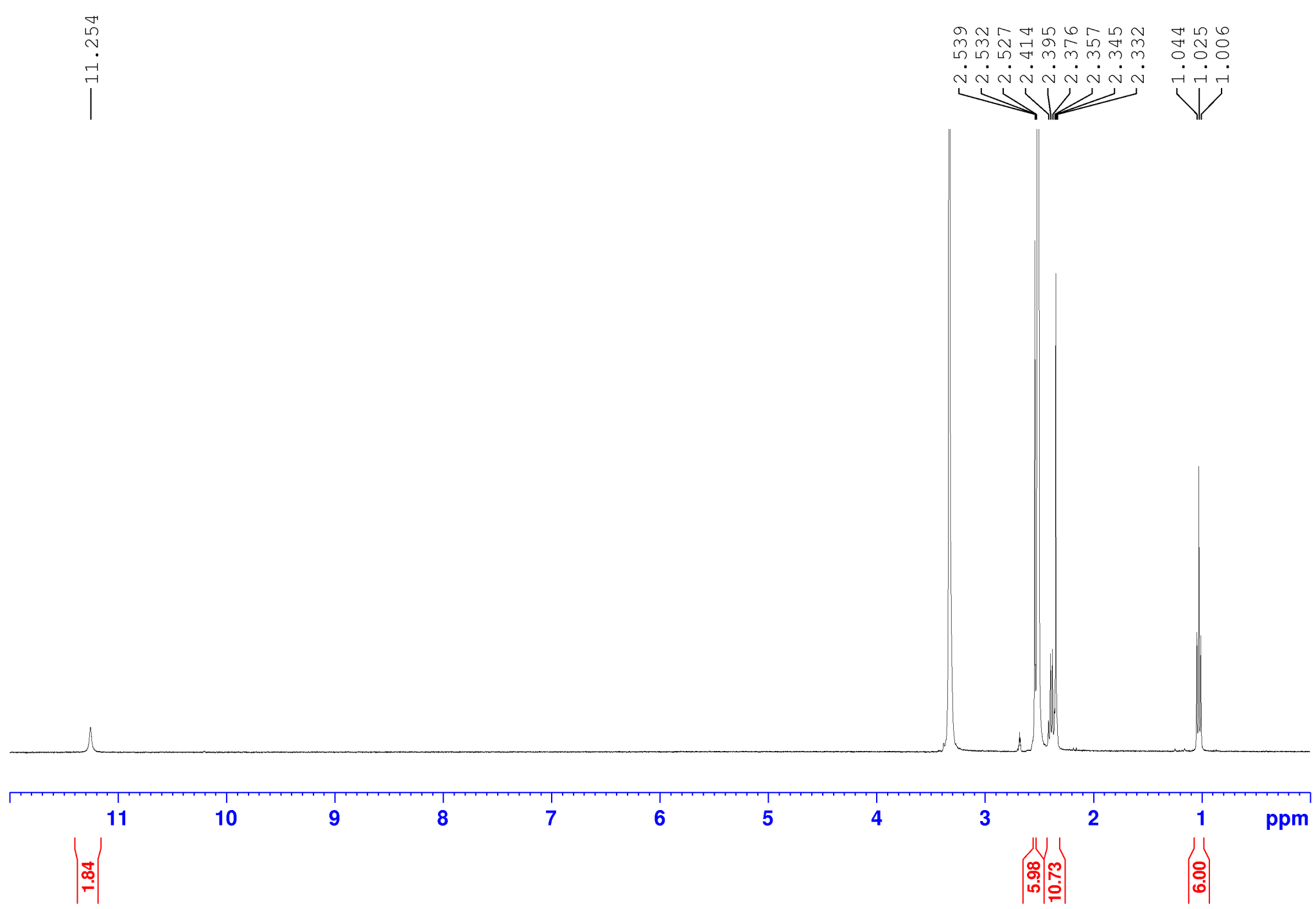


$^{1}$H NMR of **1** in acetone-d$_6$

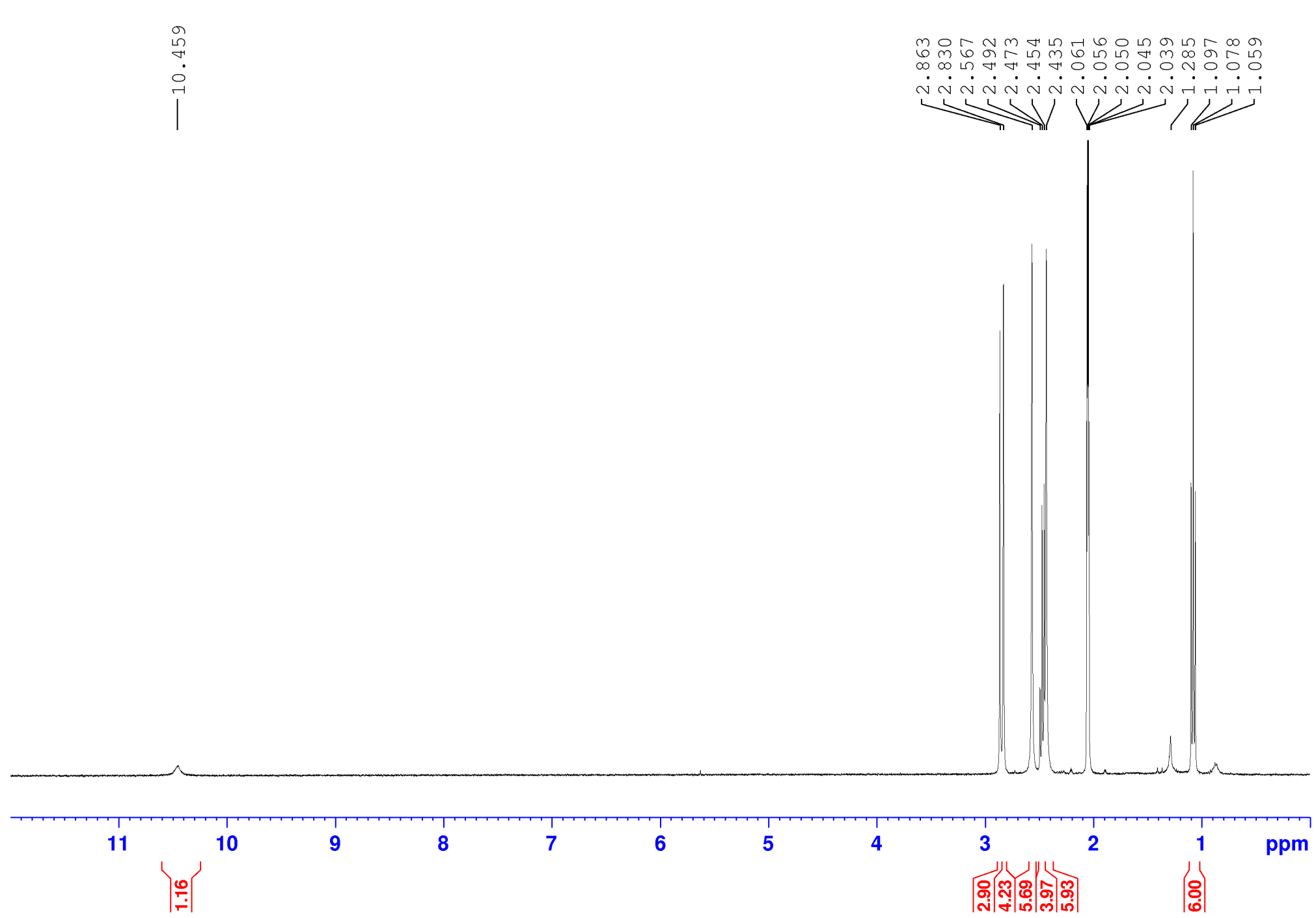

## $^1H$ NMR of **1** in $CD_3OD$

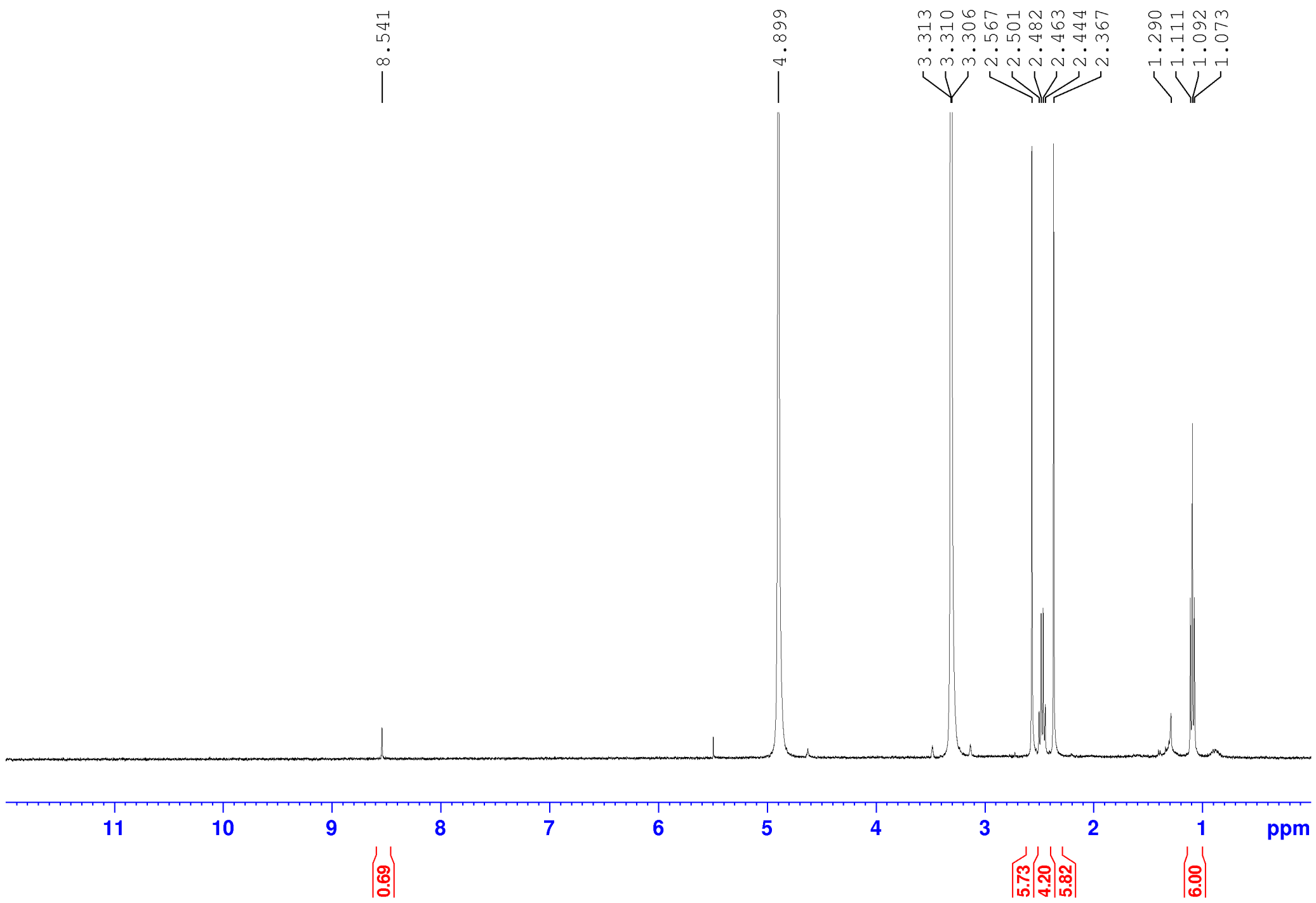


## $^1H$ NMR of **1** in $CD_3CN$

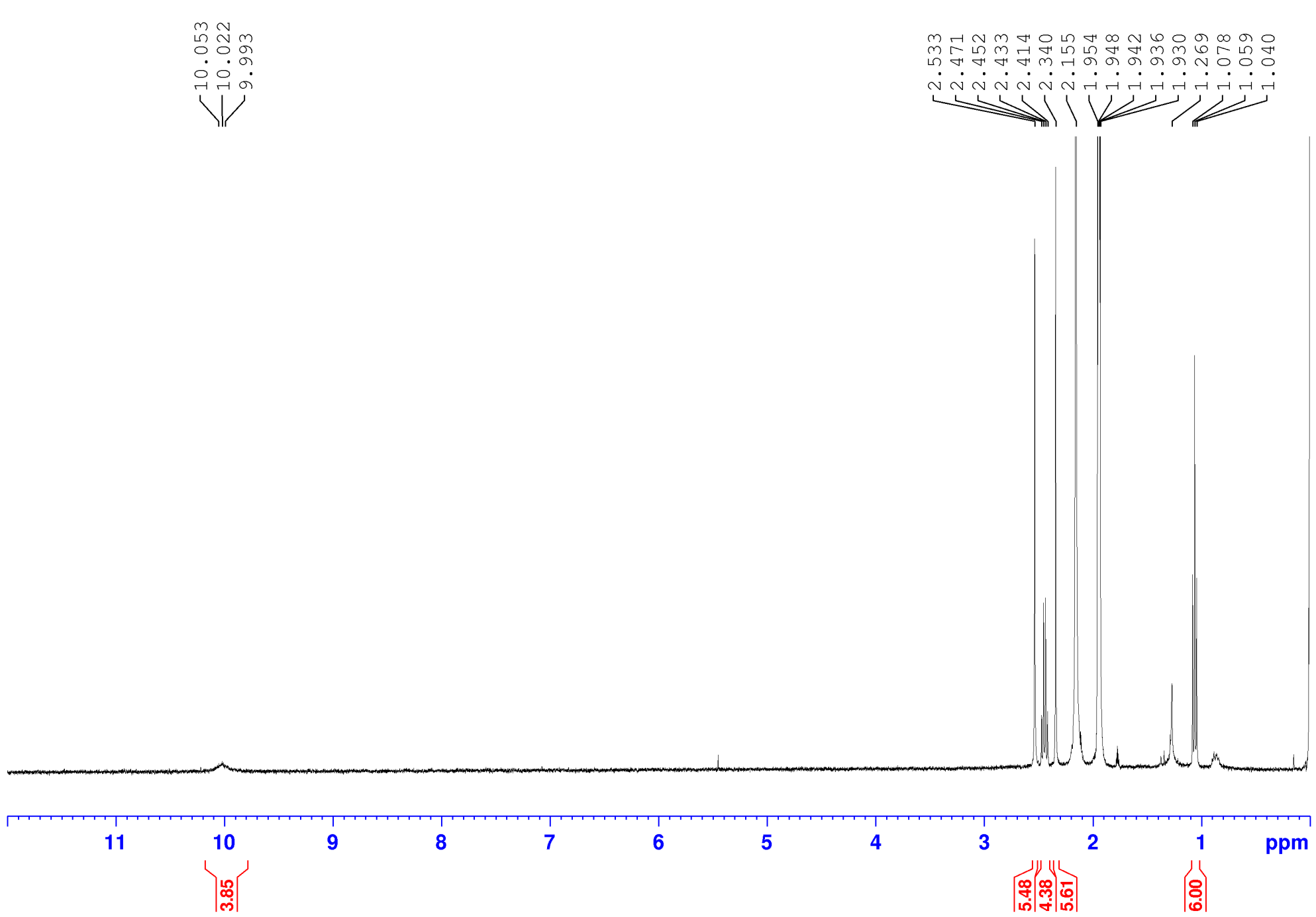

HMBC spectrum of **1** in $CDCl_3$

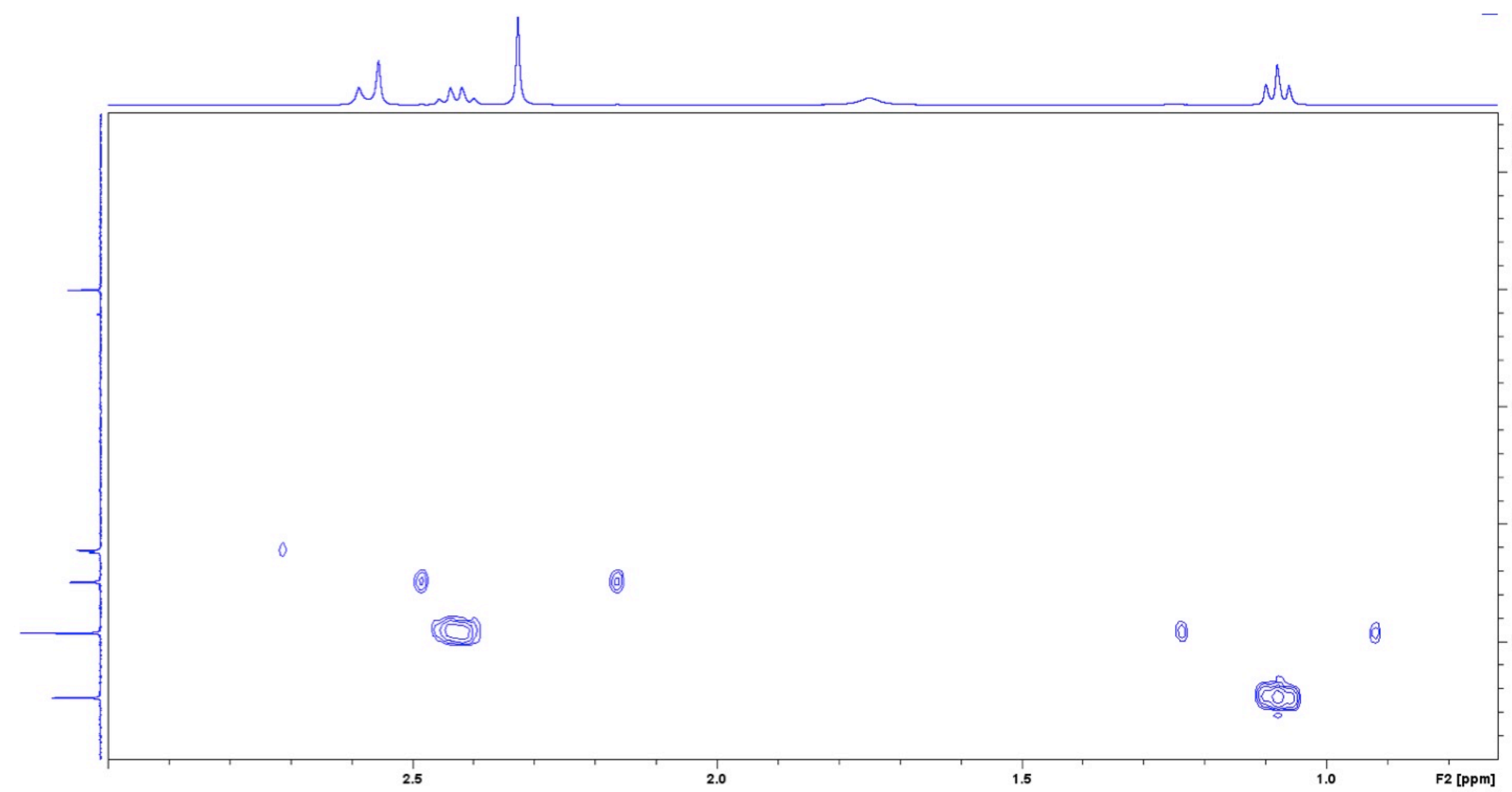


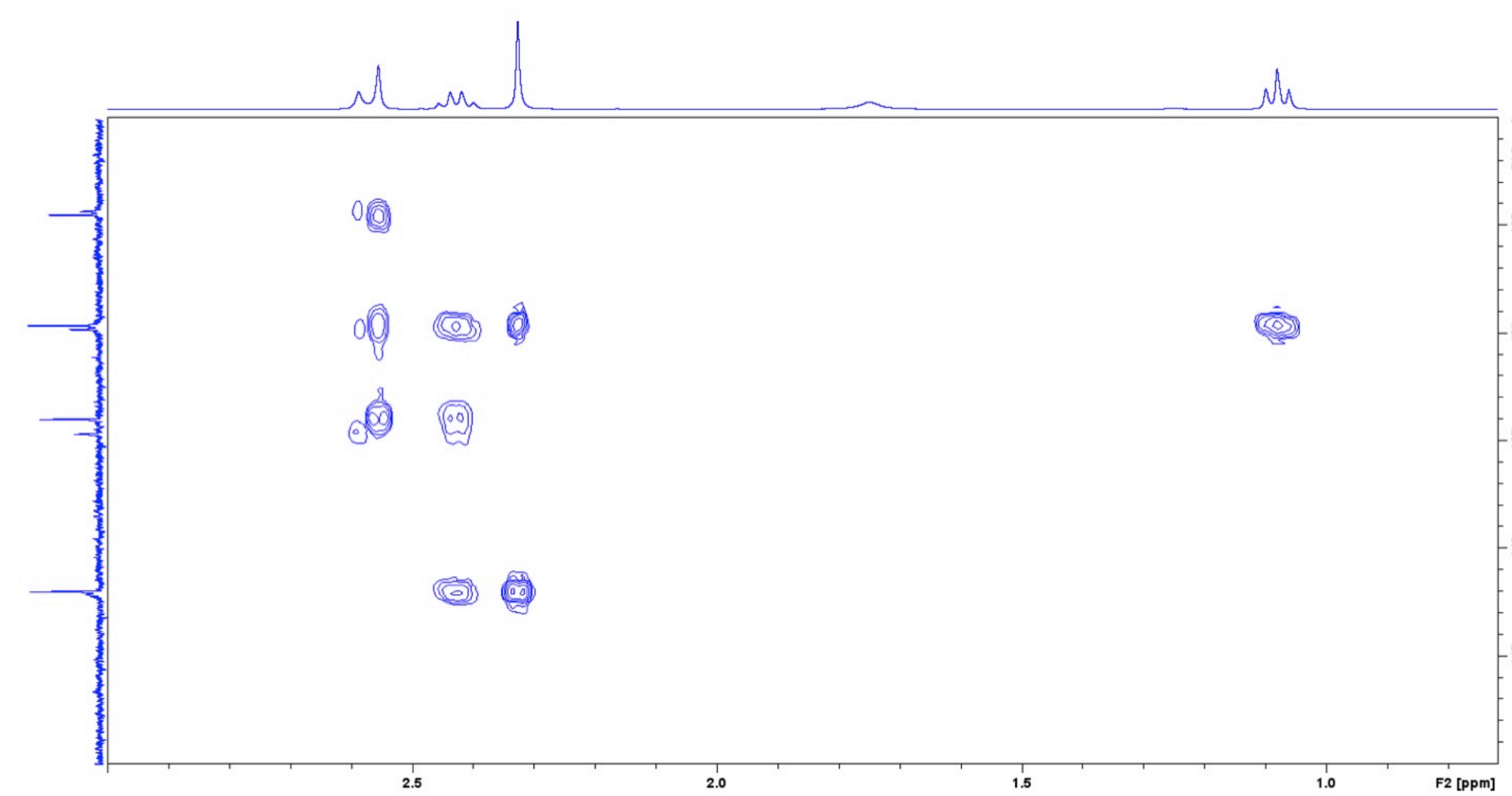

HSQC spectrum of **1** in $CDCl_3$

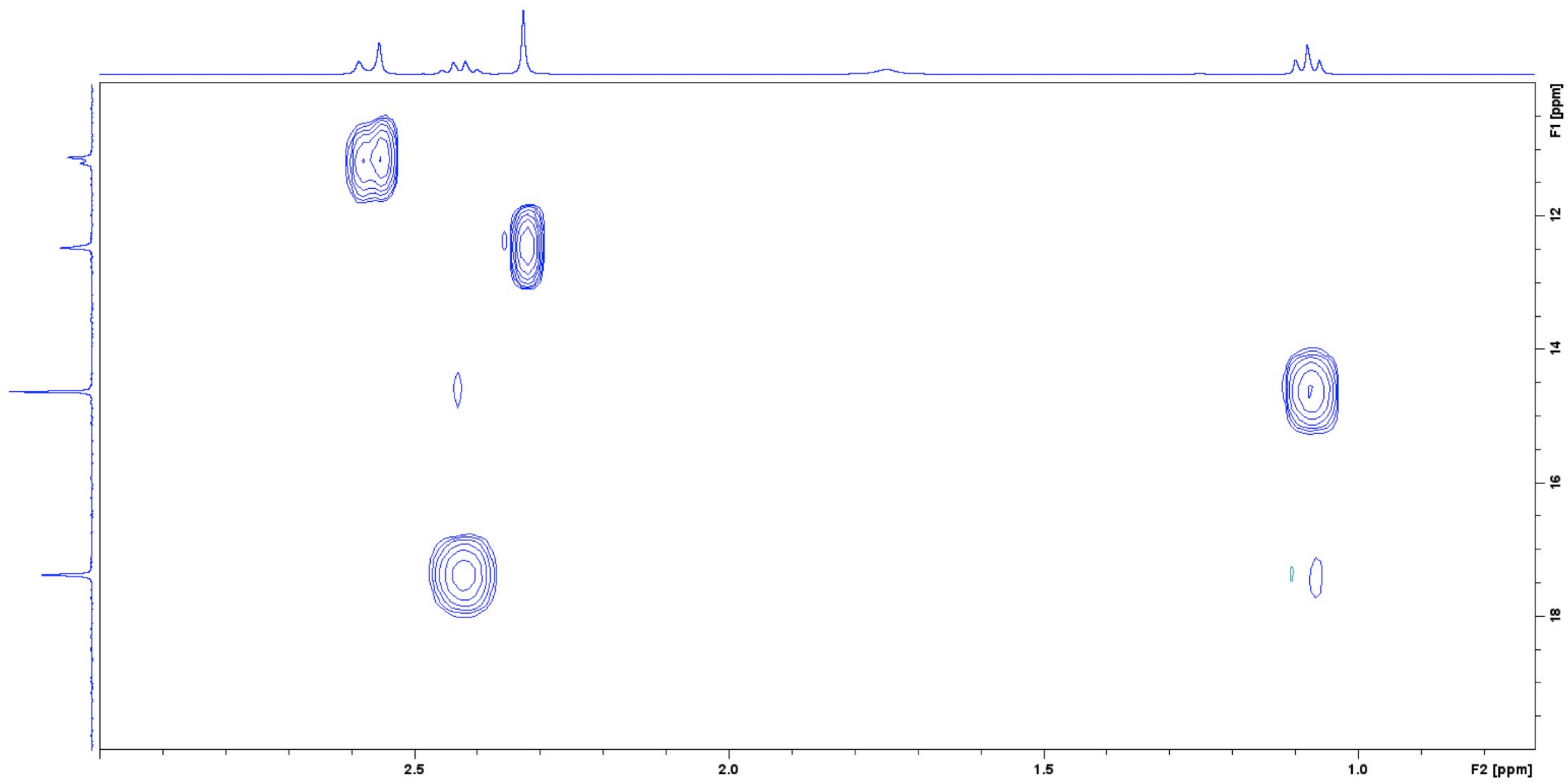

# X-RAY CHARACTERIZATION DATA OF DYE 1

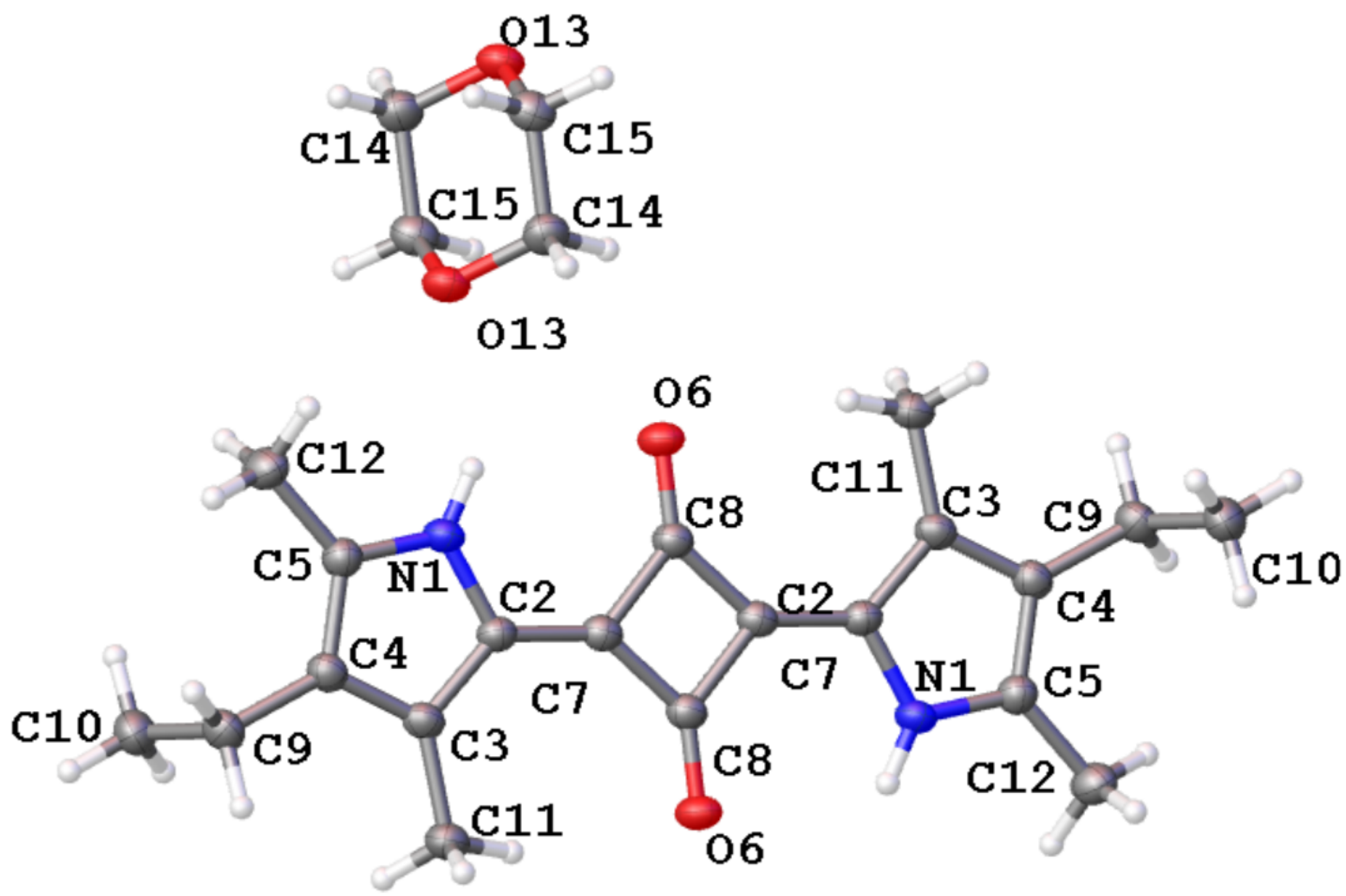


X-ray model of **1•Dioxane** showing the atom labeling scheme. The model contains a mirror plane through bonds C8-O6, generating the other half of the molecule as shown.

## Crystal data and structure refinement for 1•Dioxane

| | |
|---|---|
| Empirical formula | $C_{12}H_{15}NO_2$ |
| Formula weight | 205.258 |
| Temperature/K | 150.00(10) |
| Crystal system | triclinic |
| Space group | P-1 |
| a/Å | 4.6302(5) |
| b/Å | 11.1157(13) |
| c/Å | 11.5022(10) |
| α/° | 111.234(10) |
| β/° | 92.115(9) |
| γ/° | 96.729(9) |
| Volume/Å$^3$ | 545.99(11) |
| Z | 2 |
| $\rho_{calc}$ g/cm$^3$ | 1.249 |
| μ/mm-1 | 0.685 |
| F(000) | 220.7 |
| Crystal size/mm$^3$ | 0.9 × 0.08 × 0.05 |
| Radiation | Cu Kα (λ = 1.54184) 2Θ range for data collection/° 8.62 to 145.3 |
| Index ranges | -4 ≤ h ≤ 5, -13 ≤ k ≤ 13, -14 ≤ l ≤ 13 |
| Reflections collected | 3872 |
| Independent reflections | 2101 [$R_{int}$ = 0.0366, $R_{sigma}$ = 0.0465] Data/restraints/parameters |
| Goodness-of-fit on $F^2$ | 1.091 |

Final R indexes [I>=2σ (I)] $R_1$ = 0.0593, $wR_2$ = 0.1559
Final R indexes [all data] $R_1$ = 0.0687, $wR_2$ = 0.1704
Largest diff. peak/hole / e Å-3 0.36/-0.25

## Bond Lengths for 1•Dioxane

| Atom | Atom | Length/Å | Atom | Atom | Length/Å |
|---|---|---|---|---|---|
| O6 | C8 | 1.230(2) | C4 | C3 | 1.397(3) |
| O13 | C15 | 1.427(2) | C4 | C9 | 1.504(3) |
| O13 | C14 | 1.437(3) | C8 | C7$^1$ | 1.465(2) |
| N1 | C2 | 1.391(2) | C8 | C7 | 1.473(3) |
| N1 | C5 | 1.353(2) | C5 | C12 | 1.489(3) |
| C2 | C7 | 1.396(3) | C3 | C11 | 1.491(3) |
| C2 | C3 | 1.413(2) | C9 | C10 | 1.526(3) |
| C4 | C5 | 1.398(3) | C15 | C14$^2$ | 1.506(3) |

$^1$2-X,2-Y,1-Z; $^2$1-X,2-Y,-Z

## Bond Angles for 1•Dioxane

| Atom | Atom | Atom | Angle/˚ |
|---|---|---|---|
| C14 | O13 | C15 | 109.80(15) |
| C5 | N1 | C2 | 109.63(16) |
| C7 | C2 | N1 | 120.56(17) |
| C3 | C2 | N1 | 106.92(16) |
| C3 | C2 | C7 | 132.49(17) |
| C3 | C4 | C5 | 107.76(17) |
| C9 | C4 | C5 | 124.88(17) |
| C9 | C4 | C3 | 127.34(18) |
| C7 | C8 | O6 | 137.47(18) |
| C7[1] | C8 | O6 | 132.77(17) |
| C4 | C5 | N1 | 108.43(16) |
| C12 | C5 | N1 | 122.07(18) |
| C12 | C5 | C4 | 129.50(18) |
| C8 | C7 | C2 | 139.23(18) |
| C8[1] | C7 | C2 | 130.52(17) |
| C4 | C3 | C2 | 107.26(16) |
| C11 | C3 | C2 | 125.71(17) |
| C11 | C3 | C4 | 127.02(17) |
| C10 | C9 | C4 | 112.99(17) |
| C14[2] | C15 | O13 | 111.00(16) |
| C15[2] | C14 | O13 | 110.52(17) |

[1]2-X,2-Y,1-Z; [2]1-X,2-Y,-Z

## Torsion Angles for 1•Dioxane.

| A | B | C | D | Angle/˚ |
|---|---|---|---|---|
| O6 | C8 | C7 | C2 | -1.0(3) |
| O6 | C8 | C7[1] | C2[1] | 0.2(3) |
| O6 | C8 | C7 | C8[1] | 179.4(2) |
| O6 | C8 | C7[1] | C8[1] | -179.48(19) |
| O13 | C15 | C14[2] | O13[2] | 57.92(16) |
| O13 | C14 | C15[2] | O13[2] | -57.92(16) |
| N1 | C2 | C7 | C8 | 179.21(15) |
| N1 | C2 | C7 | C8[1] | -1.3(2) |
| N1 | C2 | C3 | C4 | 0.63(16) |
| N1 | C2 | C3 | C11 | -178.11(14) |
| N1 | C5 | C4 | C3 | 0.63(17) |
| N1 | C5 | C4 | C9 | -177.65(15) |
| C2 | C7 | C8 | C7[1] | 179.6(2) |
| C2 | C7 | C8[1] | C7[1] | -179.65(18) |
| C2 | C3 | C4 | C5 | -0.78(17) |
| C2 | C3 | C4 | C9 | 177.45(14) |
| C8 | C7 | C8[1] | C7[1] | -0.0(2) |
| C8 | C7[1] | C8[1] | C7 | 0.0(2) |

[1]2-X,2-Y,1-Z; [2]1-X,2-Y,-Z

## REFERENCES


S1. M. J. Frisch, G. W. Trucks, H. B. Schlegel, G. E. Scuseria, M. A. RobbJ. R. Cheeseman, G. Scalmani, V. Barone, G. A. Petersson, H. Nakatsuji, X. Li, M. Caricato, A. V. Marenich, J. Bloino, B. G. Janesko, R. Gomperts, B. Mennucci, H. P. Hratchian, J. V. Ortiz, A. F. Izmaylov, J. L. Sonnenberg, D. Williams-Young, F. Ding, F. Lipparini, F. Egidi, J. Goings, B. Peng, A. Petrone, T. Henderson, D. Ranasinghe, V. G. Zakrzewski, J. Gao, N. Rega, G. Zheng, W. Liang, M. Hada, M. Ehara, K. Toyota, R. Fukuda, J. Hasegawa, M. Ishida, T. Nakajima, Y. Honda, O. Kitao, H. Nakai, T. Vreven, K. Throssell, J. A. Montgomery Jr., J. E. Peralta, F. Ogliaro, M. J. Bearpark, J. J. Heyd, E. N. Brothers, K. N. Kudin, V. N. Staroverov, T. A. Keith, R. Kobayashi, J. Normand, K. Raghavachari, A. P. Rendell, J. C. Burant, S. S. Iyengar, J. Tomasi, M. Cossi, J. M. Millam, M. Klene, C. Adamo, R. Cammi, J. W. Ochterski, R. L. Martin, K. Morokuma, O. Farkas, J. B. Foresman and D. J. Fox, Gaussian 16, Revision C.01; Gaussian, Inc.: Wallingford, CT, 2016.

S2. a) P. Hohenberg, W. Kohn, *Gas. Phys. Rev*., 1964, **136**, B864−B871; b) W. Kohn, L. J. Sham, *Phys. Rev*. **1965**, *140*, A1133−A1138.

S3. a) R. Ditchfield, W. J. Hehre, J. A. Pople, *J. Chem. Phys*. **1971**, *54*, 724−728; b) A. D. McLean, G. C. Chandler, *J. Chem. Phys*. **1980**, *72*, 5639−5648; c) M. J. Frisch, J. A. Pople, J. S. Binkley, *J. Chem. Phys*. **1984**, *80*, 3265−3269.

S4. A. V. Marenich, C. J. Cramer, D. G. Truhlar, *J. Phys. Chem. B* **2009**, *113*, 6378−6396.

S5. A. Balcerak, D. Kwiatkowska, J. Kabatc, *Polym. Chem*. **2022**, *13*, 220–234.

S6. P. M. Kazmaier, G. K. Hamer, R. A. Burt, *Can. J. Chem*. **1990**, *68*, 530–536.

S7. C. W. Dirk, W. C. Herndon, F. Cervantes-Lee, H. Selnau, S. Martinez, P. Kalamegham, A. Tan, G. Campos, M. Velez, J. Zyss, I. Ledoux, L-T. Cheng, *J. Am. Chem. Soc.* **1995**, *117*, 2214–2225.

S8. A. T. Rösch, S. H. M. Söntjens, J. Robben, A. R. A. Palmans, T. Schnitzer, *J. Org. Chem*., **2021**, *86*, 13100-13103.

S9. Y. Kubota, M. Nakazawa, J. Lee, R. Naoi, M. Tachikawa, T. Inuzuka, K. Funabiki, M. Matsui, T. Kim, *Org. Chem. Front*., **2021**, *8*, 6226–6243.

S10. L. E. McNamara, T. A. Rill, A. J. Huckaba, V. Ganeshraj, J. Gayton, R. A. Nelson, E. A. Sharpe, A. Dass, N. I. Hammer, J. H. Delcamp, *Chem. Eur. J.* **2017**, *23*, 12494–12501.

S11. D. Keil, H. Hartmann, *Dyes Pigments* **2001**, *49*, 161–179.

S12. W. Li, H. Guo, Z. Wang, G. Dong, *J. Phys. Chem. C* **2017**, *121*, 15333–15338.

S13. C. Reichardt, *Chem. Rev.* **1994**, *94*, 2119–2358.

S14. M. J. Kamlet, J. L. Abboud, R. W. Taft, *J. Am. Chem. Soc.* **1977**, *99*, 6027–6038.

S15. a) A. A. Maryott, E. R. Smith, Table of dielectric Constants of Pure Liquids, National Bureau of Standards Circular 514, Issued August 10, **1951**; b) https://www.govinfo.gov/content/pkg/GOVPUB-C13-68b1154fcd941d0c6d982cb1dcd5b632/pdf/GOVPUB-C13-68b1154fcd941d0c6d982cb1dcd5b632.pdf

S16. P. Sarkar, R. Luchowski, S. Raut, N. Sabnis, A. Remaley, A. G. Lacko, S. Thamake, Z. Gryczynski, I. Gryczynski, *Biophys. Chem*. **2010**, *153 (1)*, 61–69.

S17. A. Sillen, Y. Engelborghs, The correct used of “average” fluorescence parameters. *Photochem. Photobiol.* **1998**, *67*, 475–486.

S18. H. Doan, M. Castillo. M. Bejjani, Z. Nurekeyev, S. V. Dzyuba, I. Gryczynski, Z. Gryczynski, S. Raut, Solvatochromic dye LDS 798 as microviscosity and pH probe. *Phys. Chem. Chem. Phys.* **2017**, *19*, 29934–29939.

S19.https://www.sigmaaldrich.com/deepweb/assets/sigmaaldrich/marketing/global/documents/614/456/labbasics_pg144.pdf
